# Implementation of a heart failure educational intervention for patients with recent admissions for acute decompensated heart failure

**DOI:** 10.3389/fcvm.2023.1133988

**Published:** 2023-05-05

**Authors:** Sarah Stahlman, Stephanie Huizar-Garcia, Justina Lipscomb, Christopher Frei, Ashley Oliver

**Affiliations:** ^1^ South Texas Veterans Health Care System, Veterans Health Administration, United States Department of Veterans Affairs, San Antonio, TX, United States; ^2^ College of Pharmacy, The University of Texas at Austin, Austin, TX, United States; ^3^ School of Medicine, The University of Texas Health Science Center San Antonio, San Antonio, TX, United States

**Keywords:** heart failure, education, pharmacotherapy, decompensated, readmission, emergency room (ER)

## Abstract

**Purpose:**

This study examined whether implementation of a heart failure (HF) education class targeted at patients and their caregivers decreased worsening HF, emergency department (ED) visits and hospital admissions, and improved patient quality of life and confidence in disease state management.

**Methods:**

Patients with HF and a recent hospital admission for acute decompensated heart failure (ADHF) were offered an educational course covering HF pathophysiology, medications, diet, and lifestyle modifications. Patients completed surveys before and 30 days after completion of the educational course. Outcomes of participants at 30 and 90 days after class completion were compared against outcomes for the same patients at 30 and 90 days prior to course attendance. Data was collected using electronic medical records, in-person during the class, and during a phone follow-up.

**Results:**

The primary outcome was a composite of hospital admission, ED visit, and/or outpatient visit for HF at 90 days. A total of twenty-six patients attended classes between September 2018 and February 2019 and were included in the analysis. Median age was 70 years, and most patients were white. All patients were American College of Cardiology/American Heart Association (ACC/AHA) Stage C and a majority had New York Heart Association (NYHA) Class II or III symptoms. Median left ventricular ejection fraction (LVEF) was 40%. The primary composite outcome occurred significantly more frequently in the 90 days prior to class attendance than in the 90 days following attendance (96% vs. 35%, *p* < 0.01). Likewise, the secondary composite outcome occurred significantly more frequently in the 30 days before class attendance than in the 30 days following (54% vs. 19%, *p* = 0.02). These results were driven by a decrease in admissions and ED visits for HF symptoms. Survey scores related to patient HF self-management practices and patient confidence in ability to self-manage HF increased numerically from baseline to 30 days after class attendance.

**Conclusion:**

Implementation of an educational class for HF patients improved patient outcomes, confidence, and ability to self-manage HF. Hospital admissions and ED visits also decreased. Adoption of such a course might help to decrease overall health care costs and improve patient quality of life.

## Introduction

Heart failure (HF) is a complex clinical syndrome that results from structural or functional impairment of the heart and leads to an impaired ability to fill and/or eject blood from the heart to meet the body's metabolic needs (1–3). HF is a common condition in the United States (US) with a lifetime risk of 20% in those age ≥40 years, which increases with advancing age and occurs more frequently in men than in women (1). Thus, the predominantly older, male patients with multiple comorbidities served by the South Texas Veterans Health Care System (STVHCS), are especially at risk for HF.

When untreated or poorly managed, HF can result in increased emergency department (ED) visits and hospitalizations and decreased quality of life for patients. There are over one million hospitalizations annually in the US with HF as a primary diagnosis and 25% of these patients are readmitted within the first month following discharge (1). The economic burden of disease is also large, with total costs exceeding $30 billion annually in the US alone (1) Improvements in management of HF, including improved patient. Adherence and decreased hospitalizations could substantially lessen the financial and social burdens associated with HF.

Current clinical practice guidelines recommend tailored, one-on-one patient education prior to discharge following any hospitalization for acute decompensated HF (ADHF) (2, 4). These guidelines underscore the importance of a multidisciplinary approach to care that emphasizes structured follow-up including education (2, 4). At the STVHCS, education is provided prior to discharge for most hospitalized patients; however, no formalized outpatient educational component existed prior to our study. Thus, we aimed to implement a structured outpatient educational component with the goal of improving patient knowledge and understanding of their disease, increasing patient comfort and confidence in self-management of disease, improving adherence to diet and medications, and improving patient quality of life. The goal of this initiative was a subsequent decrease in ED visits, hospital admissions, and outpatient visits reporting increased HF symptoms (e.g., increased shortness of breath, orthopnea, edema, weight gain). Additionally, decreasing hospitalizations, clinic visits, and ED visits for HF may also decrease overall costs to the health care system.

## Methods

This was a prospective cohort study of patients enrolled in a new HF education class at STVHCS. Patients served as their own controls, and outcomes at 30- and 90-days prior to class attendance were compared to outcomes at 30- and 90-days following class attendance. Veterans aged 18 years and older discharged from STVHCS with a primary discharge diagnosis of ADHF in the previous 90 days were eligible for inclusion. Patients were excluded if they were unable or unwilling to attend the class, were discharged to a skilled nursing facility, or were enrolled in hospice. Potential participants were identified by review of recent HF admissions data and by provider referral. Potential participants were contacted by a study investigator to offer voluntary enrollment in the class, and if patients agreed, they were scheduled to attend on a date convenient to them.

Included patients attended a one-time, two hour, HF education class alone or with family members or caregivers (attendance of others was based on patient choice). Upon arrival at the class, patients completed questionnaires on diet, exercise, medications, home blood pressure, heart rate, and weight measurements, smoking history, and alcohol and drug use. Attendees also completed the Self-Care Heart Failure Index (SCHFI) survey, a validated tool used to measure self-care practices in HF patients (5). This survey was used as the “pre-class,” or baseline, survey data. Vital signs were obtained during class and included weight, blood pressure, and heart rate. Topics covered during the class included an explanation of what HF means, medications that patients with HF might be prescribed (including brand and generic name, mechanism, common side effects, and basic monitoring parameters), nutrition for HF presented by a clinical dietician, and an introduction to the STVHCS Whole Health Program. Nutritional education focused mainly on low sodium diet, fluid restriction, and Dietary Approach to Stop Hypertension (See Supplementary Materials for educational slides used). During the class, patients were provided with informational handouts and home vital signs log sheets. All handouts and presentations were approved by the STVHCS Education Department prior to use and were designed to be appropriate for a fifth grade reading level. Following the group presentation, each patient met individually with a cardiology-trained Clinical Pharmacy Practitioner (CPP) to review medications, vital signs, diet, exercise, and set goals. All patient and support person questions were answered throughout the class.

**Figure 1:**
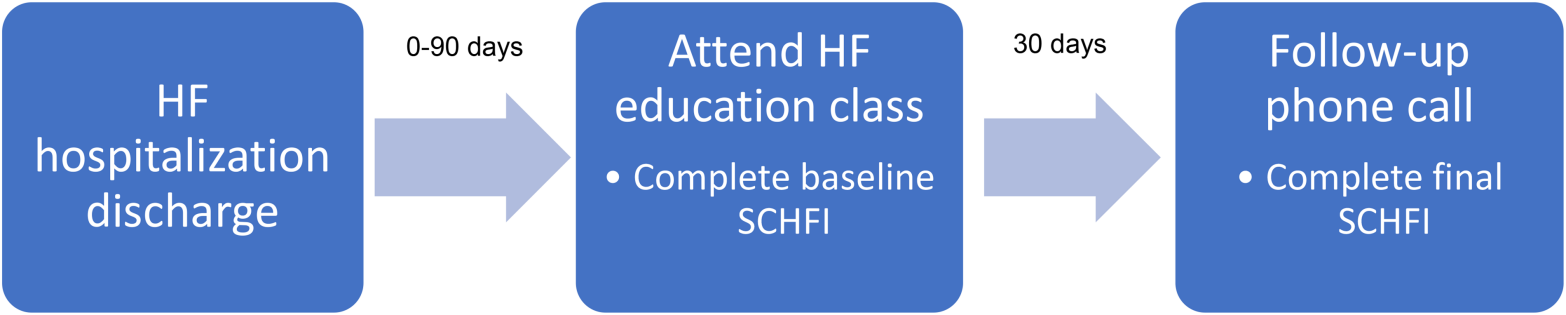
Study timeline.

If necessary, medications were initiated, adjusted, or discontinued by the CPP during individual meetings. Adjustments could be made directly by the CPP or could be suggested by alerting a primary or cardiology provider. Referrals were made to other programs or providers as needed. Patients who were identified as needing close follow-up were offered enrollment in an outpatient clinic specializing in HF and were scheduled for follow-up with a Cardiology CPP. All patients received a 30-day follow-up call from a study team member who administered the SCHFI survey. Figure 1 displays the time flow of the studied intervention from hospital discharge to final follow-up phone call.

The primary outcome was a composite of hospital admission for HF, ED visit for HF symptoms, and outpatient visits reporting uncontrolled or worsening HF symptoms at 90 days (including a broad range of symptoms such as shortness of breath, edema, or requirement of diuretic dose adjustment based on provider notes). Secondary outcomes included the composite outcome at 30 days, each individual component of the primary composite outcome, overall 30-day readmission rates for HF at STVHCS, change in patient SCHFI survey scores in sections assessing confidence in disease state management and self-management strategies, and number of pharmacist interventions made during class.

Baseline characteristics and study outcome data were collected by electronic medical record chart review. All data collected were used to analyze primary and secondary outcomes. Baseline characteristics collected included age, sex, marital status, race, ethnicity, weight, body mass index, blood pressure, heart rate, LVEF, duration of HF diagnosis, New York Heart Association (NYHA) HF class, ACC/AHA HF stage, comorbid diagnoses, smoking status, and prescriptions for select cardiovascular medications (e.g., diuretics, antihypertensives, antiplatelets, anticoagulants, and statins). Based on baseline data collection, a MAGGIC (Meta-Analysis Global Group in Chronic Heart Failure) score was calculated to assess overall cohort risk of mortality at 1 and 3 years (6). Primary and secondary outcome data at 30- and 90-days prior to class attendance were collected and compared to outcomes for the same patient at 30- and 90-days after class participation. Impact of the class on STVHCS system-wide HF outcomes was assessed by comparison of 30-day readmission rates for HF at the STVHCS in the quarter prior to implementation of the course and the quarter after implementation of the course. SCHFI survey scores before and 30 days following class attendance were compared for the same patient. Survey data for patients were linked using a coding system for deidentification purposes.

Baseline characteristics were analyzed using descriptive statistics. Primary and secondary outcomes were compared using bivariable statistics including Student's *t*-test, Wilcoxon Rank Sum, Chi-square test, Fisher's exact test, or paired versions of these tests, as appropriate. A *p*-value of <0.05 was considered to be statistically significant.

This study was approved by the UT Health San Antonio Institutional Review Board and by the Veterans Affairs Research & Development committee as non-regulated research.

## Results

Overall, twenty-six patients attended class between September 2018 and February 2019 and were included in the outcome analyses. Six patients failed to answer all survey questions, and were therefore excluded, leaving twenty patients for the survey analysis.

Table 1 contains the baseline characteristics. Median age was 70 years, most patients were white, and most were married. Most patients were NYHA Class II or III, and all patients were ACC/AHA Stage C. Patients had reduced ejection fraction, with a median LVEF of 40%. 73% of patients were on a beta-blocker and 92% were on a loop diuretic at the time of class attendance. Median MAGGIC score was 20 points, indicating an estimated 1-year mortality rate of 12.2% and 3-year mortality rate of 29.2% (6). Scores were not able to be calculated for four patients due to lack of information on date of HF diagnosis.

**Table 1:** Baseline characteristics, *n* = 26.

Age (median, IQR)	70 (67–73)
Duration of HF in days (median, IQR)	387 (87–804)
Duration of HF in months (median, IQR)	13 (3–27)
NYHA Class, *n* (%)	
I	1 (4)
II	13 (50)
III	12 (46)
IV	0 (0)
ACC/AHA Stage, *n* (%)^a^	
A	0 (0)
B	0 (0)
C	24 (100)
D	0 (0)
LVEF % (median, IQR)	40 (27–55)
Married, *n* (%)	16 (62)
Race, *n* (%)	
White	21 (81)
Black	4 (15)
Unknown	1 (4)
Ethnicity, *n* (%)	
Hispanic	10 (39)
Non-Hispanic	14 (54)
Unknown	2 (8)
Weight in kg (median, IQR)	108 (81–123)
BMI in kg/m^2^ (median, IQR)	33 (26–37)
SBP, mmHg (median, IQR)	123 (106–134)
DBP, mmHg (median, IQR)	68 (60–72)
Heart rate, beats/min (median, IQR)	69 (65–80)
MAGGIC Score (median, IQR)^b^	22 (20–24)
Comorbidities, *n* (%)	
Dyslipidemia	23 (89)
Hypertension	26 (100)
CAD	17 (65)
History TIA/Stroke	4 (15)
COPD	4 (15)
Anemia	12 (46)
Arrhythmia	11 (42)
Valvular heart disease	2 (8)
Current smoker, *n* (%)	5 (19)
Medications, *n* (%)	
Beta-blocker	19 (73)
ACEI	6 (23)
ARB	9 (35)
MRA	3 (12)
ARNi	0 (0)
Ibavradine	0 (0)
Digoxin	3 (12)
Loop diuretic	24 (92)
Thiazide diuretic	3 (12)
Other antihypertensive	5 (19)
Statin	24 (92)
Aspirin	22 (85)
P2Y12 inhibitor	5 (19)
Anticoagulant	10 (39)

IQR, interquartile range; HF, heart failure; NYHA, New York Heart Association; ACC/AHA, American College of Cardiology/American Heart Association; LVEF, left ventricular ejection fraction; BMI, body mass index; SBP, systolic blood pressure; DBP, diastolic blood pressure; CAD, coronary artery disease; TIA, transient ischemic attack; ACEI, angiotensin converting enzyme inhibitor; ARB, angiotensin receptor blocker; ARNi, angiotensin receptor neprilysin inhibitor; MRA, mineralocorticoid receptor antagonist; thiazide diuretic, includes chlorthalidone, hydrochlorothiazide, or metolazone; MAGGIC, Meta-Analysis Global Group in Chronic Heart Failure.

^a^ ACC/AHA stage was unavailable for two patients.

^b^ MAGGIC score was unavailable for four patients.

The primary composite outcome (Table 2) occurred significantly more frequently in the 90 days prior to class attendance than in the 90 days following attendance (96% vs. 35%, *p* < 0.01). The secondary composite outcome also occurred more commonly 30 days prior to class attendance than in the 30 days following attendance (54% vs. 19%, *p* = 0.02). The pre-class primary composite outcome occurrence was driven by readmissions for HF, while the post-class primary composite outcome occurrence was completely due to outpatient visits for HF. Importantly, there were no 30- or 90-day readmissions observed in our study compared to a 20% readmission rate for all of STVHCS for the last two fiscal quarters of 2018. In both the 30- or 90-day post-class analysis, no patients were seen in the ED for HF symptoms or ADHF.

**Table 2:** Health outcomes, *n* = 26.

Outcomes	Pre-class	Post-class	*p*-value
Primary composite outcome (90-day)	96%	35%	<0.01
Hospital readmission for HF	92%	0%	–
Emergency department visit for HF	8%	0%	–
Outpatient visit reporting HF symptoms	50%	35%	–
Secondary composite outcome (30-day)	54%	19%	0.02
Hospital readmission for HF	23%	0%	–
Emergency department visit for HF	0%	0%	–
Outpatient visit reporting HF symptoms	35%	19%	–

HF, heart failure.

Changes in SCHFI results from baseline survey to 30-day follow-up survey can be seen in Table 3. Section A of the SCHFI survey assesses a patient's HF self-management practices, such as monitoring daily weights, monitoring edema, other symptoms of HF, and following health care provider recommendations. Possible scores for this section of the survey ranged from 10 to 40 points, with more points indicating better self-management practices. From the baseline to 30-day follow-up survey, a median increase of 5 points (IQR −7.5, 16.7) occurred. Section C of the SCHFI survey assesses a patient's confidence in ability to self-manage HF symptoms. Possible scores for this section of the survey ranged from 6 to 24 points, with more points indicating a higher level of self-confidence. From the baseline to 30-day follow-up survey, a median increase of 5.5 points (IQR −18.1, 23.6) occurred.

**Table 3:** SCHFI change from baseline to 30-days post-class, *n* = 20.

Survey section	Change, median (IQR)	*p*-value
Section A (HF self-management practices)	+5 (−8 to 17)	0.41
Section C (HF self-management confidence)	+6 (−18 to 24)	0.28

HF, heart failure; IQR, interquartile range.

An average of two interventions per patient (IQR 2–3) were made by CPPs during class meetings. Most interventions included completion of goal-setting or other non-pharmacological interventions. Other interventions included dose adjustments and one new medication addition. Multiple providers were notified for follow-up and six patients were scheduled for follow-up in CPP HF clinics.

## Discussion

This study demonstrated that attending a one-time, two hour outpatient HF education class led to a decrease in ADHF, hospitalizations, ED visits for HF symptoms, and outpatient visits with HF symptoms. This study also demonstrated that attendance at an outpatient HF education class can lead to an increase in patient ability to self-manage their disease at home. Although the increases in survey scores for Section A and C were not statistically significant, increases in self-management practices and patient confidence in ability to self-manage symptoms are clinically significant. Of note, the 90-day composite outcome after attendance at class was driven completely by outpatient visits. No patients attending the HF education class were readmitted to the hospital, or seen in the ED, for HF symptoms within the 90 days following class attendance. Improvements in survey scores may provide insight into why no patients were readmitted or seen in the ED at the 90-day outcome analysis. The decrease in overall hospitalizations at the 90-day follow-up outcome might be due to improvement in patients' ability to recognize HF symptoms early and report them to an outpatient provider. Utilization of the SCHFI score to evaluate HF patient's self-management abilities could be considered by other facilities as a component of long-term monitoring. The score is based on a scale from 1 to 5 and implementation in an electronic monitoring system could be considered by clinicians managing HF for ease of tracking. The most current scoring system can be found here: https://self-care-measures.com/project/patient-version-schfi-english/.

The study highlights the benefits of involving pharmacists in the management of HF patients, especially in the post-discharge period as pharmacists on average made multiple interventions with each patient during class meetings. The data presented is limited by lack of detailed information on which medications specifically were adjusted during the class, however the significant influence of pharmacists on improved patient care is highlighted by the individualized care available through this type of class setting.

System-wide 30-day readmission rates for HF did not change significantly before and after the implementation of the course, possibly because patients in this study represented a small sample of all the patients hospitalized for ADHF in the STVHCS. Since the system-wide HF readmission rate was 20%, while our readmission rate was 0%, expansion of this educational intervention to the entire system would have a profound impact on the number of patients readmitted for HF within 30 days. Furthermore, since hospital reimbursement is tied to this outcome, system-wide implementation of such a class has the potential to impact hospital reimbursement.

This study has several strengths, including that it is one of very few studies that have studied the effect of an interdisciplinary outpatient HF educational class on clinical outcomes (7–10). Other studies have shown a benefit with provision of patient education at time of discharge following ADHF admission (11). One group examined an outpatient educational model including one-on-one follow-up with a nurse, followed by telehealth management, and found reduced overall costs and adverse clinical outcomes (7). Another group looked at a close follow-up model which included an educational component; however, education mainly involved providing patients with a handout discussing HF and some reinforcement and follow-up visits (8). Notably, none of these studies included a group educational component and only one of them involved interdisciplinary education (9). The educational intervention studied here included pharmacists, dieticians, and health coaches. This enabled the providers to make referrals directly with other team members and to implement medication changes immediately. Another strength of this study is access to Veterans Affairs (VA) patient medical records as it was possible to get complete pre- and post-class attendance data for patients included in the study. Results of this study are most generalizable to a VA population, but use of an interdisciplinary educational course including pharmacy and nutrition components is likely to benefit all HF patients, regardless of the health system. Additionally, we note that there is a high burden of obesity within our patient population. While our nutritional counseling focused on limiting dietary sodium, others could consider additional teaching on nutritional strategies for weight loss and maintenance to further reduce long-term morbidity risk. During the class, patients were also offered follow-up appointments with nutritionist where individualized needs could be addressed more fully, which we encourage others to consider as well.

This study does have some limitations, including that it was completed in a veteran population which included mostly older, white, male patients and therefore may not be generalizable to all patient populations. As can be seen by the MAGGIC score, our selected patient population also may represent an overall sicker patient population compared to the average heart failure patient. However, given that the majority of patients with hospitalizations for ADHF are elderly and generally have a higher comorbidity burden, some generalizability remains and our results may also be useful to other VA health systems. One likely benefit of the class design in this study was the ability to refer to other providers directly from the course and to make medication changes immediately on site if indicated. This benefit may be confined to a closed health system or managed care organization similar to that see in the VA, but likely provided significant benefit to patients. Additionally, our group of patients is small, and future studies in larger groups might help provide more information on the benefits of this type of class.

The selection of patients recently hospitalized for ADHF does have the potential to slightly skew the data as we are comparing hospitalization rates only for those who were recently hospitalized. However, given the ADHF population is at very high risk for readmission and the fact that the readmission rates in our population were reduced compared to rates in the entire HF population at our facility, it is likely the class still had significant benefits. Patients who are willing to attend an educational class after discharge may be those who are generally more adherent to therapy compared to the general population; thus, their reduced hospitalization rate following class participation could be confounded by medication, lifestyle, and medical visit adherence. The exclusion of patients discharged to a nursing facility or those receiving hospice care may also skew the population as those patients are potentially sicker than patients discharged home, thus this may have caused our readmission rate to be lower than it might have been for the overall population of recent discharges. Additionally, specific reasons for patients unwilling or unable to attend class were not collected, so it is unclear if these included significant comorbidities or circumstances that might increase risk for readmission. This intervention also included the opportunity for medication adjustment by a CPP, an intervention which could have influenced rates of rehospitalization or outpatient visits. Because of this, it would likely be of greatest benefit to include medication adjustments into such a class if implemented elsewhere. Lastly, we did not collect medication dosages or dose adjustments which limits our ability to fully analyze whether patients achieved optimal dosing. At the time of the study, Entresto® was also restricted within our health system to the cardiology department, limiting the ability of the CPPs involved with the education class to initiate or adjust this medication.

In the era of increased online educational programs and with a growing focus on virtual medical care, a similar intervention might be considered in a virtual format. Based on the study presented here, it is difficult to say whether a virtual format would provide the same benefit. We acknowledge that online-learning can reach a larger group of patients and can increase access for medically underserved populations, thus a virtual class would likely be worthwhile. We feel that the benefits of our class stem largely from the interactive nature and the individualization of the education, specifically including the one-on-one patient meetings to monitor and adjust patient-specific therapies and offer follow-up as indicated. If a virtual format is preferred, we recommend maintaining the individualization to the greatest extent possible.

## Conclusion

Implementation of an educational class for HF patients improved clinical outcomes, patient confidence, and patient ability to self-manage HF. This class helped to decrease hospital readmission and ED visits and adoption of such a course might help to decrease overall health care costs and improve patient quality of life. Further research into long-term outcomes for patients attending an educational class like that proposed here would also be helpful in determining the long-term effect of a course such as this.

## Data Availability

The data analyzed in this study is subject to the following licenses/restrictions: The datasets used and/or analyzed during the current study are available from the corresponding author on reasonable request. Requests to access these datasets should be directed to Sarah Stahlman, sarah.stahlman@va.gov.
