# Supplementary material for: Implementation of a heart failure educational intervention for patients with recent admissions for acute decompensated heart failure
Source: Front Cardiovasc Med. 2023 May 5;10:1133988. doi: 10.3389/fcvm.2023.1133988 (PMC10196446; doi:10.3389/fcvm.2023.1133988)
Supplement: Supplementary file 1 [file Presentation1.pptx]

## Slide 1
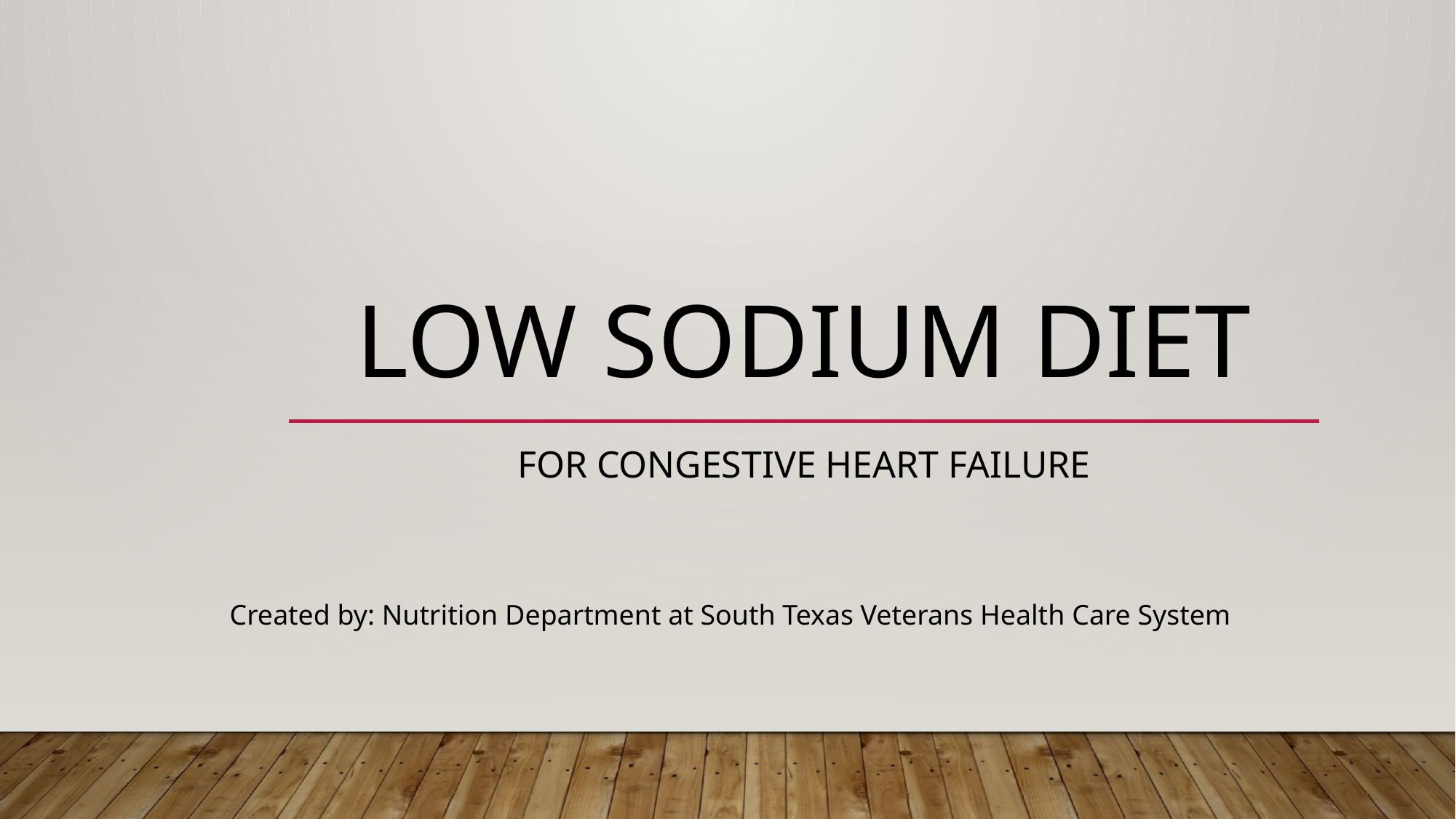

# Low Sodium Diet
For congestive heart failure
Created by: Nutrition Department at South Texas Veterans Health Care System

## Slide 2
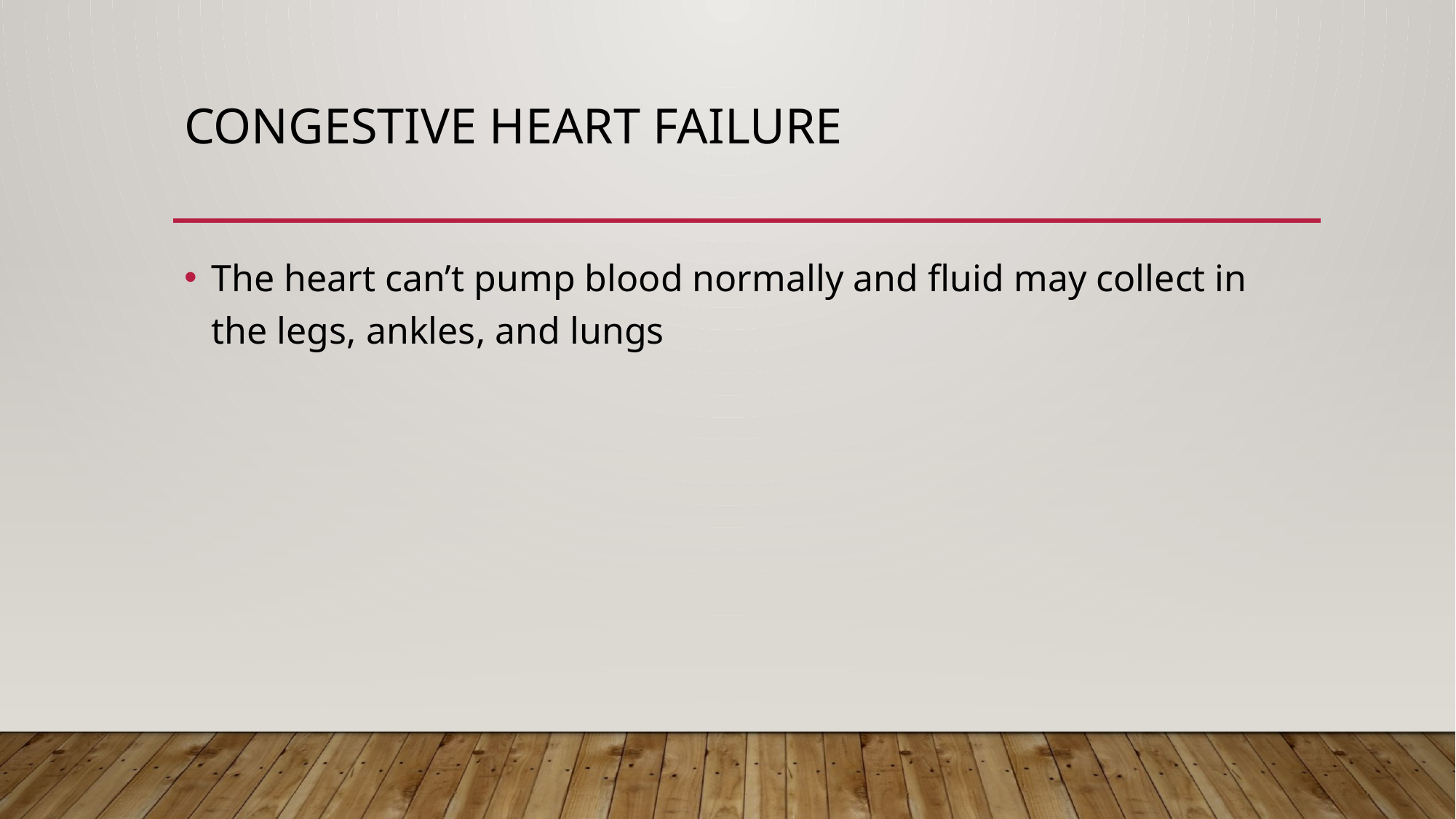

# Congestive heart failure
The heart can’t pump blood normally and fluid may collect in the legs, ankles, and lungs

## Slide 3
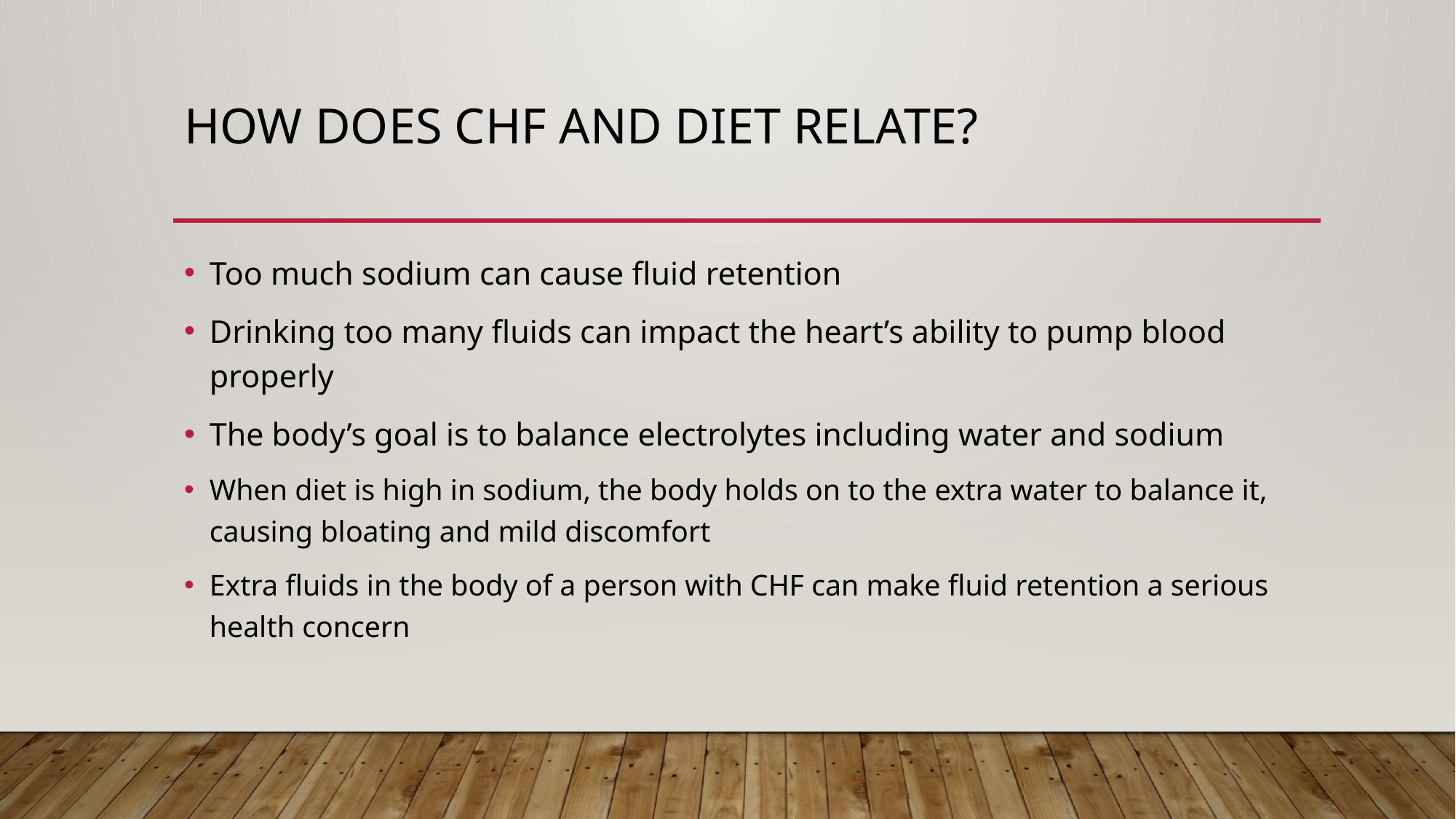

# How does CHF and diet relate?
Too much sodium can cause fluid retention
Drinking too many fluids can impact the heart’s ability to pump blood properly
The body’s goal is to balance electrolytes including water and sodium
When diet is high in sodium, the body holds on to the extra water to balance it, causing bloating and mild discomfort
Extra fluids in the body of a person with CHF can make fluid retention a serious health concern

## Slide 4
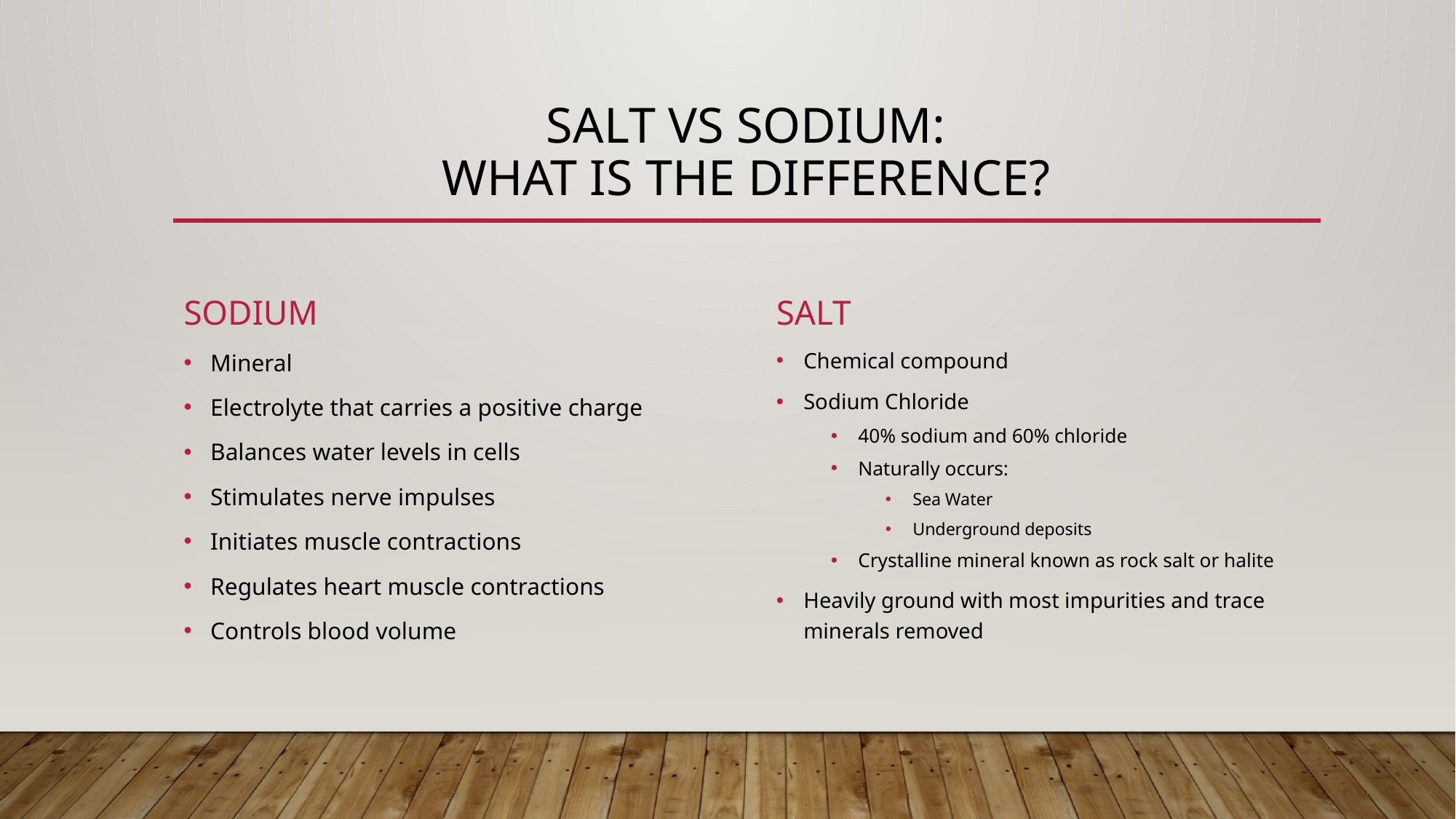

# Salt vs sodium: what is the difference?
Sodium
Salt
Chemical compound
Sodium Chloride
40% sodium and 60% chloride
Naturally occurs:
Sea Water
Underground deposits
Crystalline mineral known as rock salt or halite
Heavily ground with most impurities and trace minerals removed
Mineral
Electrolyte that carries a positive charge
Balances water levels in cells
Stimulates nerve impulses
Initiates muscle contractions
Regulates heart muscle contractions
Controls blood volume

## Slide 5
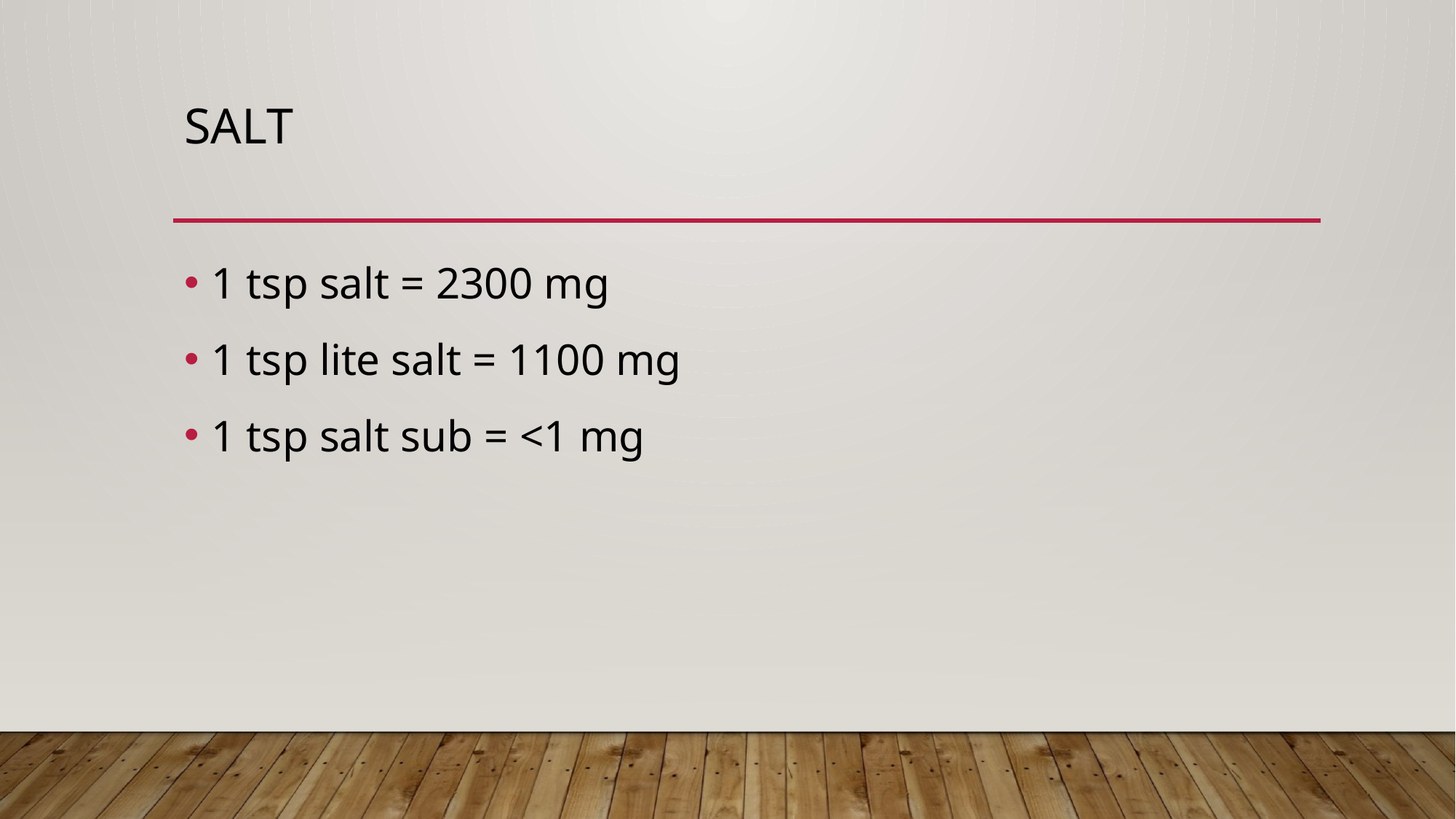

# SalT
1 tsp salt = 2300 mg
1 tsp lite salt = 1100 mg
1 tsp salt sub = <1 mg

## Slide 6
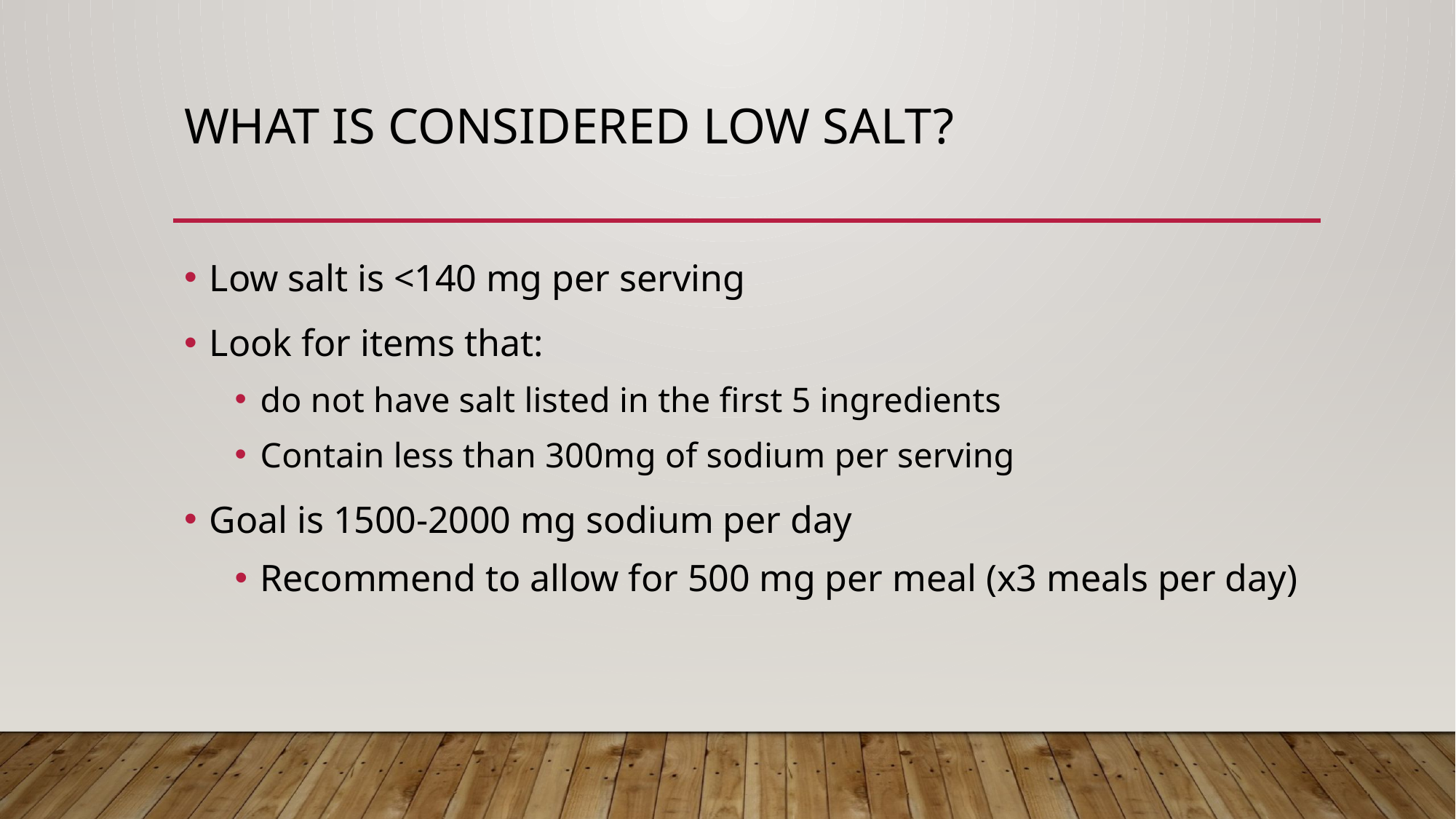

# What is considered low salt?
Low salt is <140 mg per serving
Look for items that:
do not have salt listed in the first 5 ingredients
Contain less than 300mg of sodium per serving
Goal is 1500-2000 mg sodium per day
Recommend to allow for 500 mg per meal (x3 meals per day)

## Slide 7
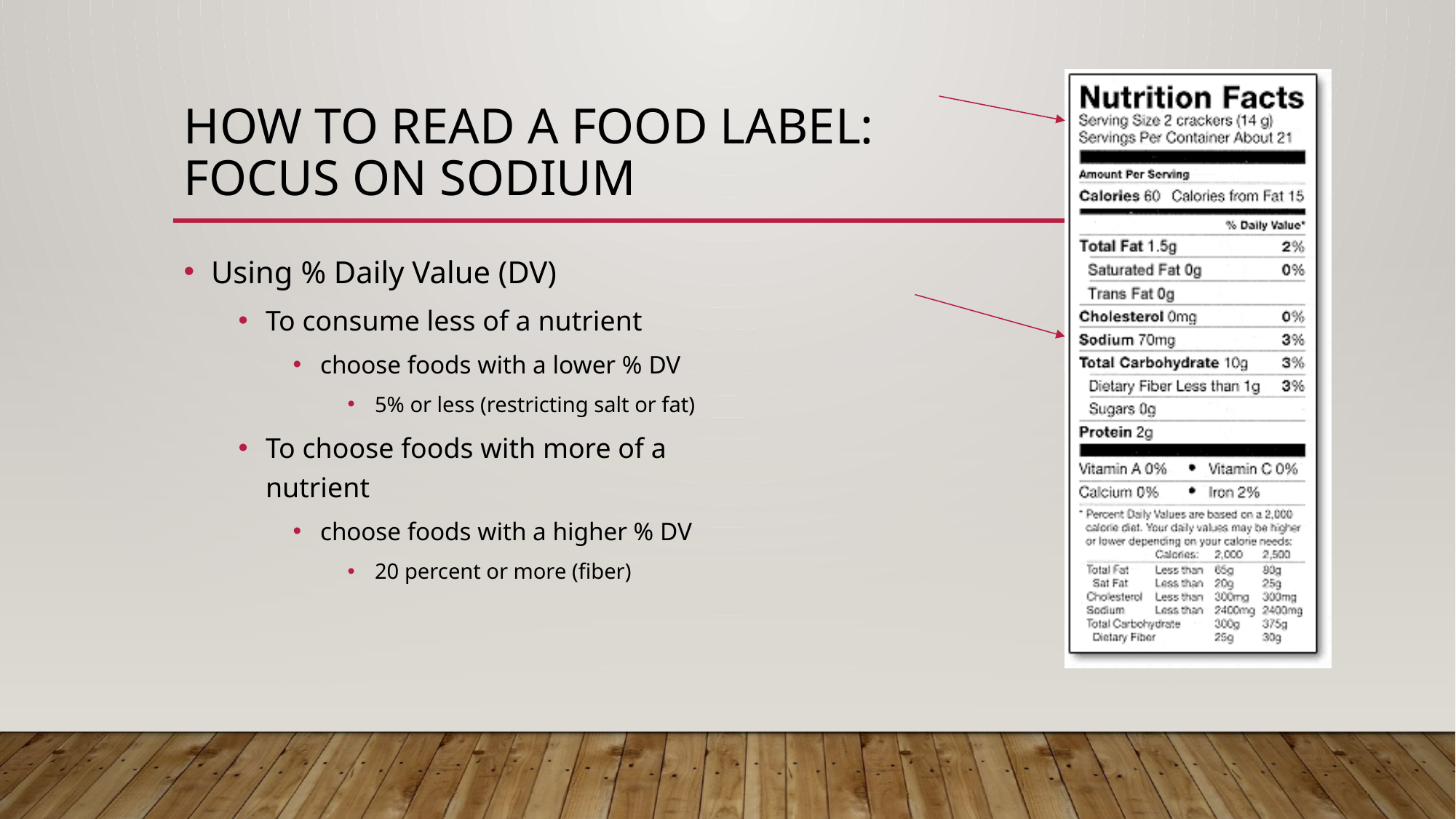

# How to read a food label: focus on sodium
Using % Daily Value (DV)
To consume less of a nutrient
choose foods with a lower % DV
5% or less (restricting salt or fat)
To choose foods with more of a nutrient
choose foods with a higher % DV
20 percent or more (fiber)

## Slide 8
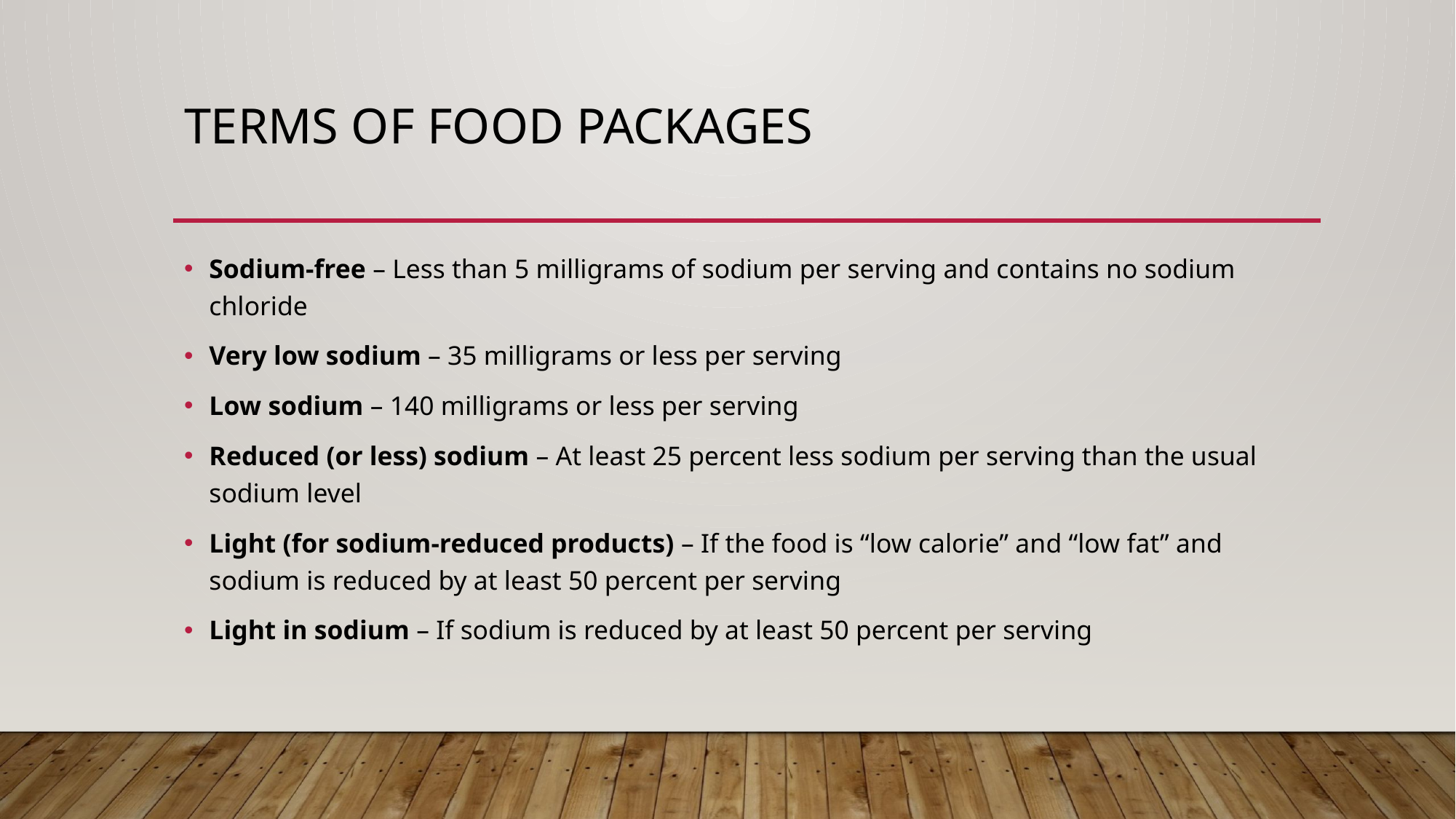

# Terms of food packages
Sodium-free – Less than 5 milligrams of sodium per serving and contains no sodium chloride
Very low sodium – 35 milligrams or less per serving
Low sodium – 140 milligrams or less per serving
Reduced (or less) sodium – At least 25 percent less sodium per serving than the usual sodium level
Light (for sodium-reduced products) – If the food is “low calorie” and “low fat” and sodium is reduced by at least 50 percent per serving
Light in sodium – If sodium is reduced by at least 50 percent per serving

## Slide 9
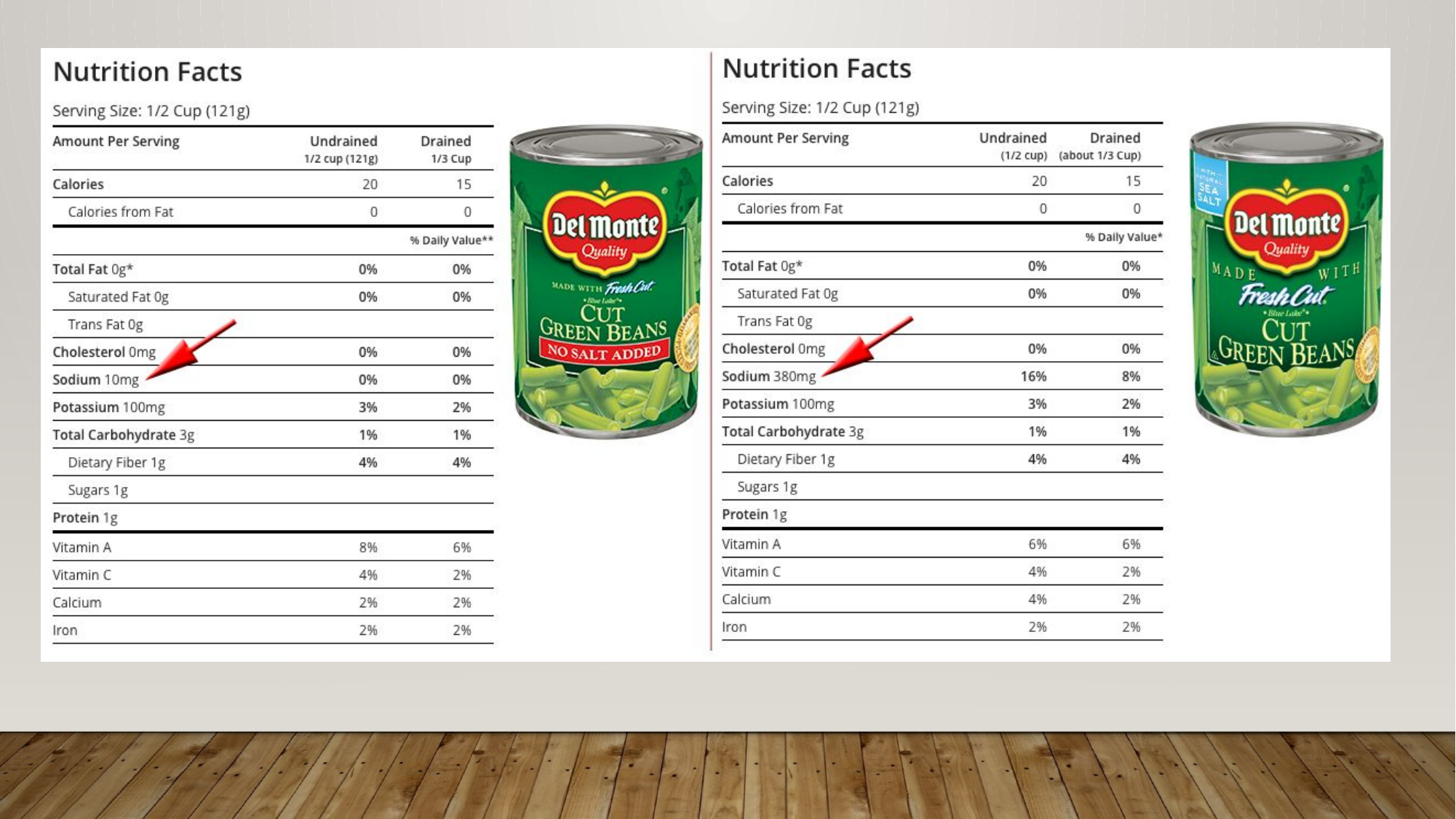

## Slide 10
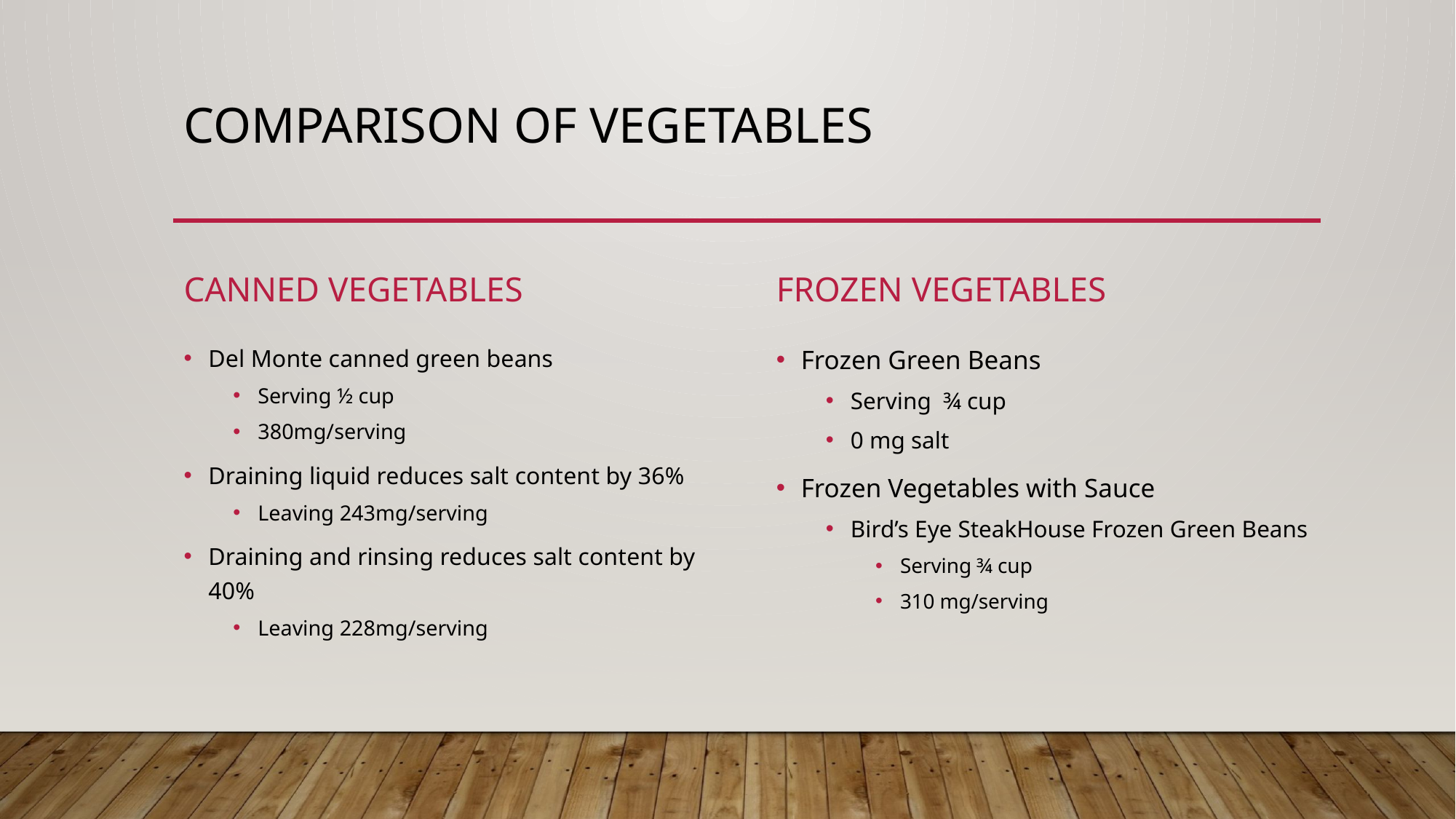

# Comparison of vegetables
Canned vegetables
Frozen vegetables
Del Monte canned green beans
Serving ½ cup
380mg/serving
Draining liquid reduces salt content by 36%
Leaving 243mg/serving
Draining and rinsing reduces salt content by 40%
Leaving 228mg/serving
Frozen Green Beans
Serving ¾ cup
0 mg salt
Frozen Vegetables with Sauce
Bird’s Eye SteakHouse Frozen Green Beans
Serving ¾ cup
310 mg/serving

## Slide 11
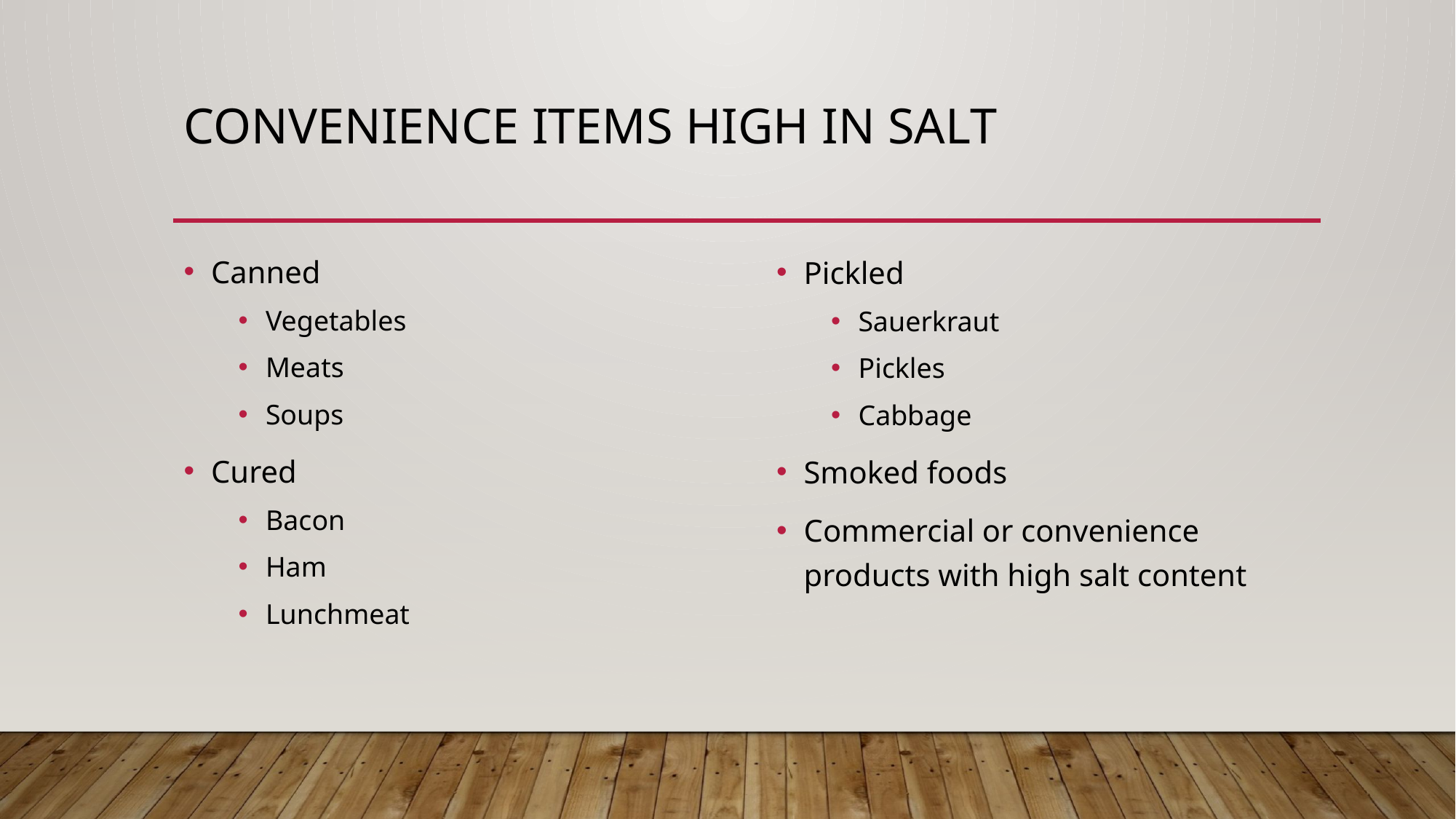

# Convenience items high in salt
Canned
Vegetables
Meats
Soups
Cured
Bacon
Ham
Lunchmeat
Pickled
Sauerkraut
Pickles
Cabbage
Smoked foods
Commercial or convenience products with high salt content

## Slide 12
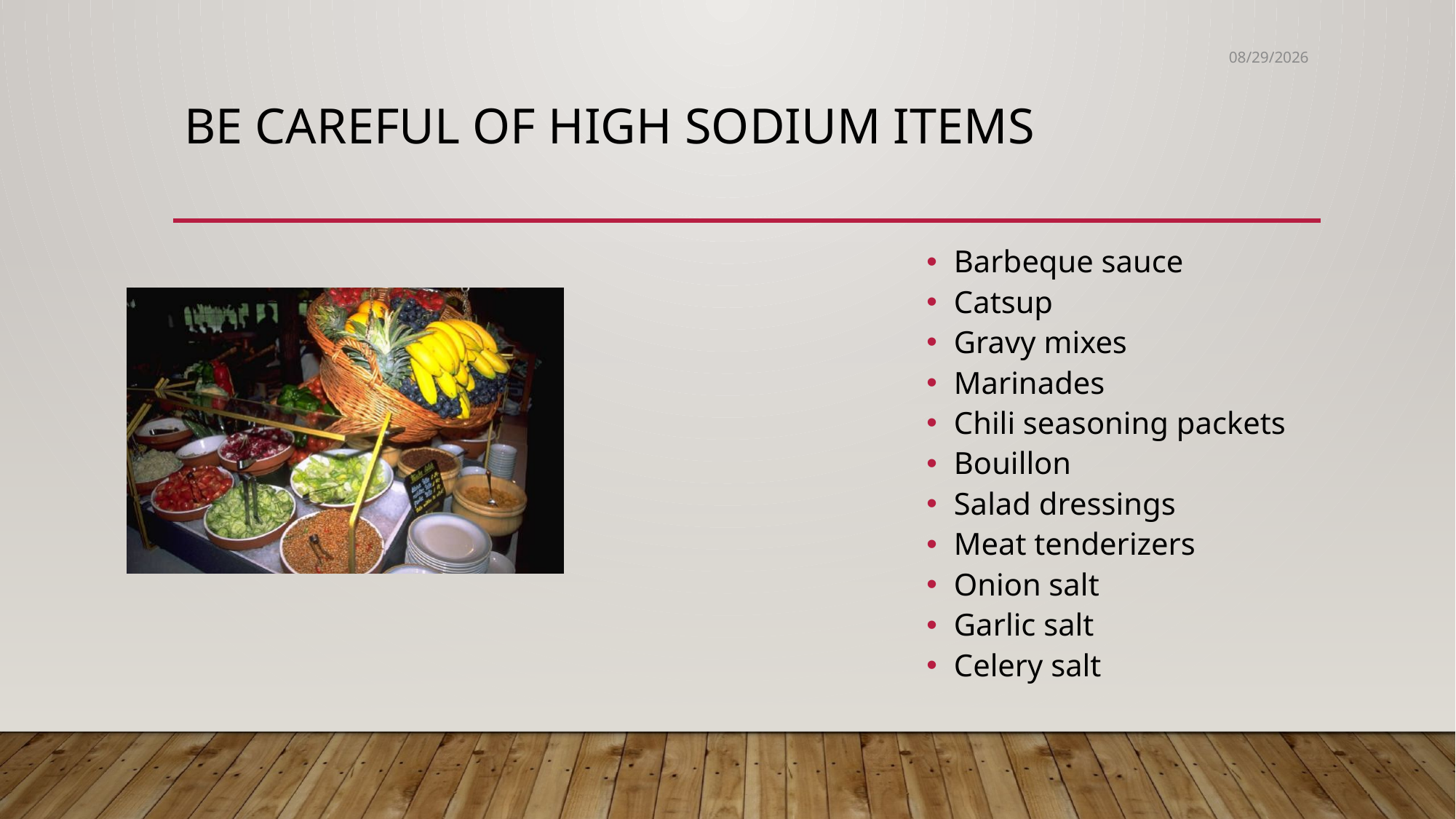

4/4/23
# Be careful of high sodium items
Barbeque sauce
Catsup
Gravy mixes
Marinades
Chili seasoning packets
Bouillon
Salad dressings
Meat tenderizers
Onion salt
Garlic salt
Celery salt

## Slide 13
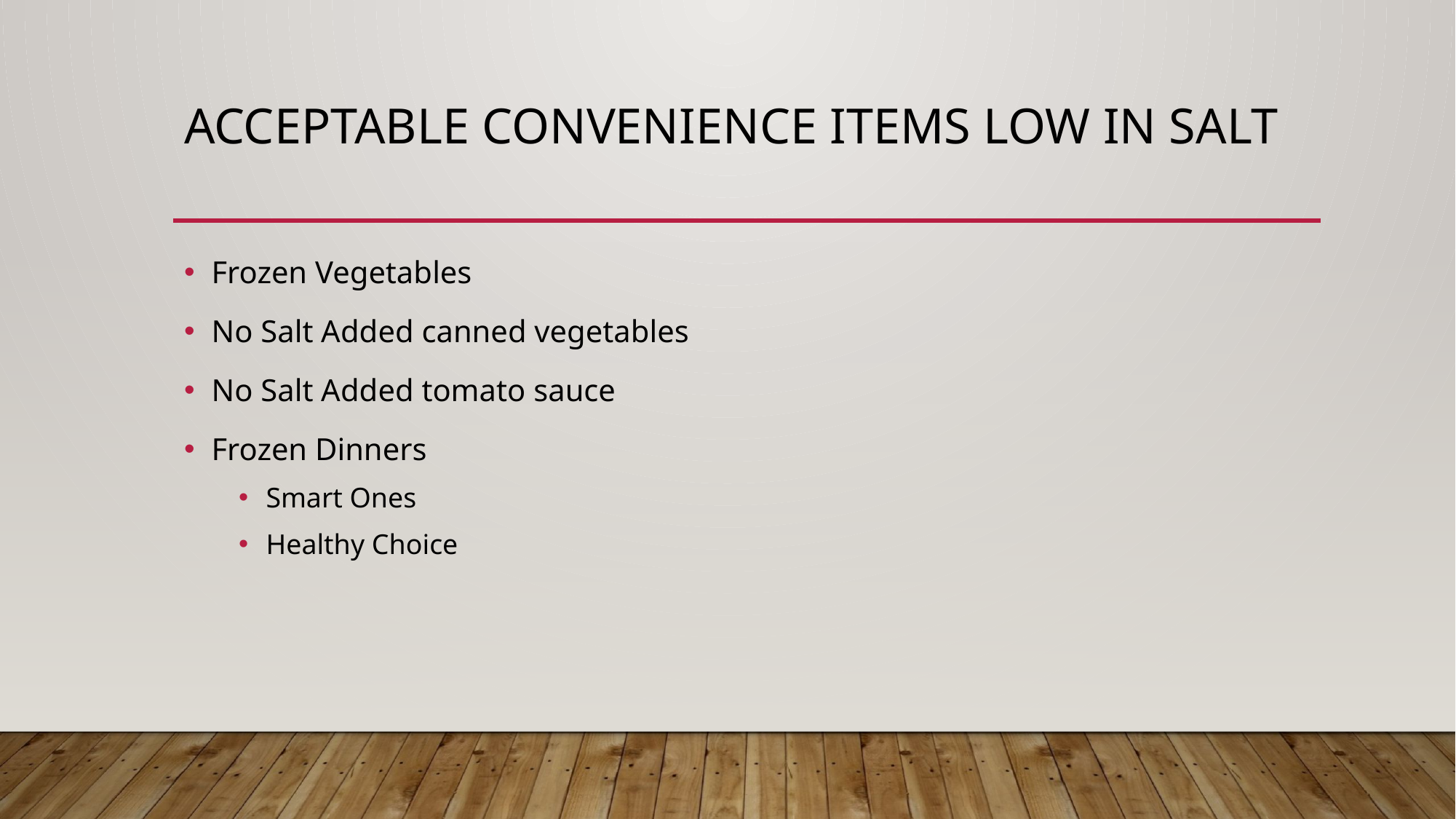

# Acceptable convenience items low in salt
Frozen Vegetables
No Salt Added canned vegetables
No Salt Added tomato sauce
Frozen Dinners
Smart Ones
Healthy Choice

## Slide 14
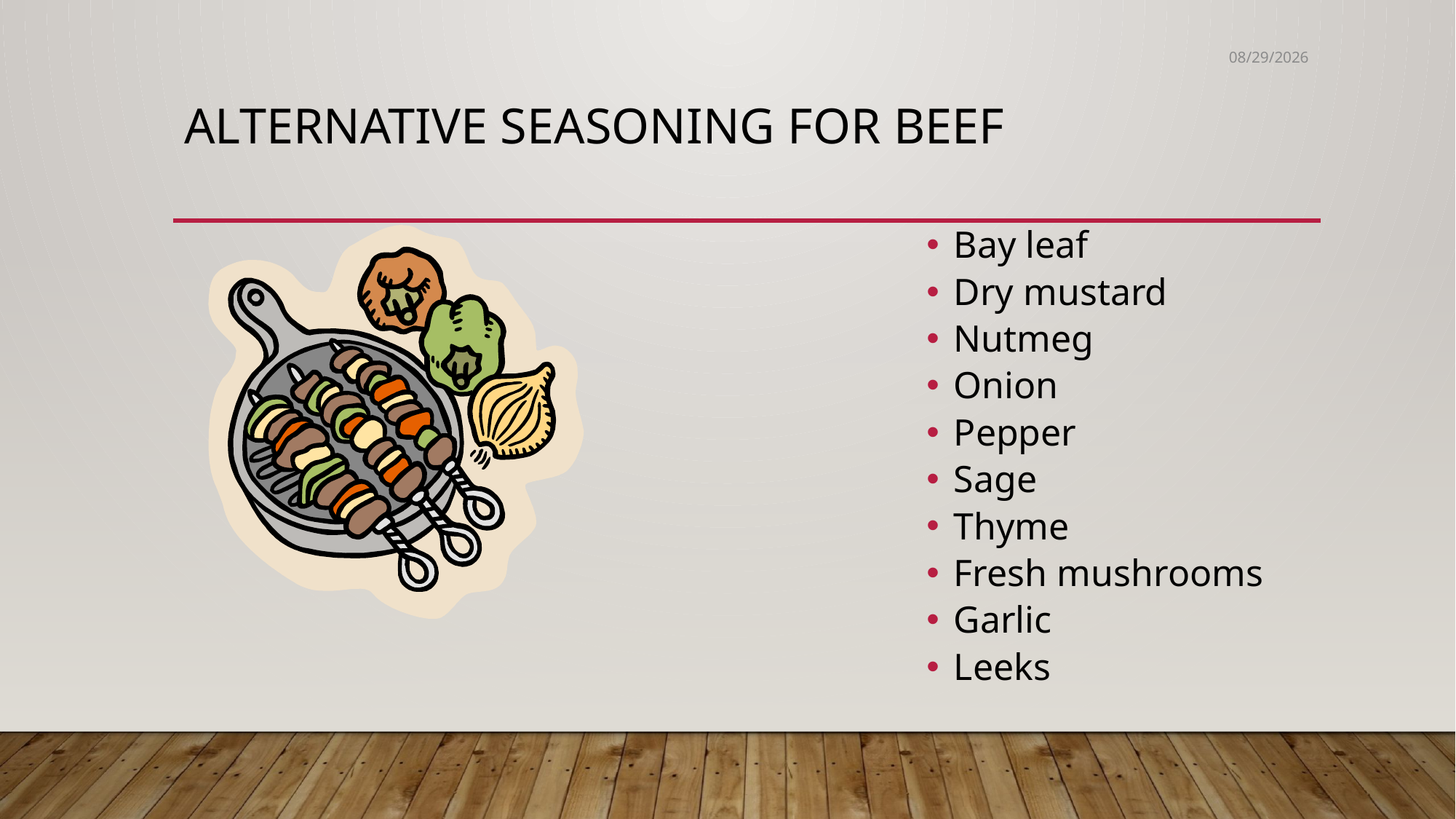

4/4/23
# Alternative seasoning for beef
Bay leaf
Dry mustard
Nutmeg
Onion
Pepper
Sage
Thyme
Fresh mushrooms
Garlic
Leeks

## Slide 15
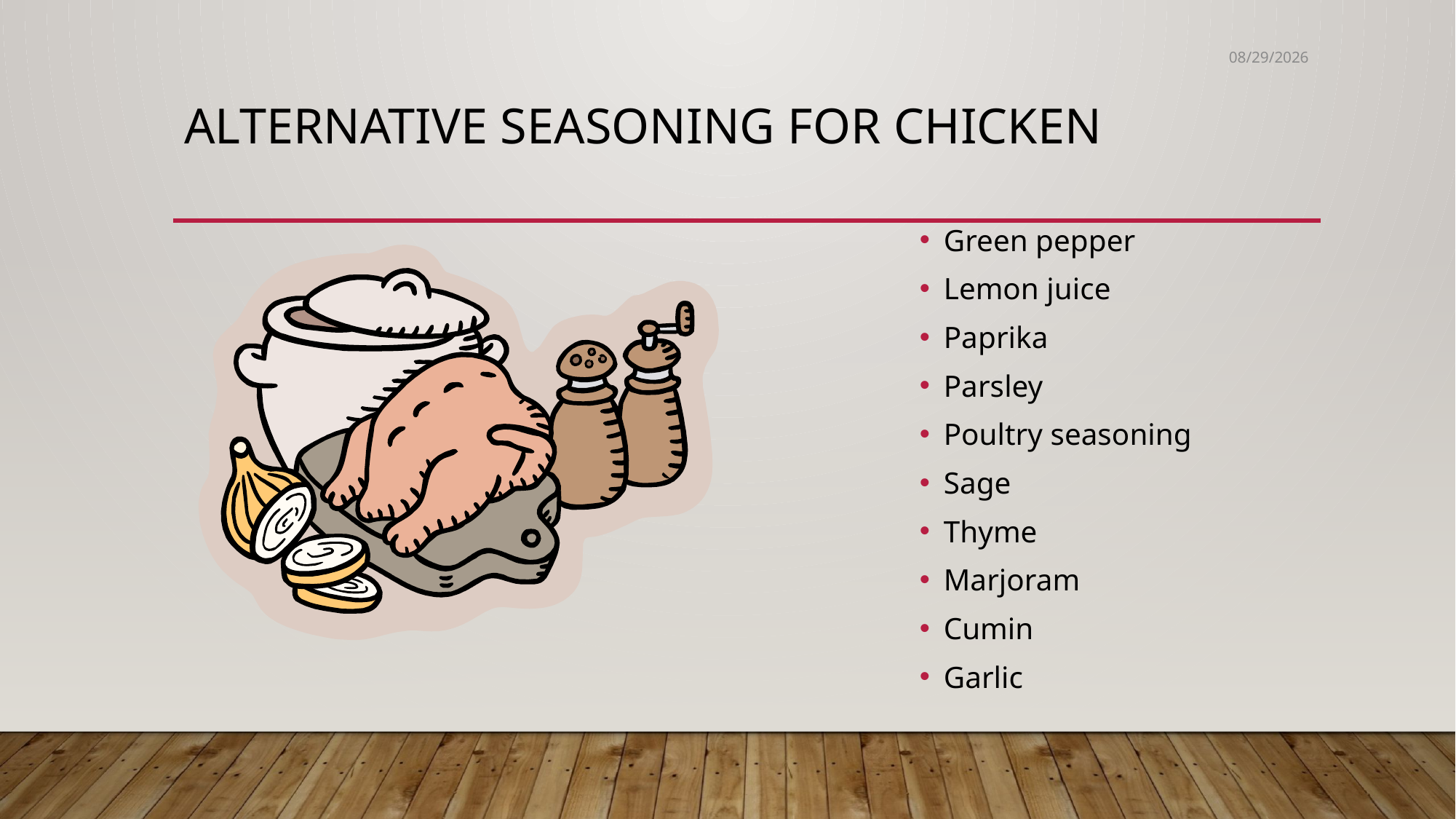

4/4/23
# Alternative seasoning for chicken
Green pepper
Lemon juice
Paprika
Parsley
Poultry seasoning
Sage
Thyme
Marjoram
Cumin
Garlic

## Slide 16
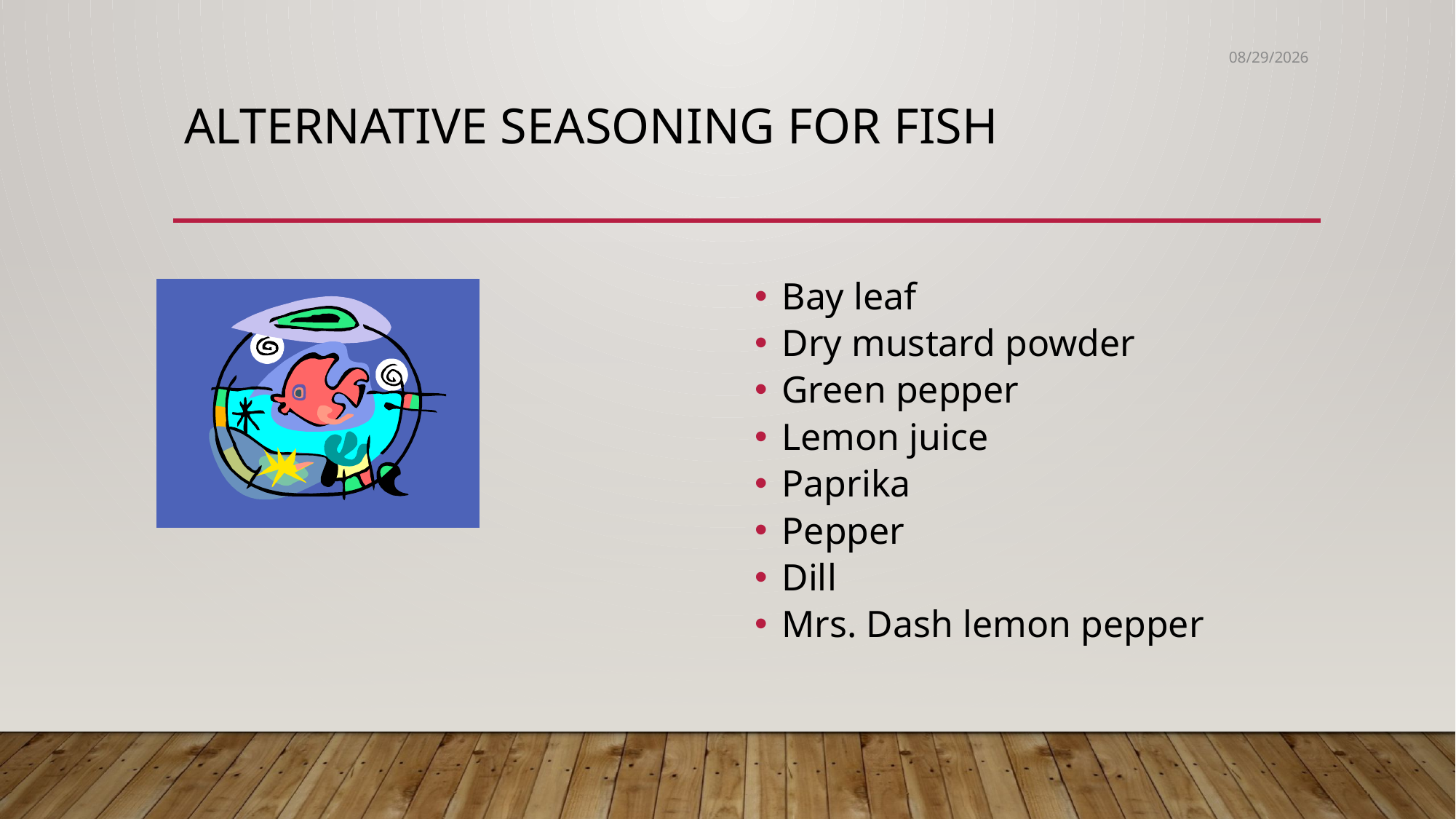

4/4/23
# Alternative seasoning for fish
Bay leaf
Dry mustard powder
Green pepper
Lemon juice
Paprika
Pepper
Dill
Mrs. Dash lemon pepper

## Slide 17
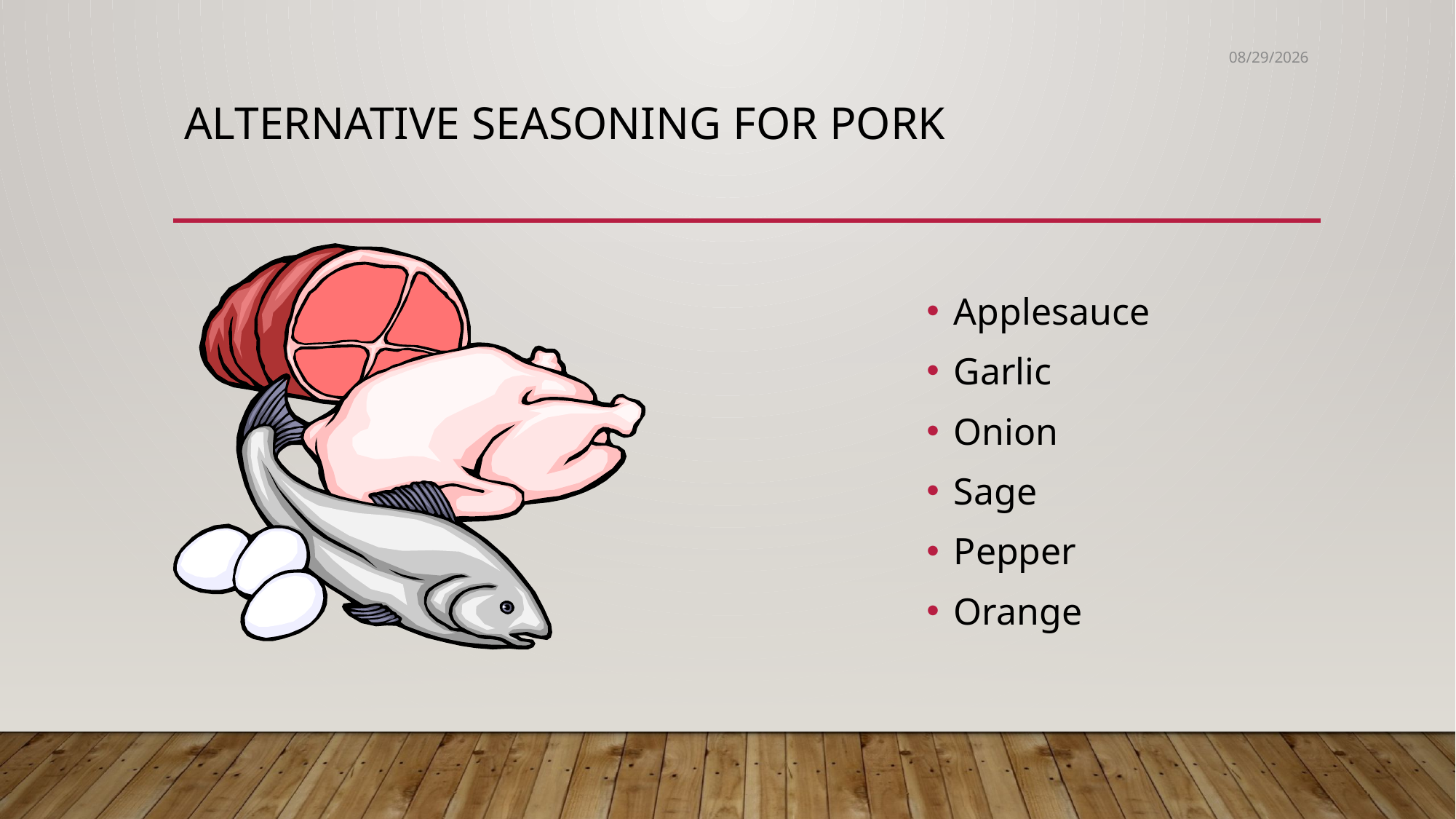

4/4/23
# Alternative seasoning for pork
Applesauce
Garlic
Onion
Sage
Pepper
Orange

## Slide 18
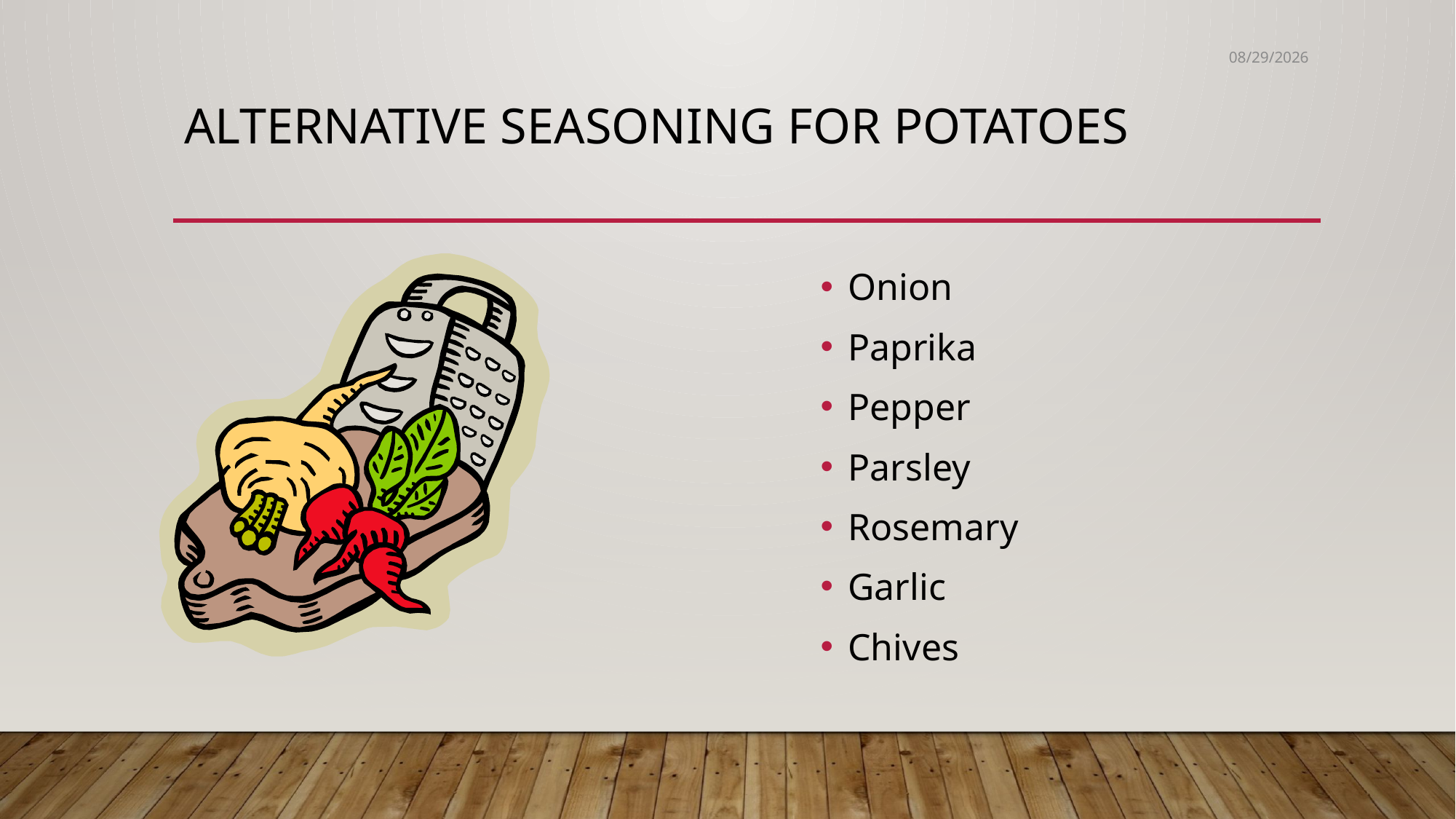

4/4/23
# Alternative seasoning for Potatoes
Onion
Paprika
Pepper
Parsley
Rosemary
Garlic
Chives

## Slide 19
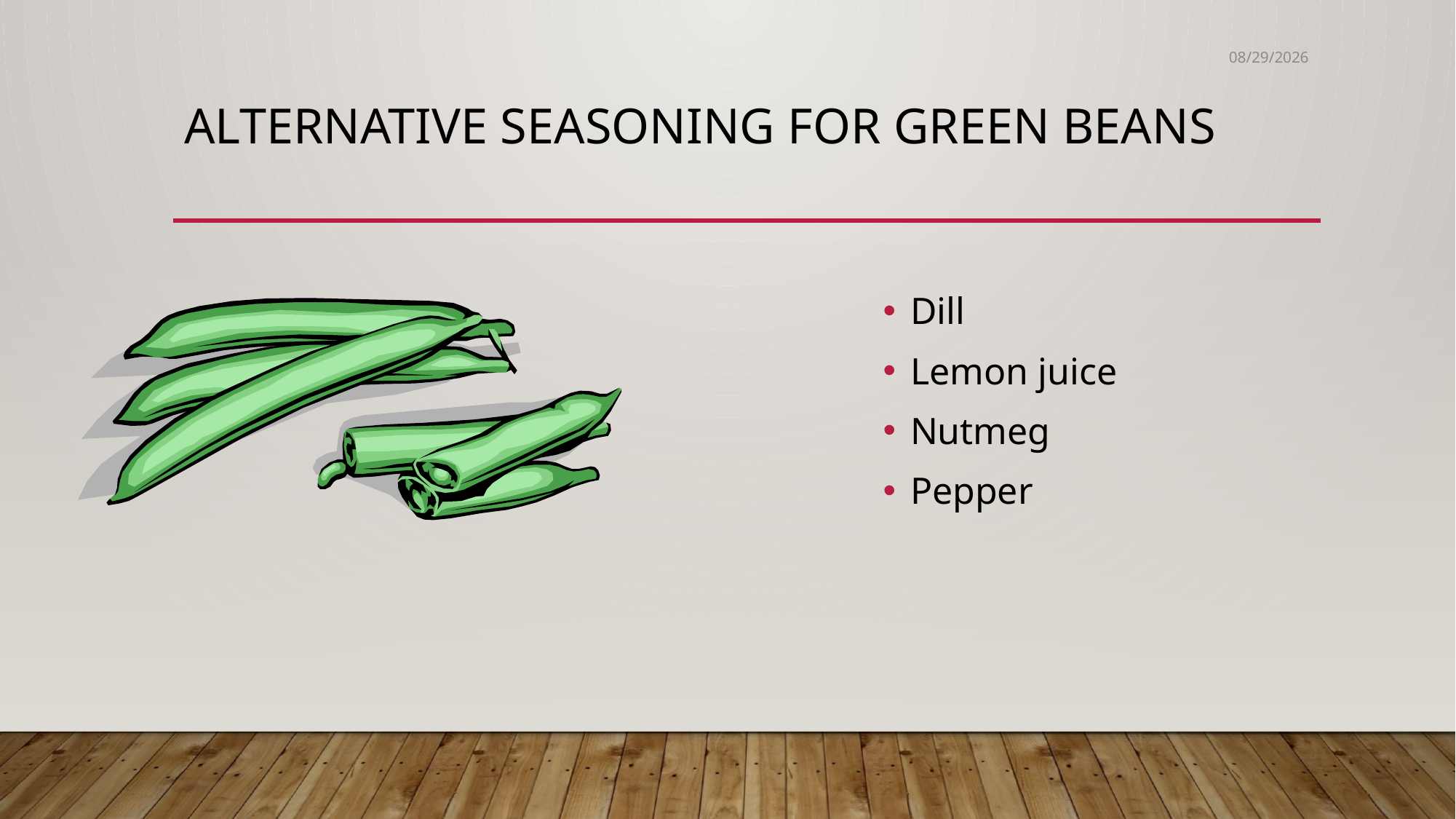

4/4/23
# Alternative seasoning for Green beans
Dill
Lemon juice
Nutmeg
Pepper

## Slide 20
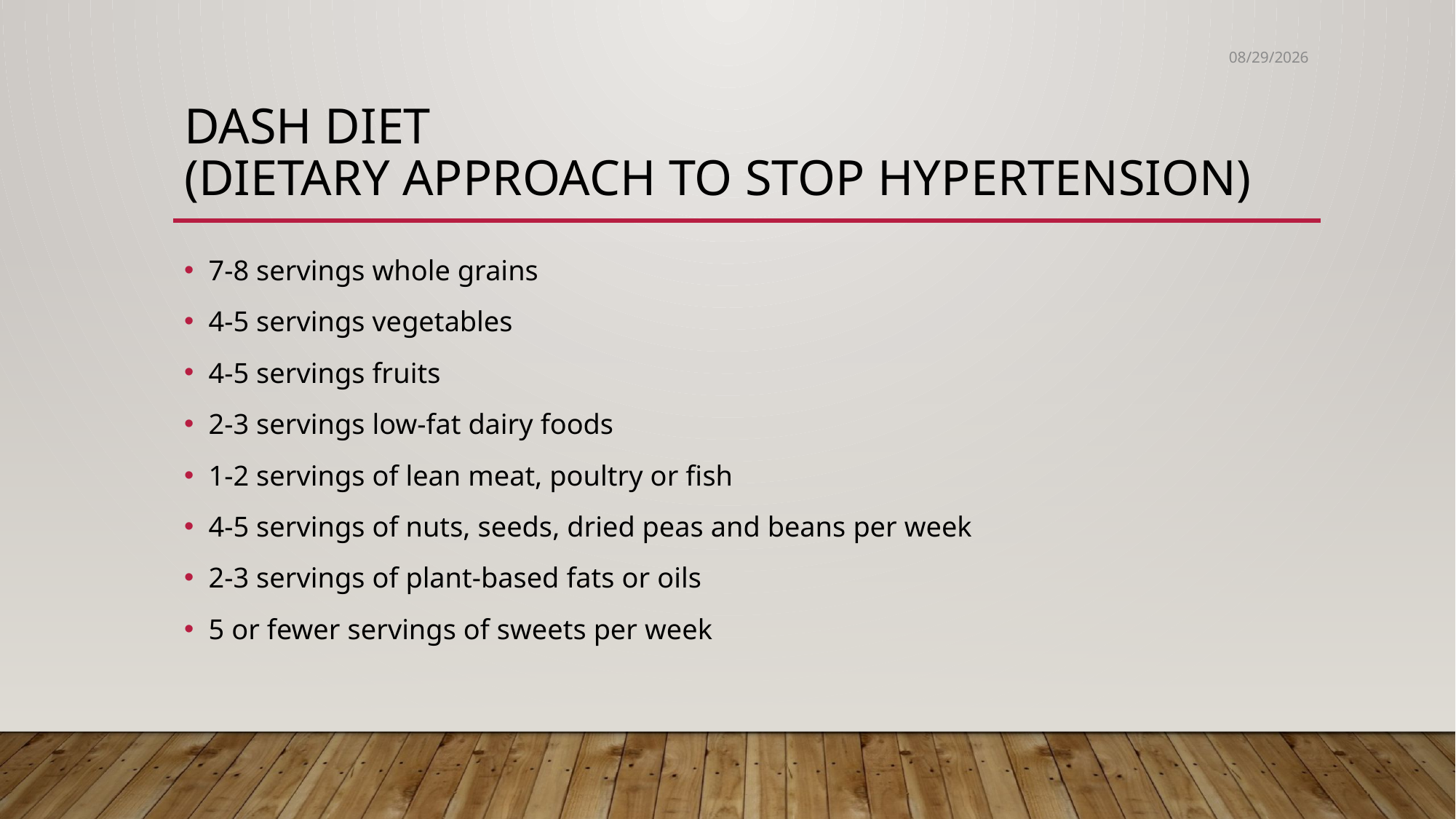

4/4/23
# DASH Diet (Dietary Approach to Stop Hypertension)
7-8 servings whole grains
4-5 servings vegetables
4-5 servings fruits
2-3 servings low-fat dairy foods
1-2 servings of lean meat, poultry or fish
4-5 servings of nuts, seeds, dried peas and beans per week
2-3 servings of plant-based fats or oils
5 or fewer servings of sweets per week

## Slide 21
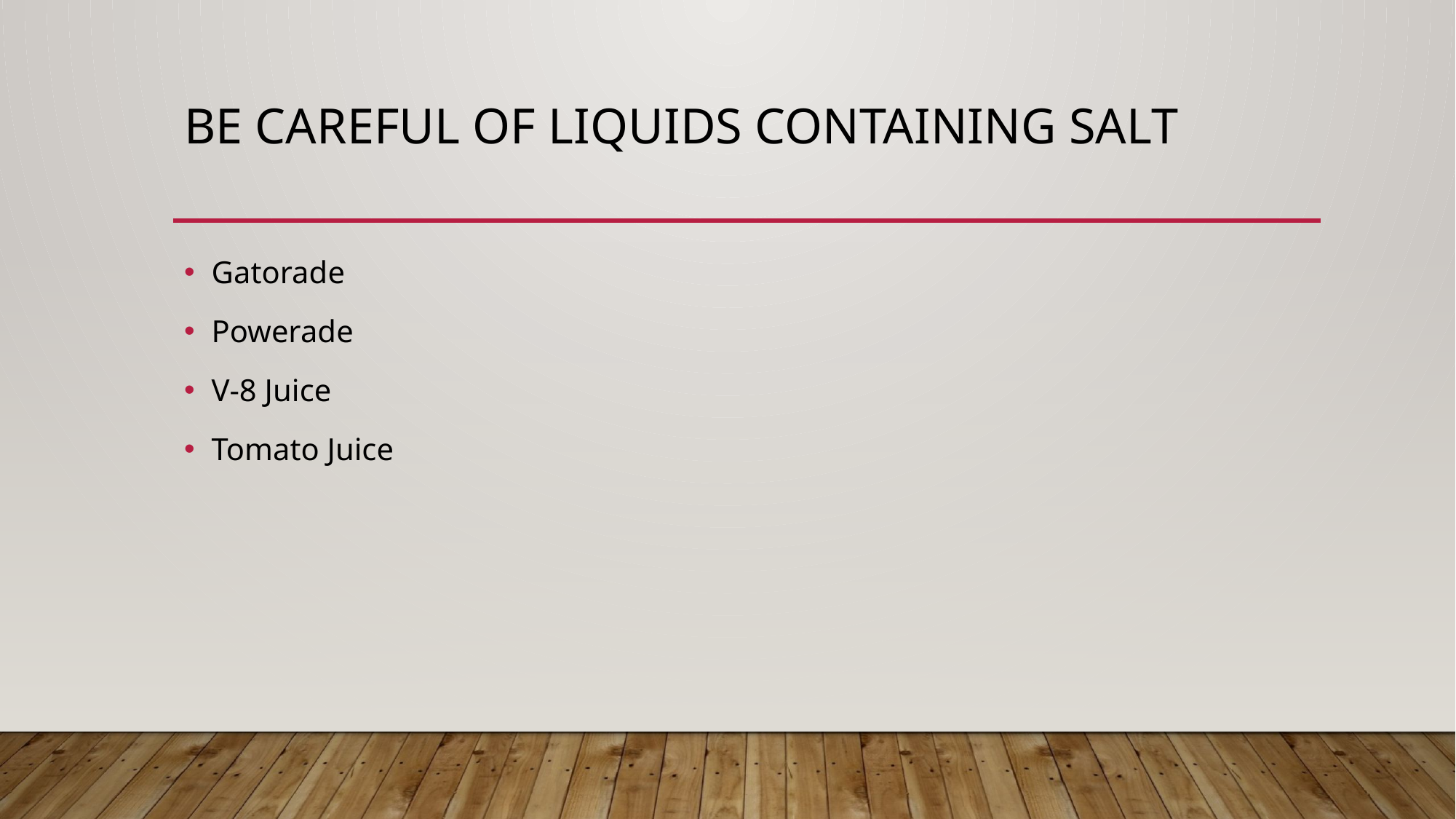

# Be careful of Liquids containing salt
Gatorade
Powerade
V-8 Juice
Tomato Juice

## Slide 22
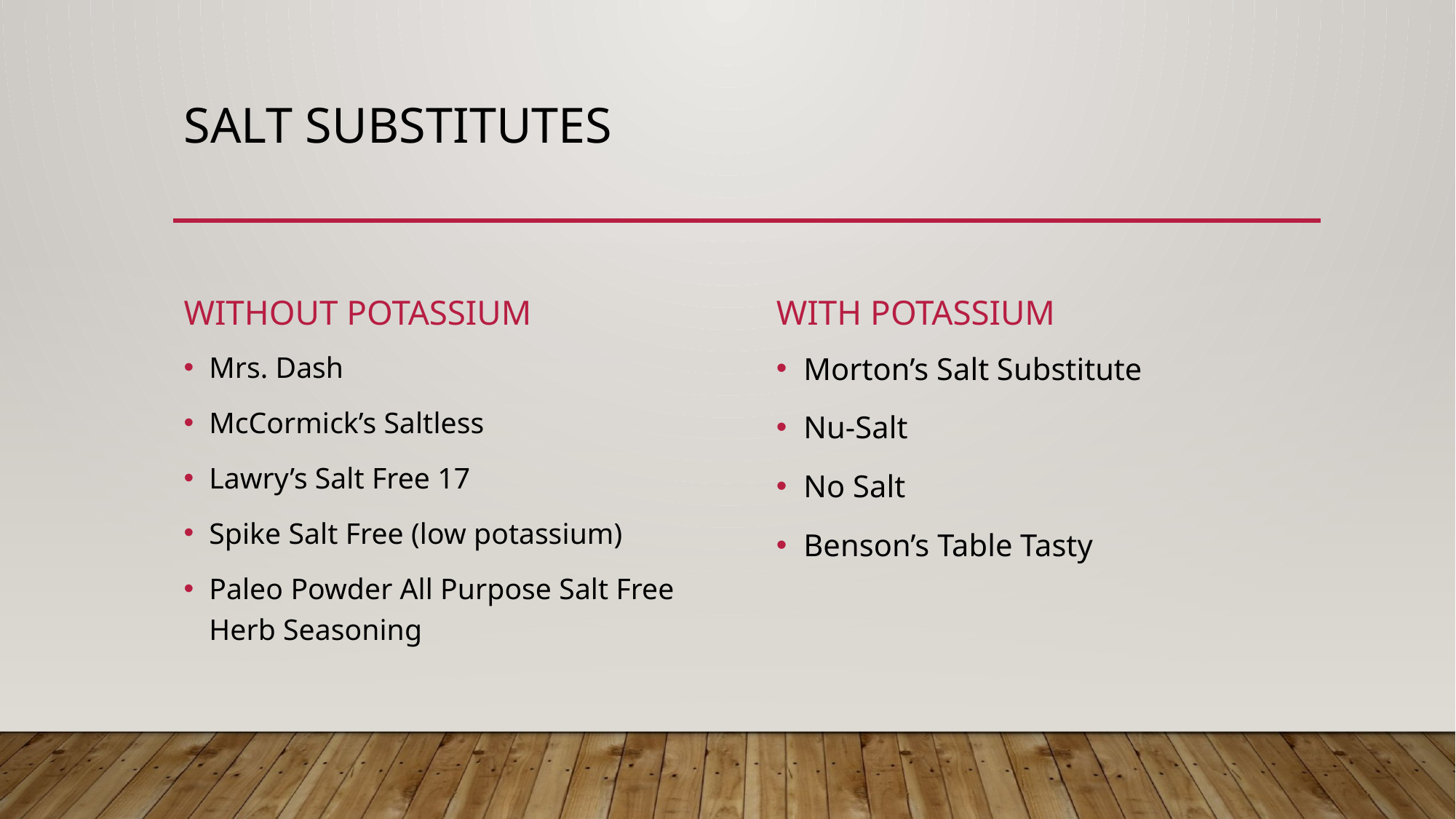

# Salt substitutes
Without potassium
With potassium
Morton’s Salt Substitute
Nu-Salt
No Salt
Benson’s Table Tasty
Mrs. Dash
McCormick’s Saltless
Lawry’s Salt Free 17
Spike Salt Free (low potassium)
Paleo Powder All Purpose Salt Free Herb Seasoning

## Slide 23
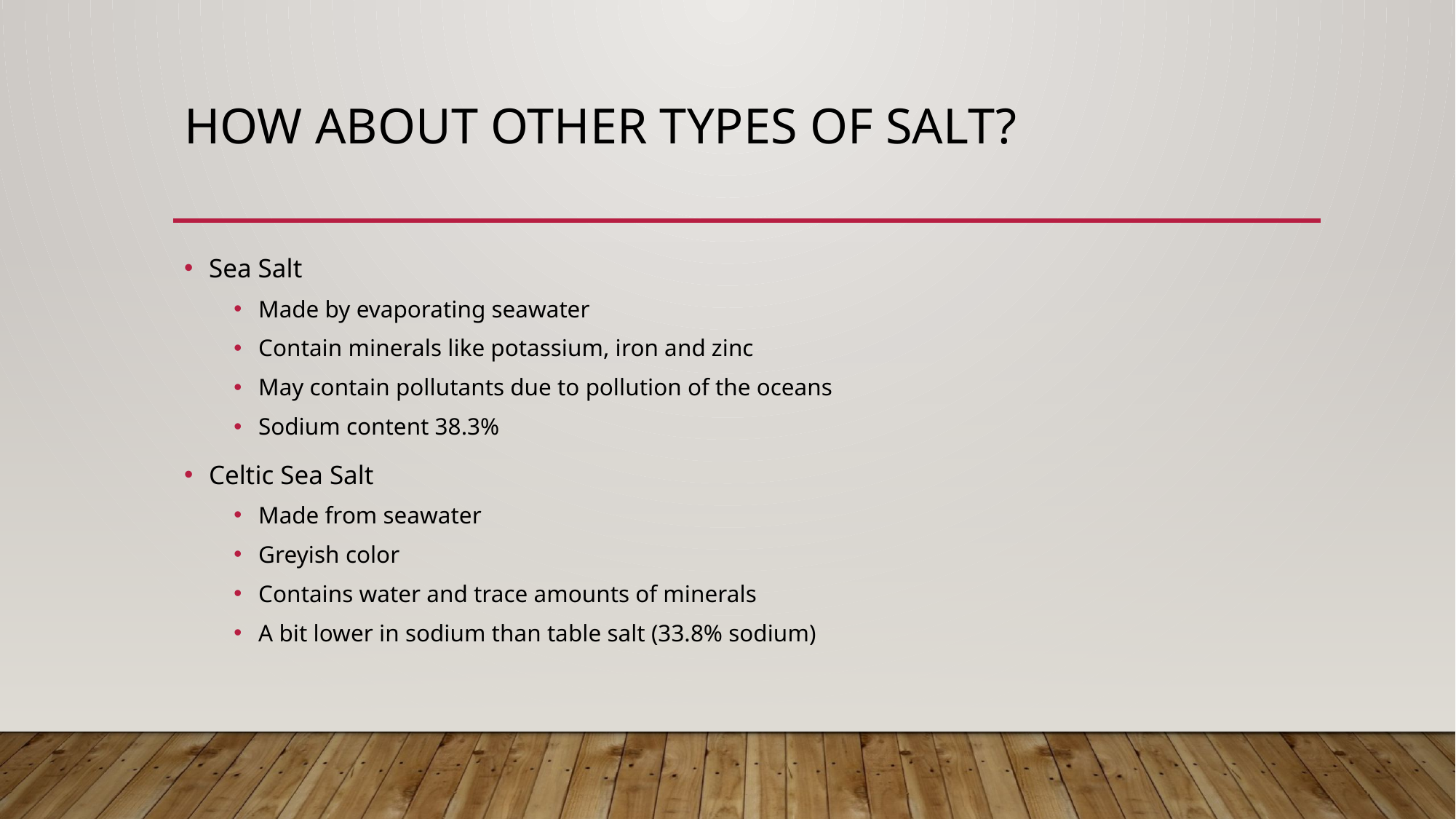

# How about Other types of salt?
Sea Salt
Made by evaporating seawater
Contain minerals like potassium, iron and zinc
May contain pollutants due to pollution of the oceans
Sodium content 38.3%
Celtic Sea Salt
Made from seawater
Greyish color
Contains water and trace amounts of minerals
A bit lower in sodium than table salt (33.8% sodium)

## Slide 24
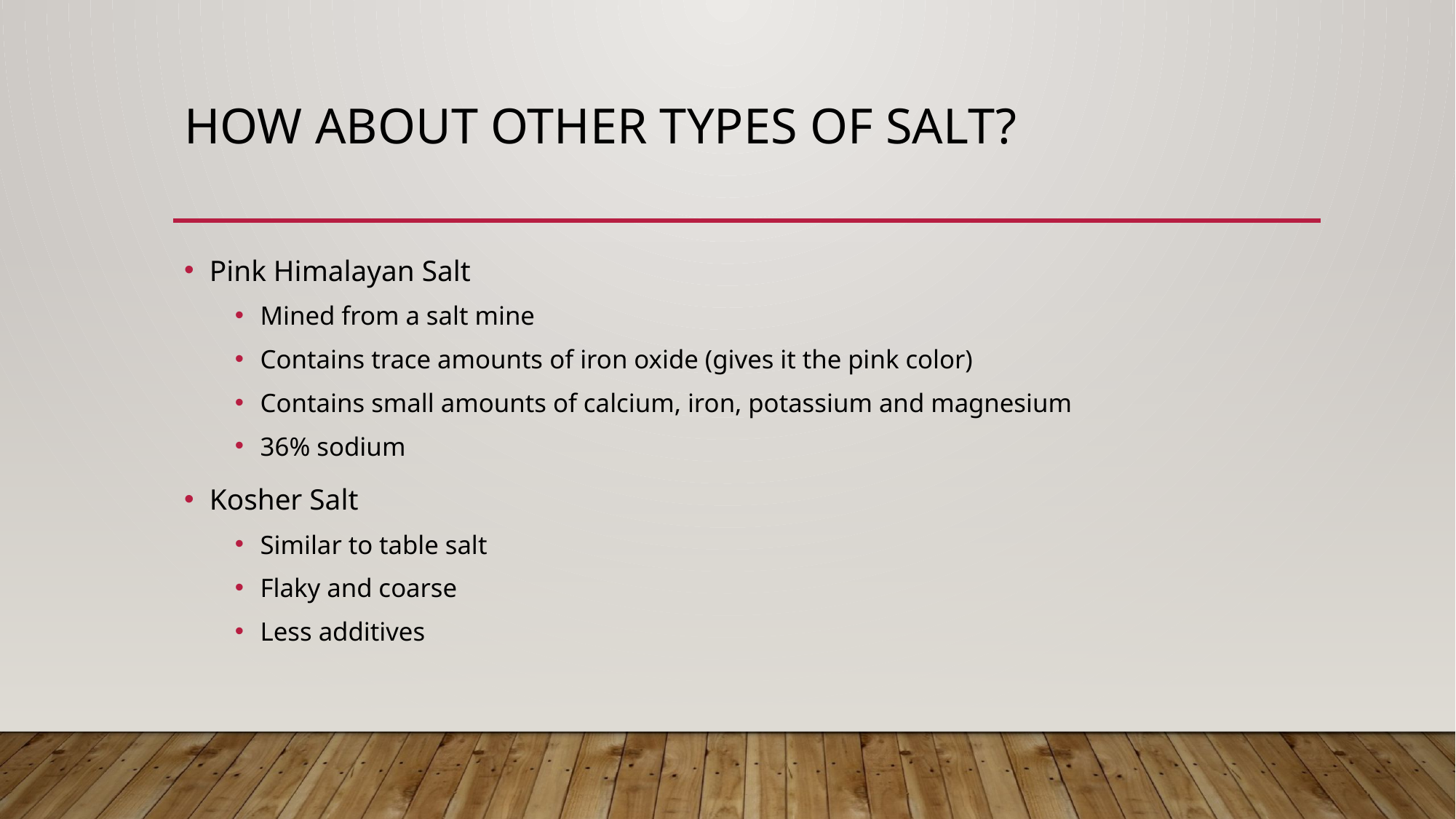

# How about Other types of salt?
Pink Himalayan Salt
Mined from a salt mine
Contains trace amounts of iron oxide (gives it the pink color)
Contains small amounts of calcium, iron, potassium and magnesium
36% sodium
Kosher Salt
Similar to table salt
Flaky and coarse
Less additives

## Slide 25
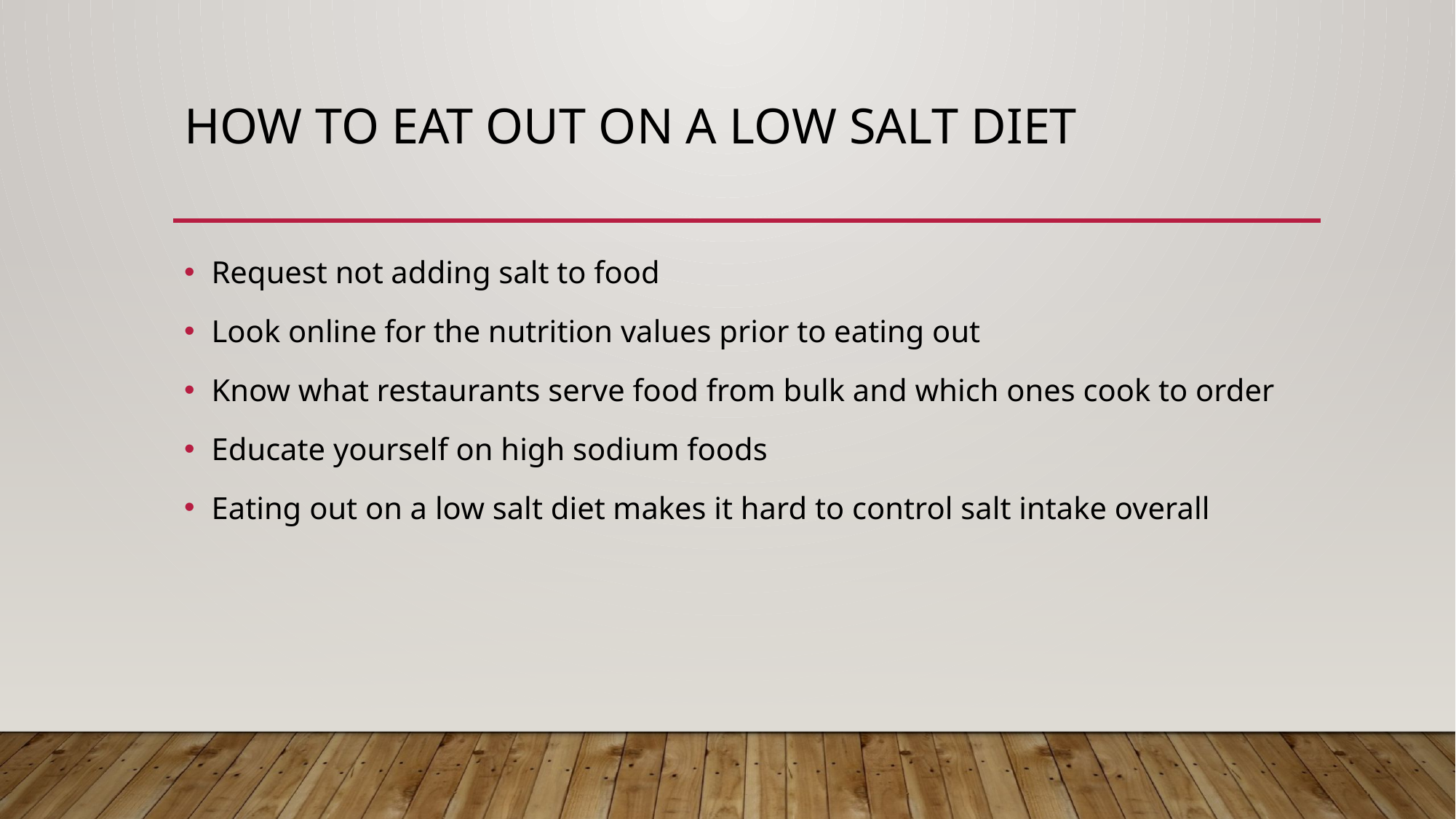

# How to eat out on a low salt diet
Request not adding salt to food
Look online for the nutrition values prior to eating out
Know what restaurants serve food from bulk and which ones cook to order
Educate yourself on high sodium foods
Eating out on a low salt diet makes it hard to control salt intake overall

## Slide 26
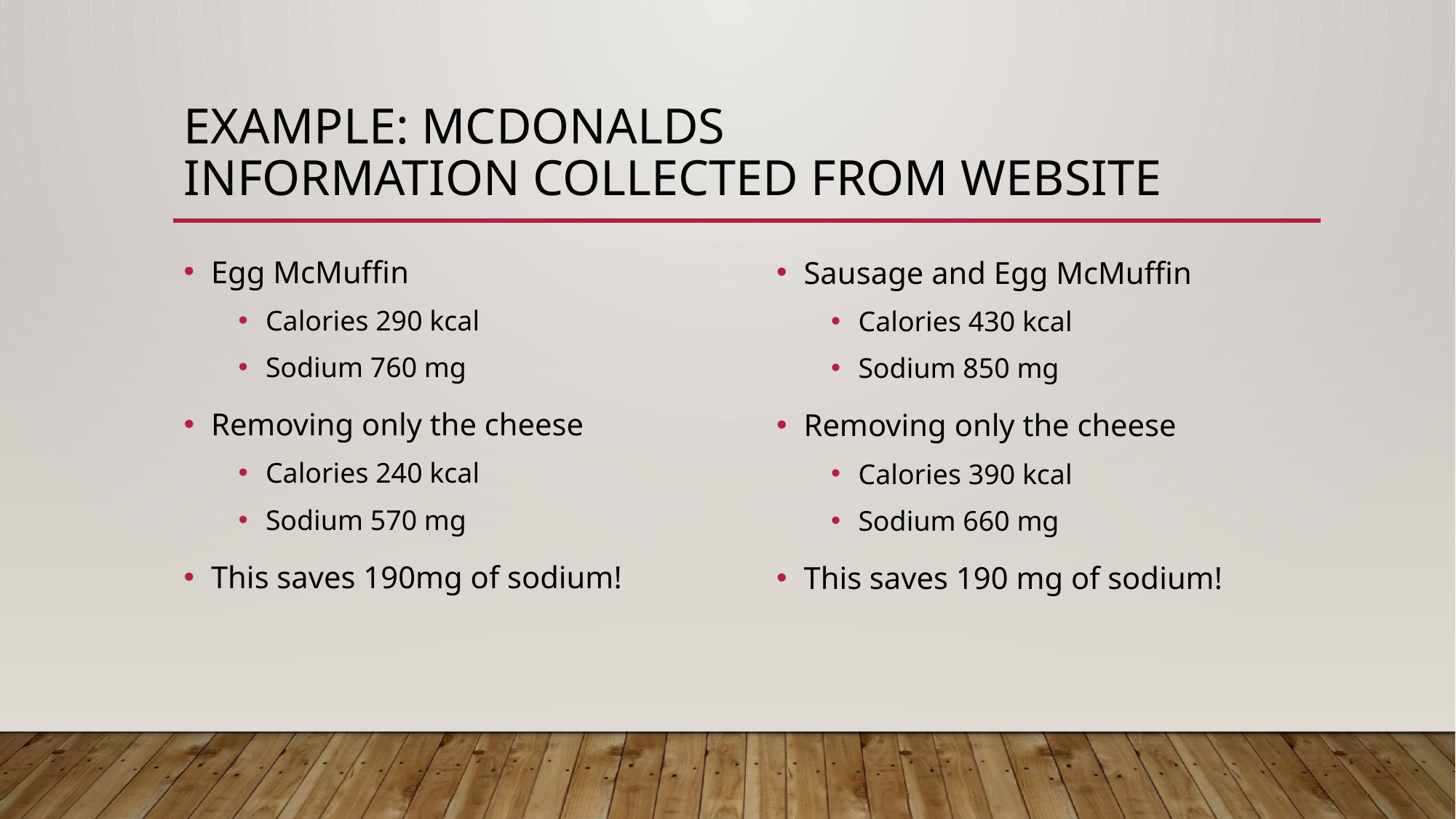

# Example: Mcdonalds information collected from website
Egg McMuffin
Calories 290 kcal
Sodium 760 mg
Removing only the cheese
Calories 240 kcal
Sodium 570 mg
This saves 190mg of sodium!
Sausage and Egg McMuffin
Calories 430 kcal
Sodium 850 mg
Removing only the cheese
Calories 390 kcal
Sodium 660 mg
This saves 190 mg of sodium!

## Slide 27
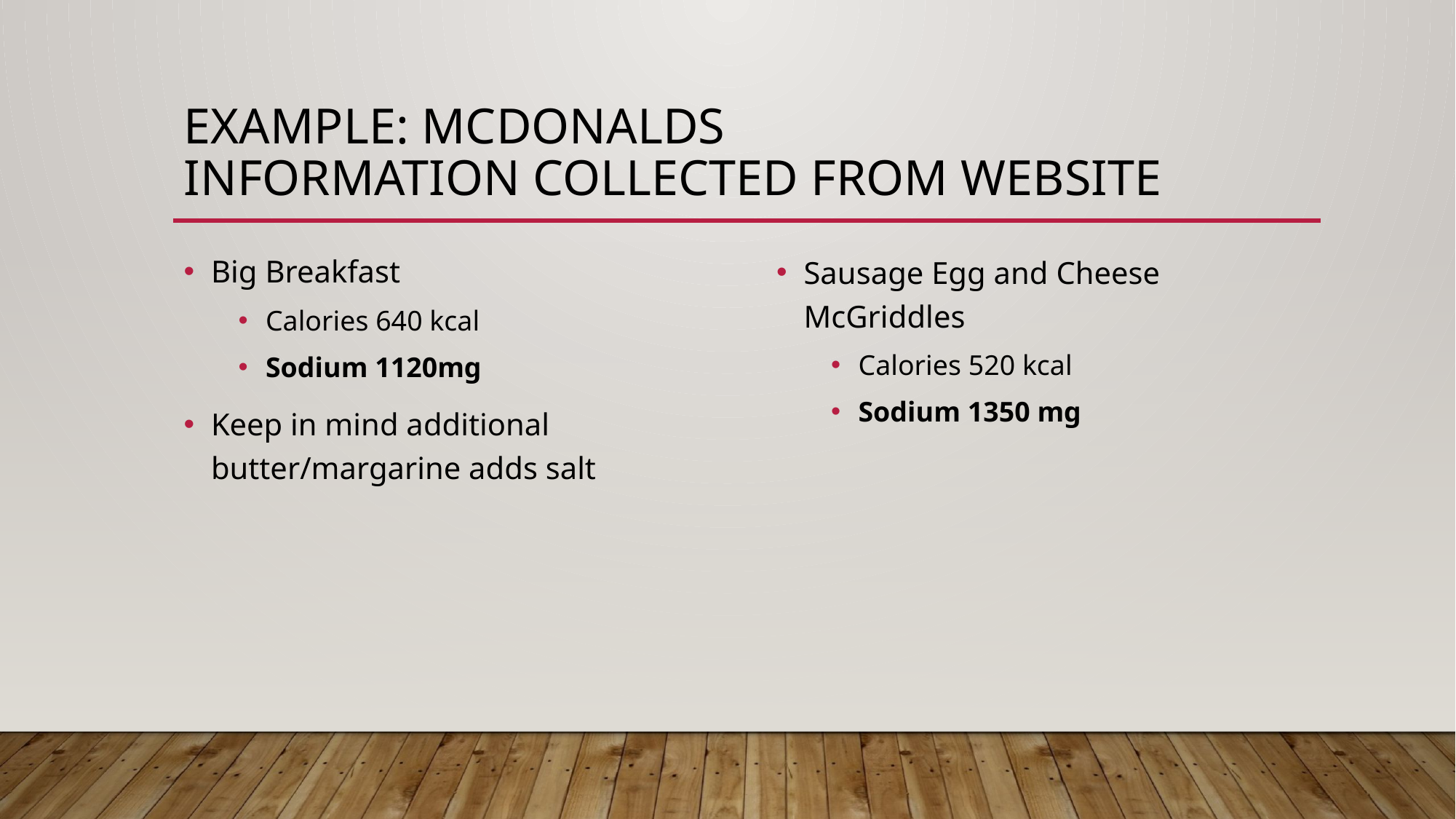

# Example: Mcdonalds information collected from website
Big Breakfast
Calories 640 kcal
Sodium 1120mg
Keep in mind additional butter/margarine adds salt
Sausage Egg and Cheese McGriddles
Calories 520 kcal
Sodium 1350 mg

## Slide 28
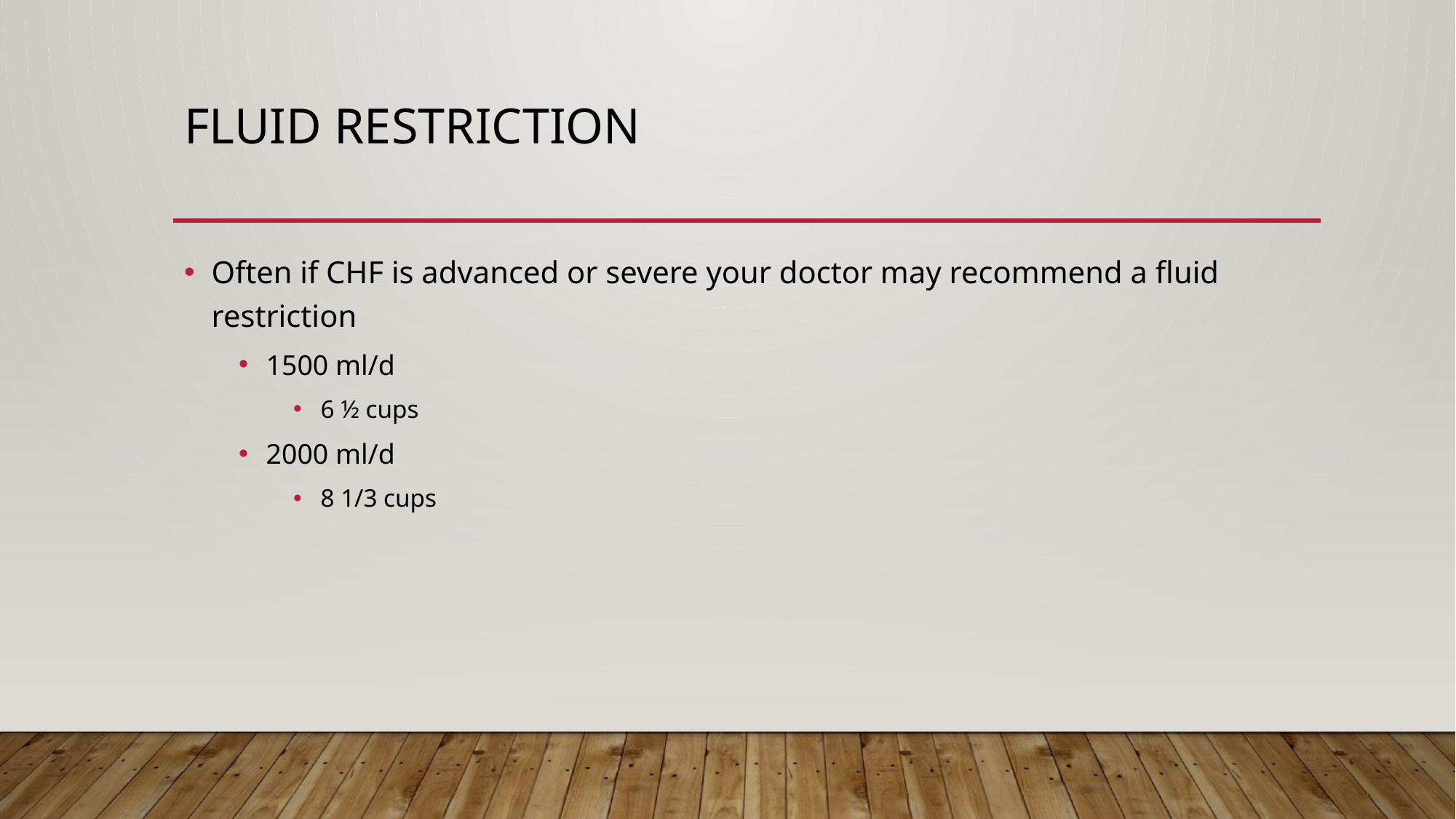

# Fluid restriction
Often if CHF is advanced or severe your doctor may recommend a fluid restriction
1500 ml/d
6 ½ cups
2000 ml/d
8 1/3 cups

## Slide 29
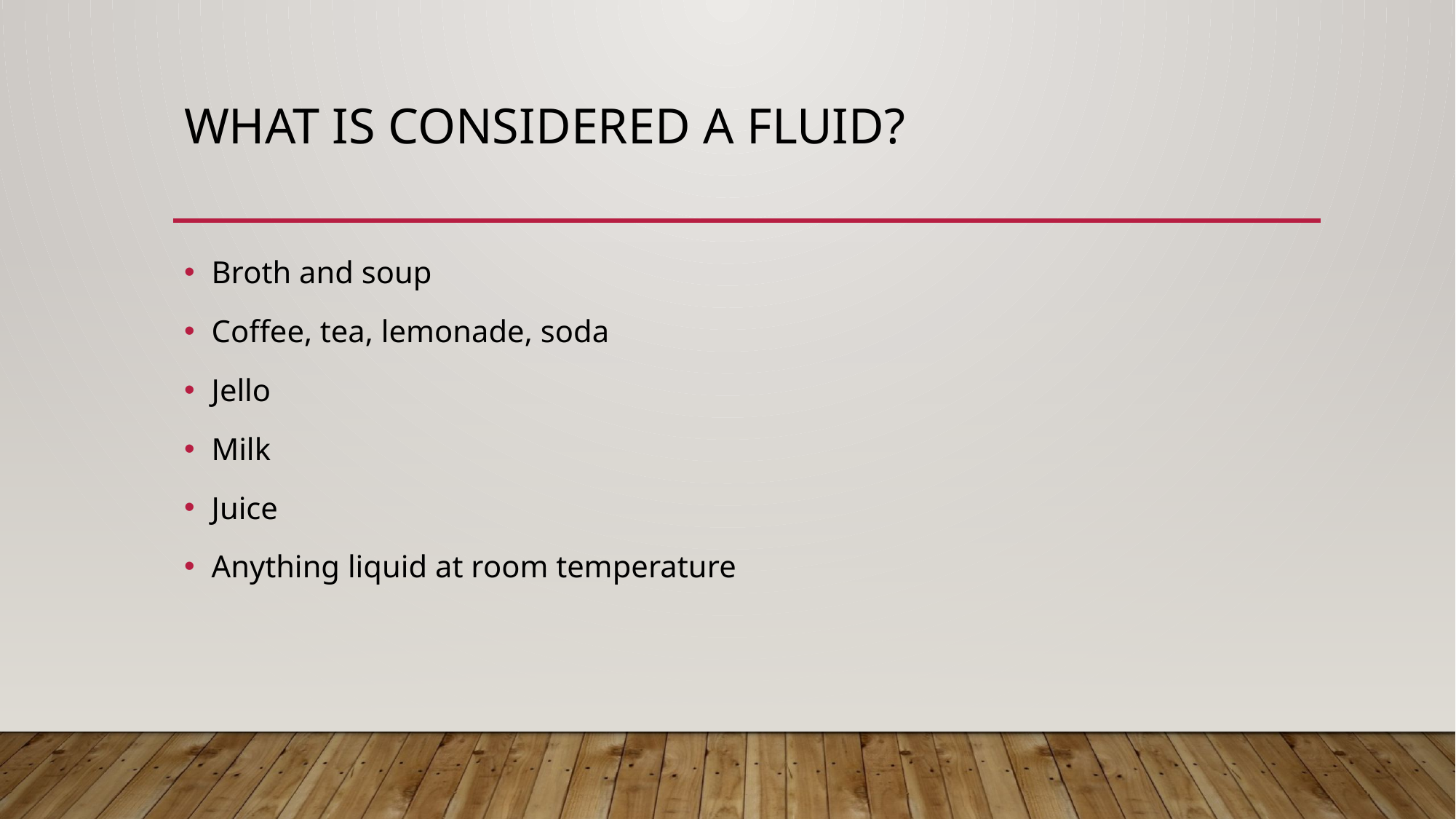

# What is considered a fluid?
Broth and soup
Coffee, tea, lemonade, soda
Jello
Milk
Juice
Anything liquid at room temperature

## Slide 30
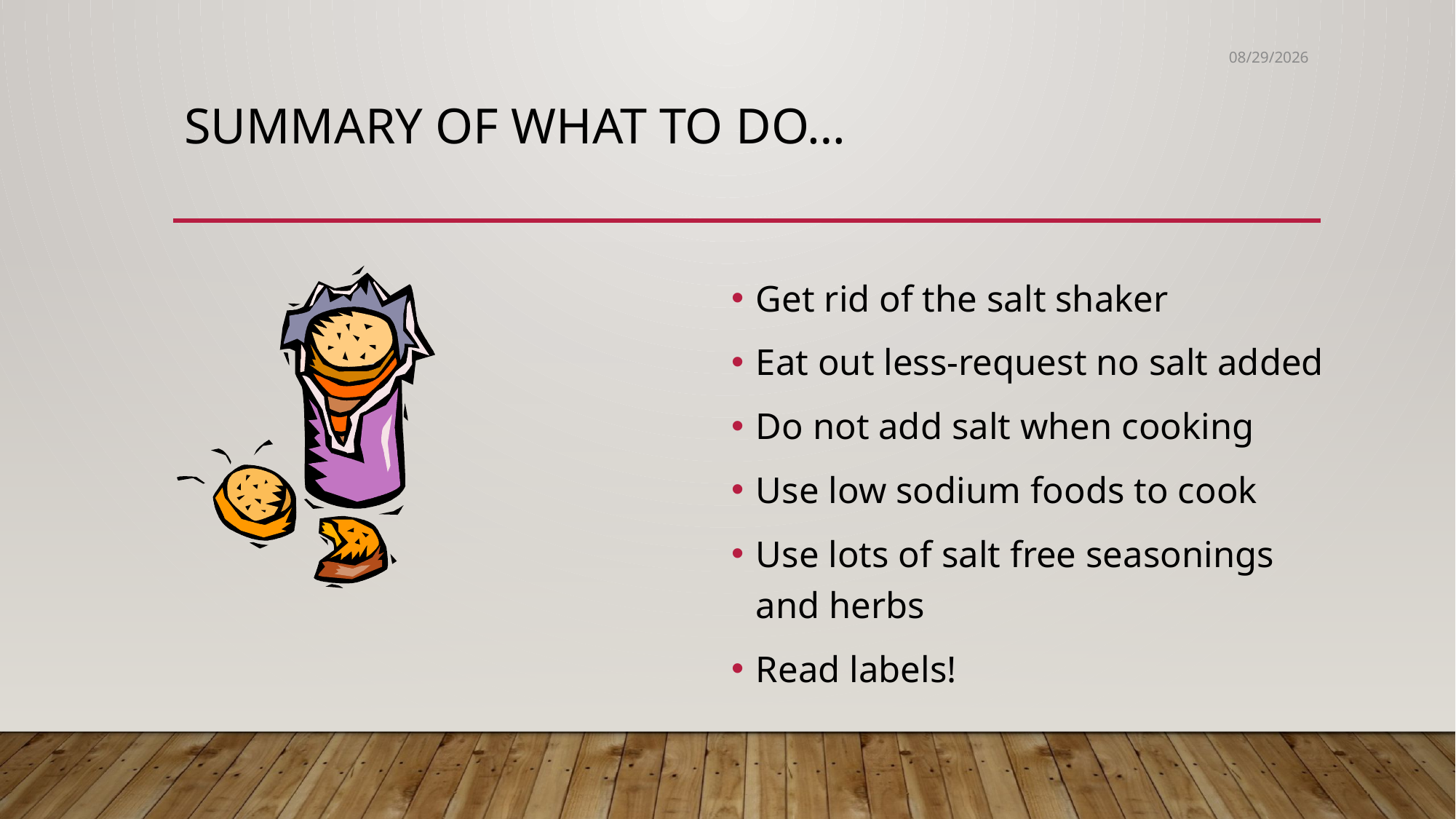

4/4/23
# Summary of What to do…
Get rid of the salt shaker
Eat out less-request no salt added
Do not add salt when cooking
Use low sodium foods to cook
Use lots of salt free seasonings and herbs
Read labels!

## Slide 31
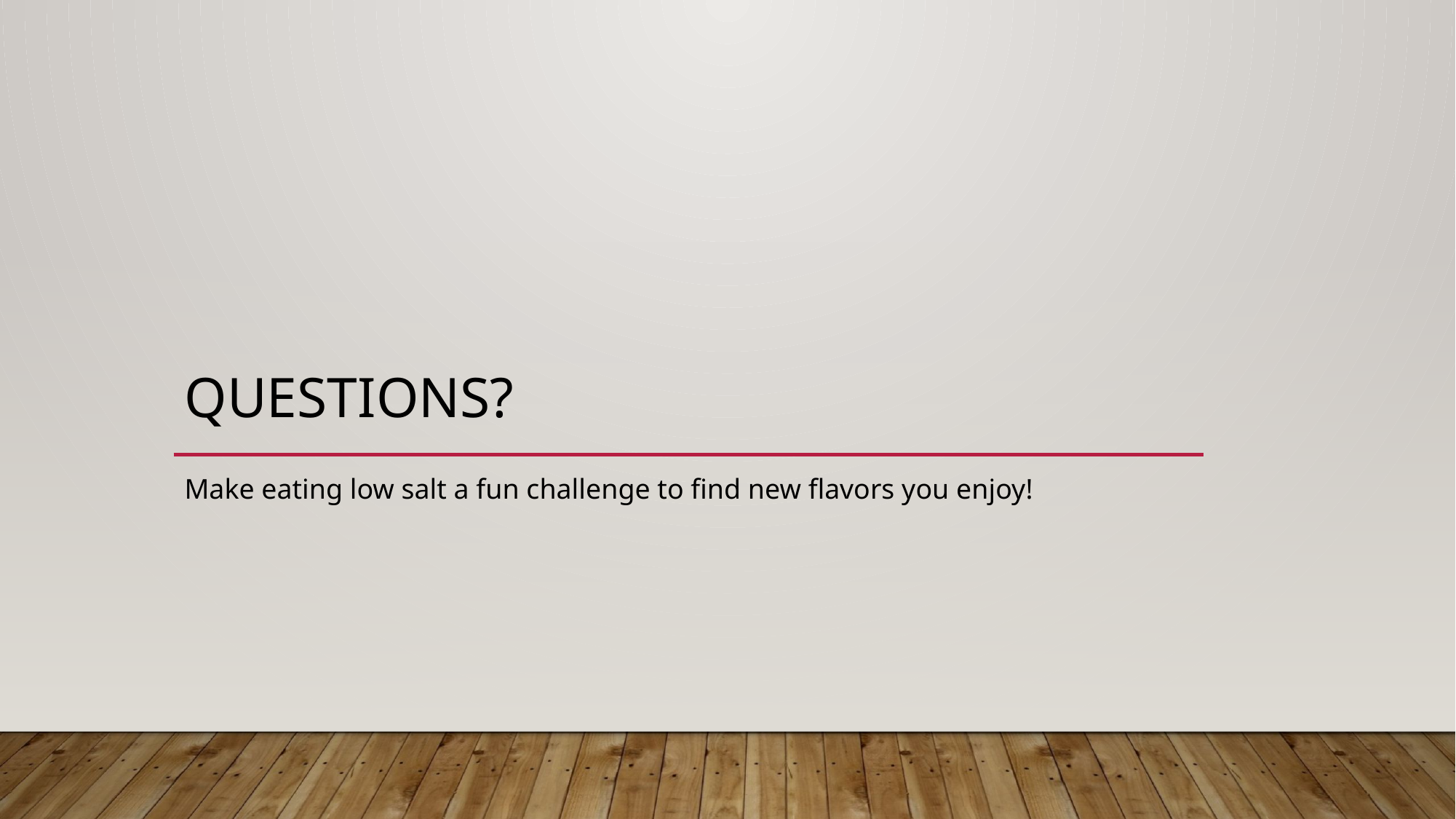

# Questions?
Make eating low salt a fun challenge to find new flavors you enjoy!
