# Supplementary material for: Implementation of a heart failure educational intervention for patients with recent admissions for acute decompensated heart failure
Source: Front Cardiovasc Med. 2023 May 5;10:1133988. doi: 10.3389/fcvm.2023.1133988 (PMC10196446; doi:10.3389/fcvm.2023.1133988)
Supplement: Supplementary file 2 [file Presentation2.pptx]

## Slide 1
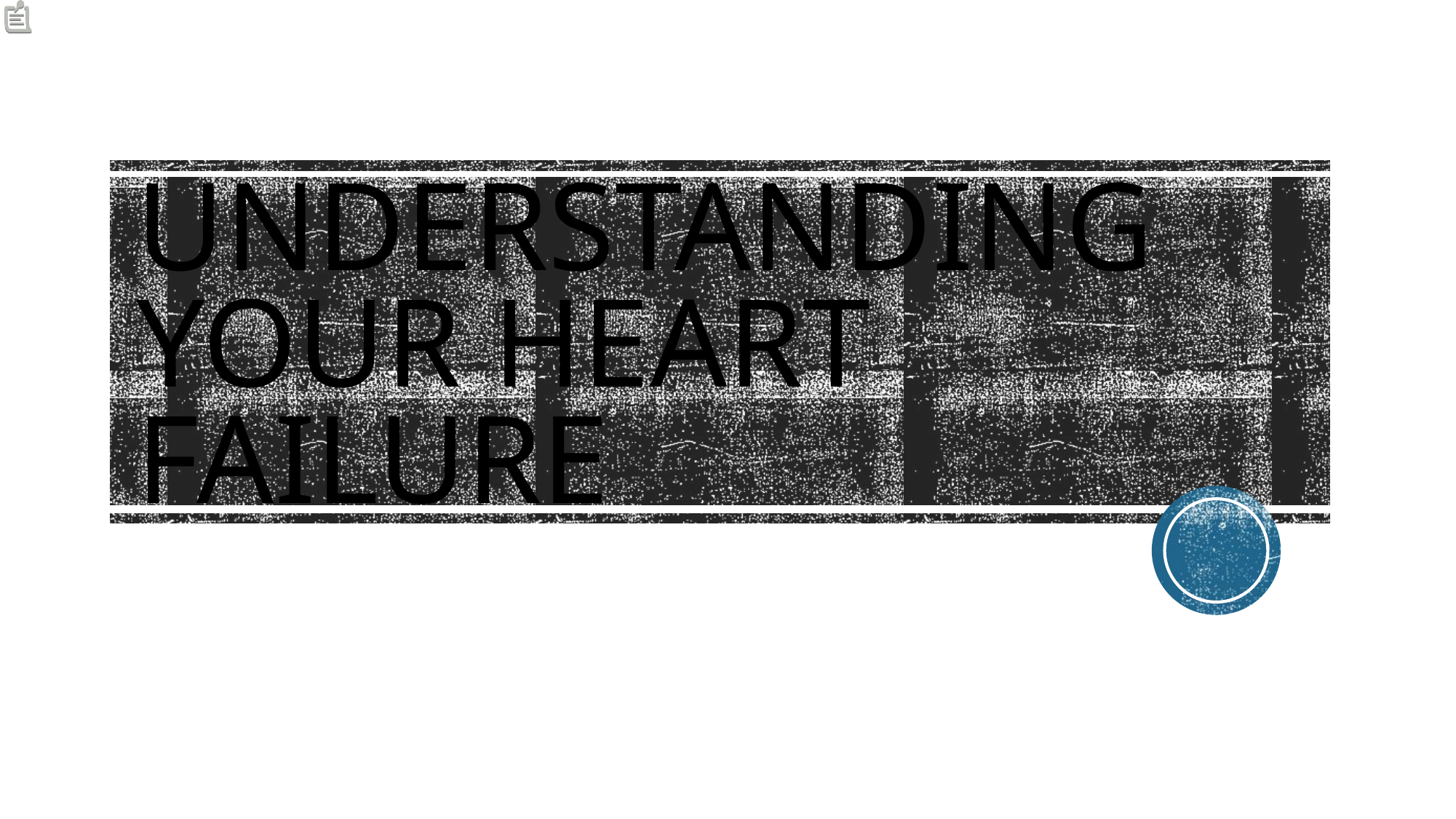

# Understanding your Heart Failure

## Slide 2
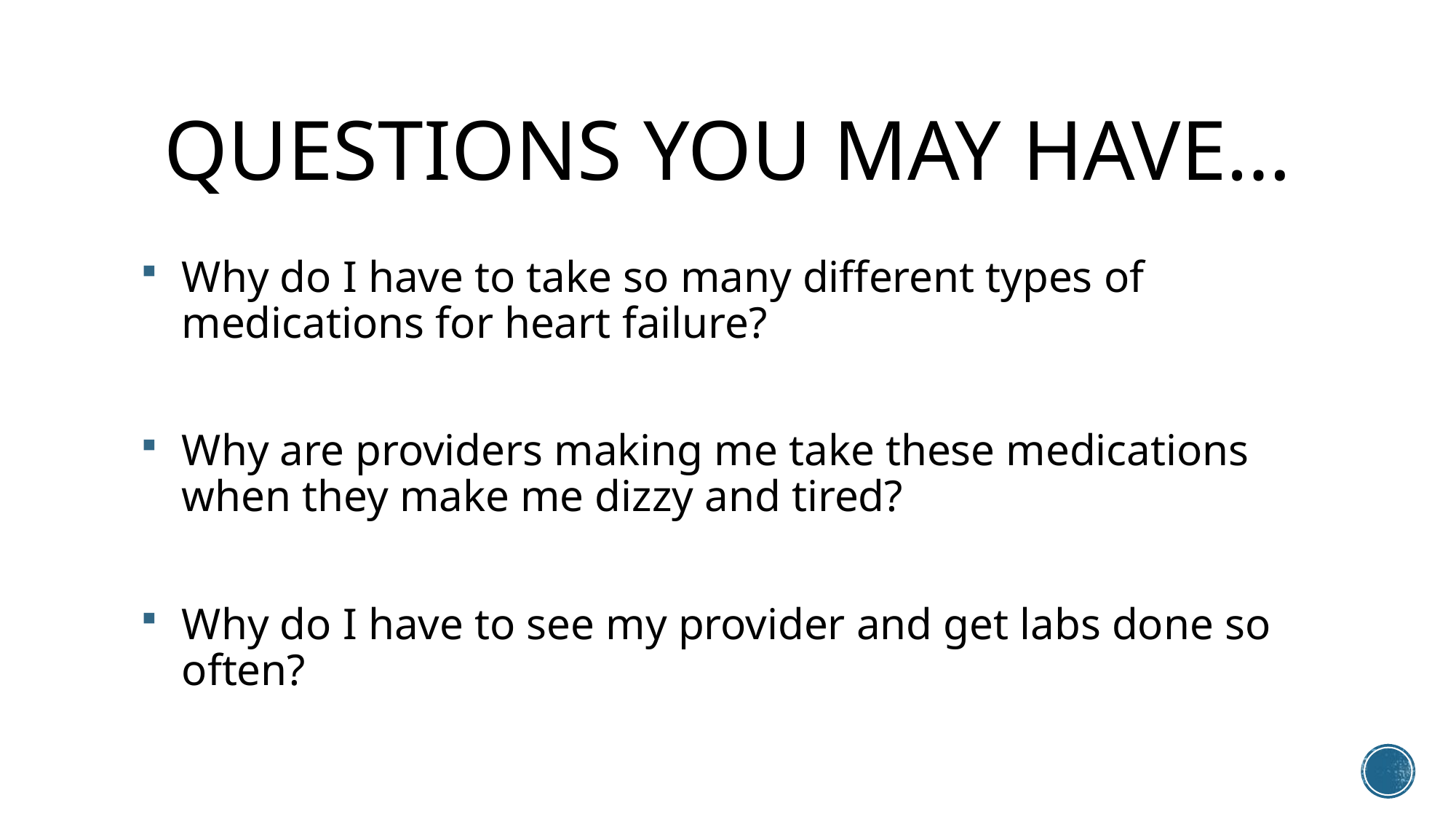

# Questions you may have…
Why do I have to take so many different types of medications for heart failure?
Why are providers making me take these medications when they make me dizzy and tired?
Why do I have to see my provider and get labs done so often?

## Slide 3
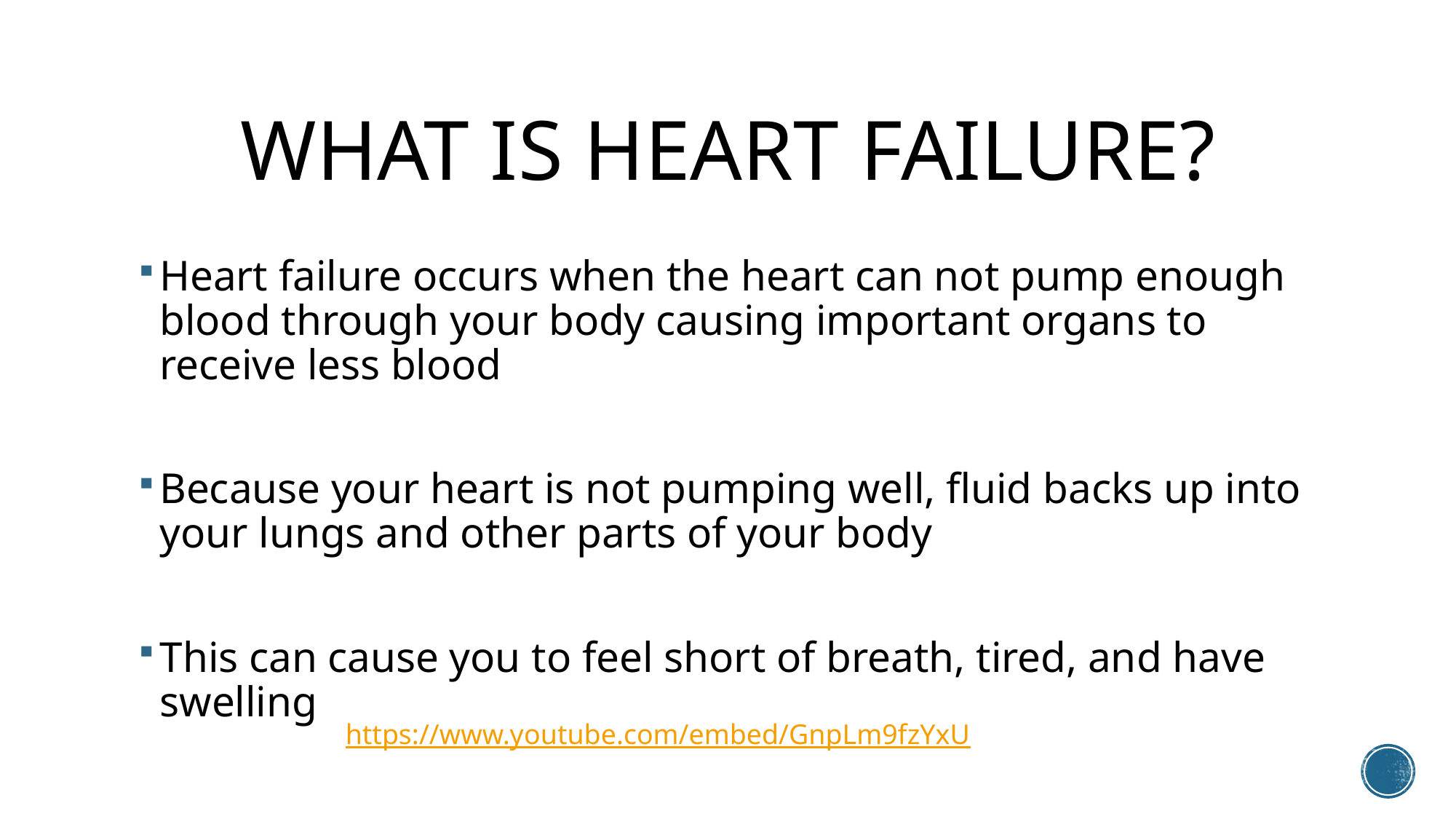

# What is Heart Failure?
Heart failure occurs when the heart can not pump enough blood through your body causing important organs to receive less blood
Because your heart is not pumping well, fluid backs up into your lungs and other parts of your body
This can cause you to feel short of breath, tired, and have swelling
https://www.youtube.com/embed/GnpLm9fzYxU

## Slide 4
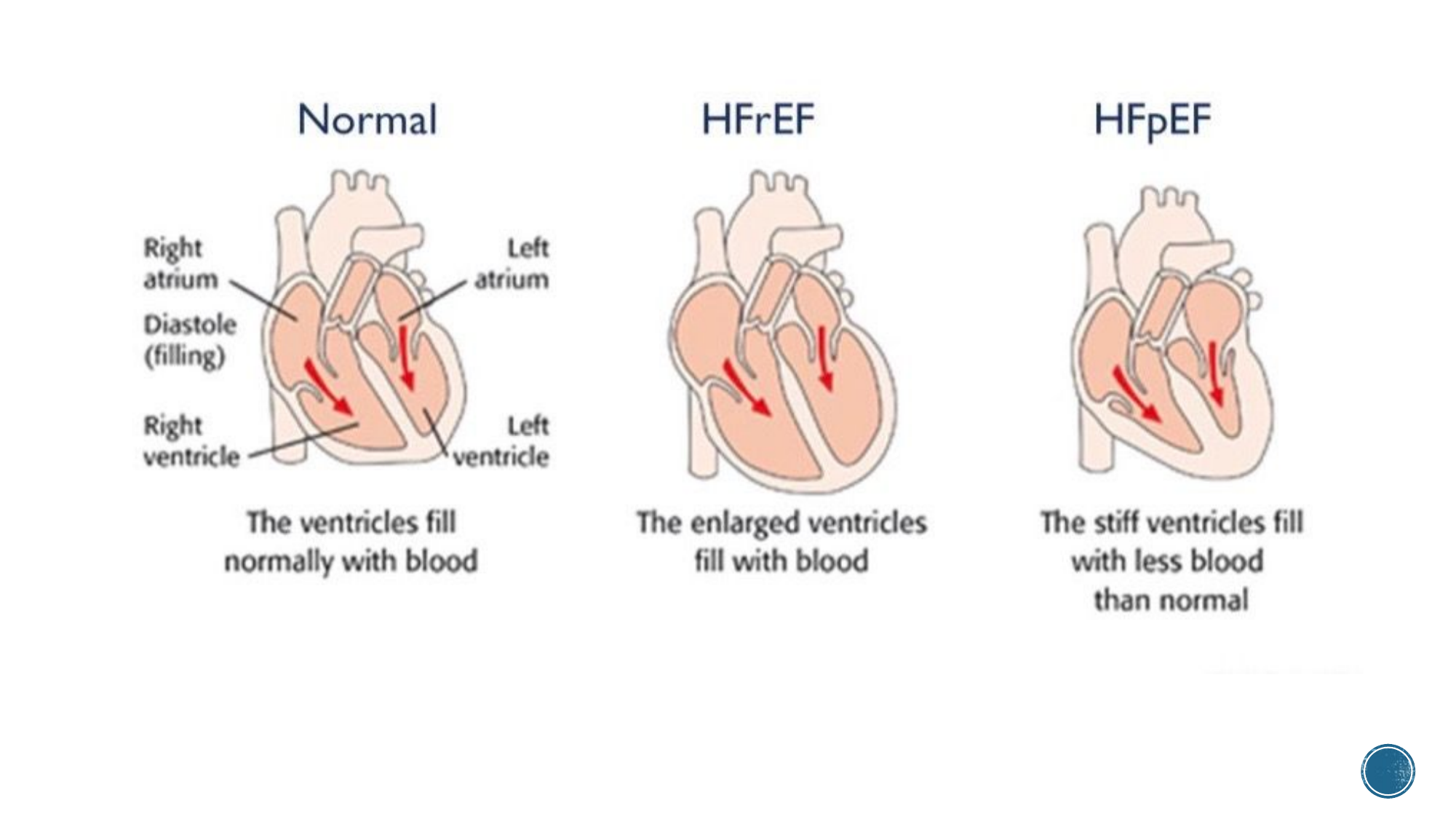

#

## Slide 5
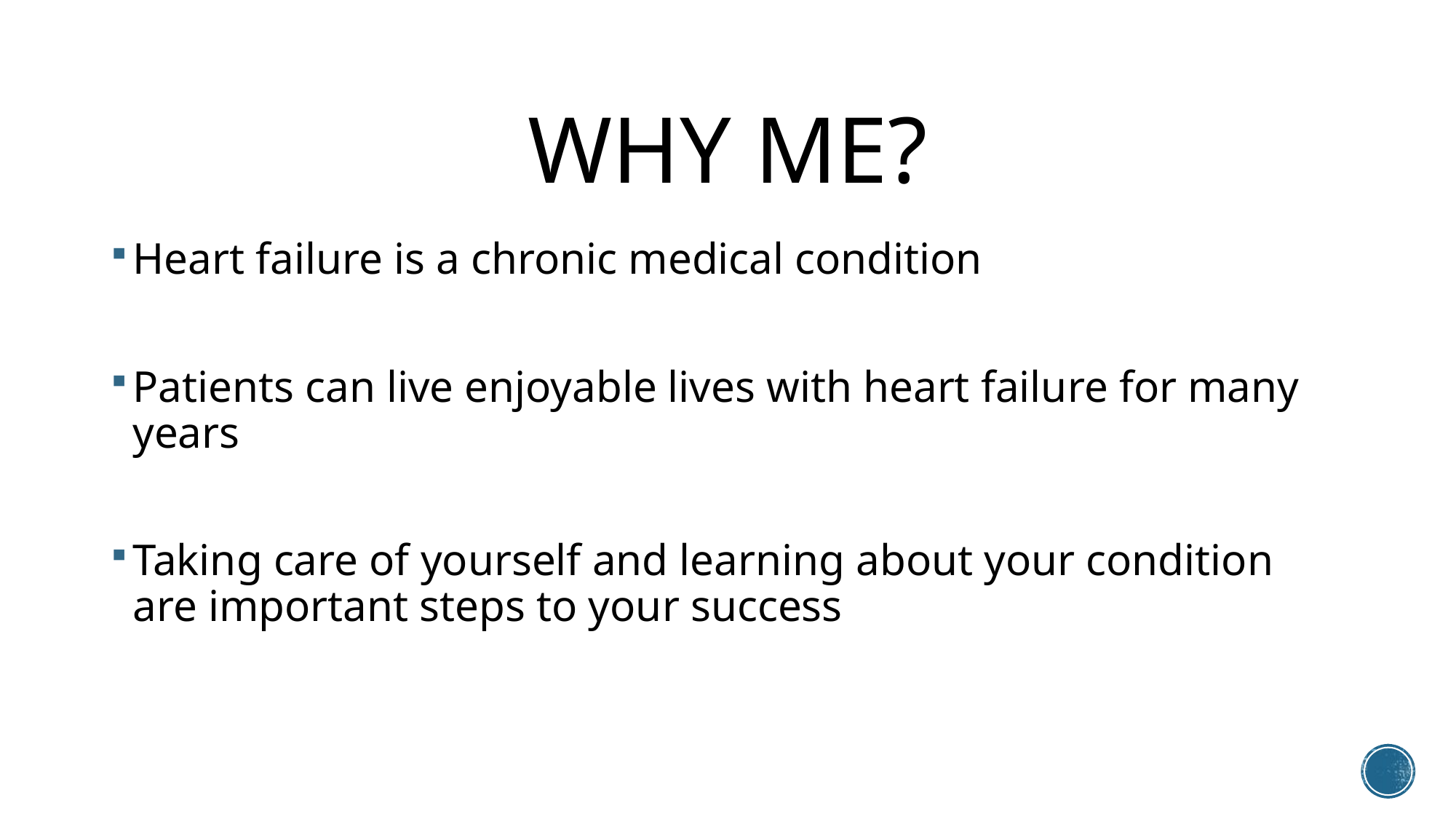

# Why me?
Heart failure is a chronic medical condition
Patients can live enjoyable lives with heart failure for many years
Taking care of yourself and learning about your condition are important steps to your success

## Slide 6
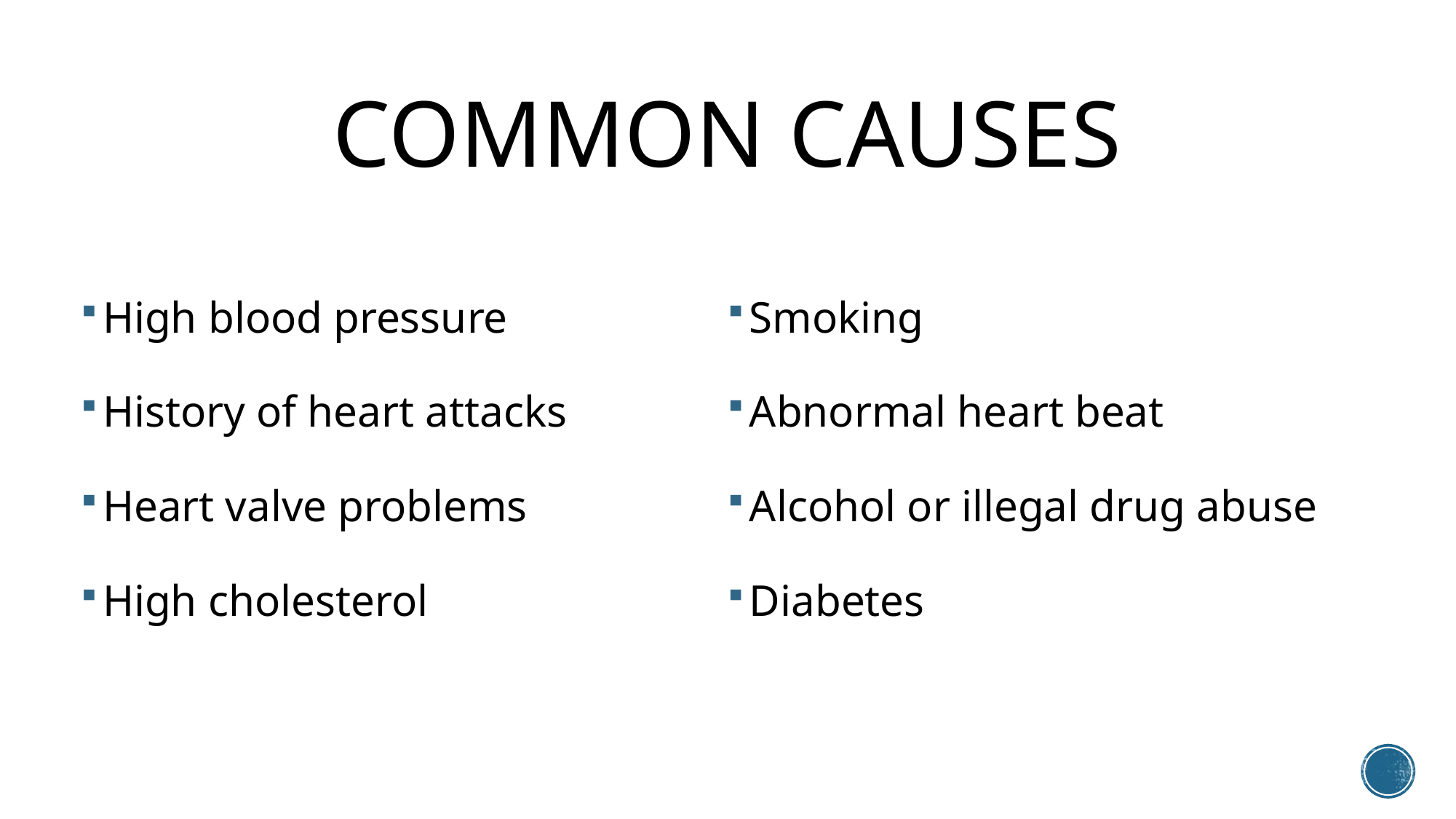

# Common Causes
High blood pressure
History of heart attacks
Heart valve problems
High cholesterol
Smoking
Abnormal heart beat
Alcohol or illegal drug abuse
Diabetes

## Slide 7
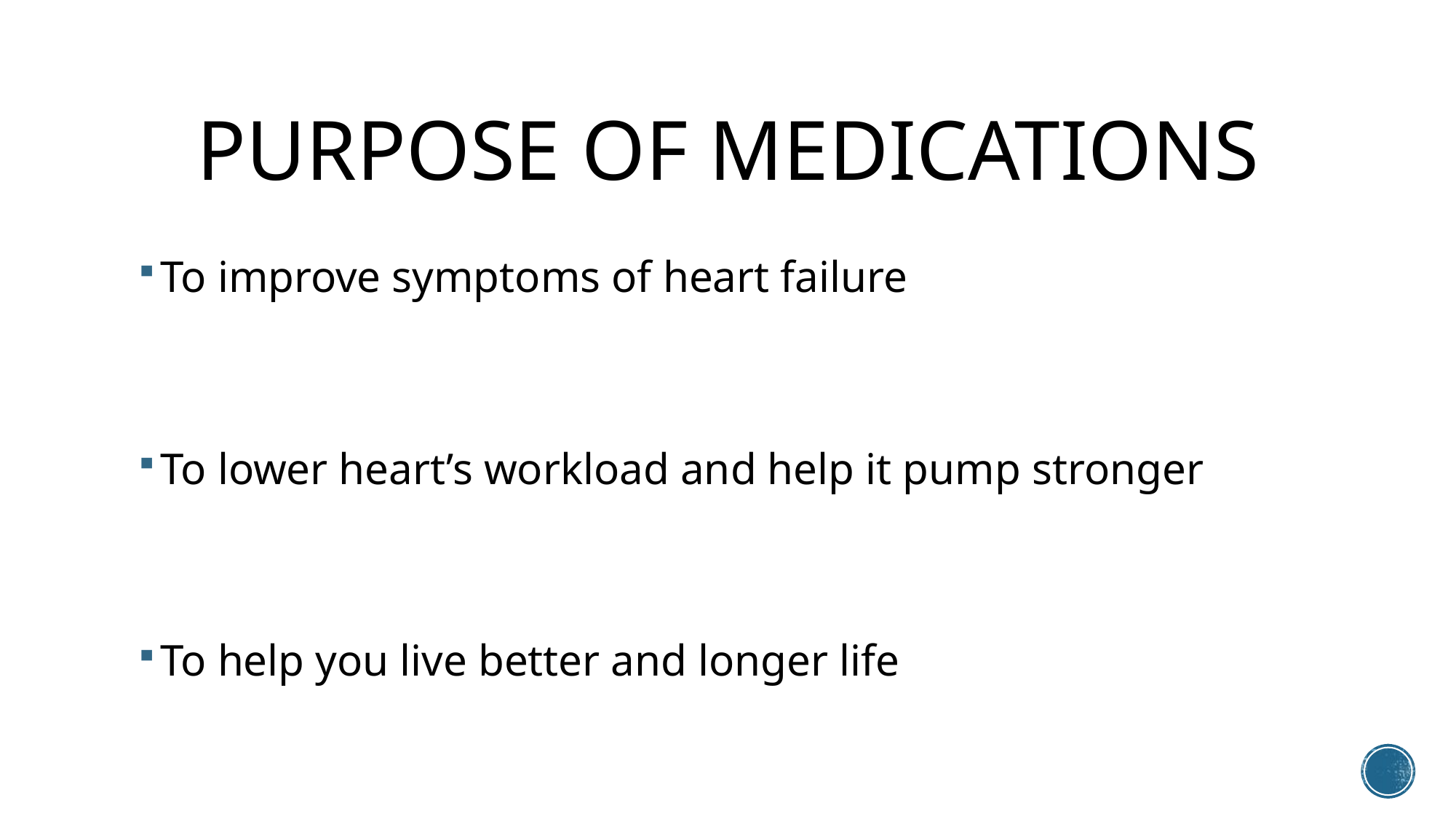

# Purpose of Medications
To improve symptoms of heart failure
To lower heart’s workload and help it pump stronger
To help you live better and longer life

## Slide 8
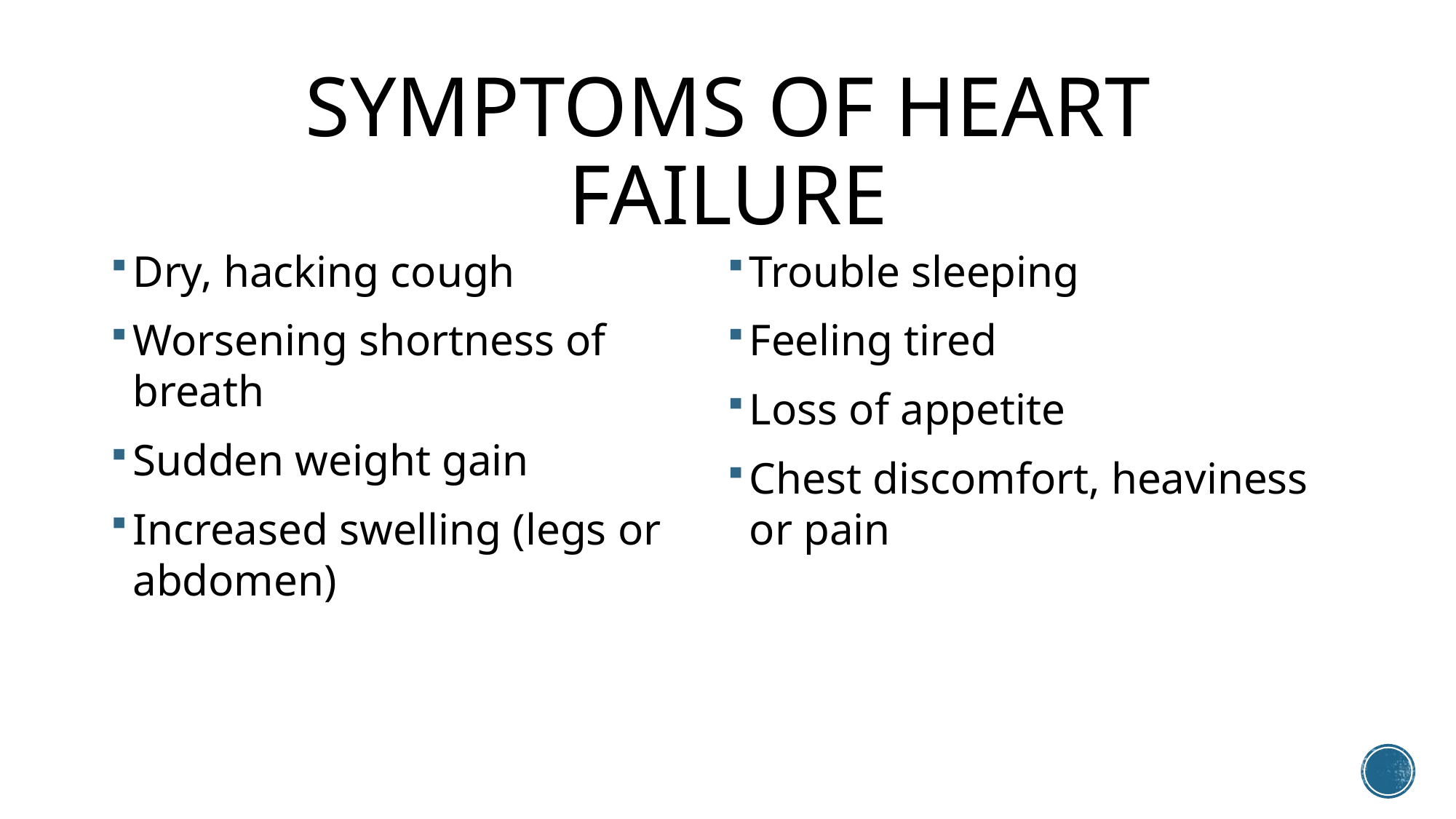

# Symptoms of heart failure
Dry, hacking cough
Worsening shortness of breath
Sudden weight gain
Increased swelling (legs or abdomen)
Trouble sleeping
Feeling tired
Loss of appetite
Chest discomfort, heaviness or pain

## Slide 9
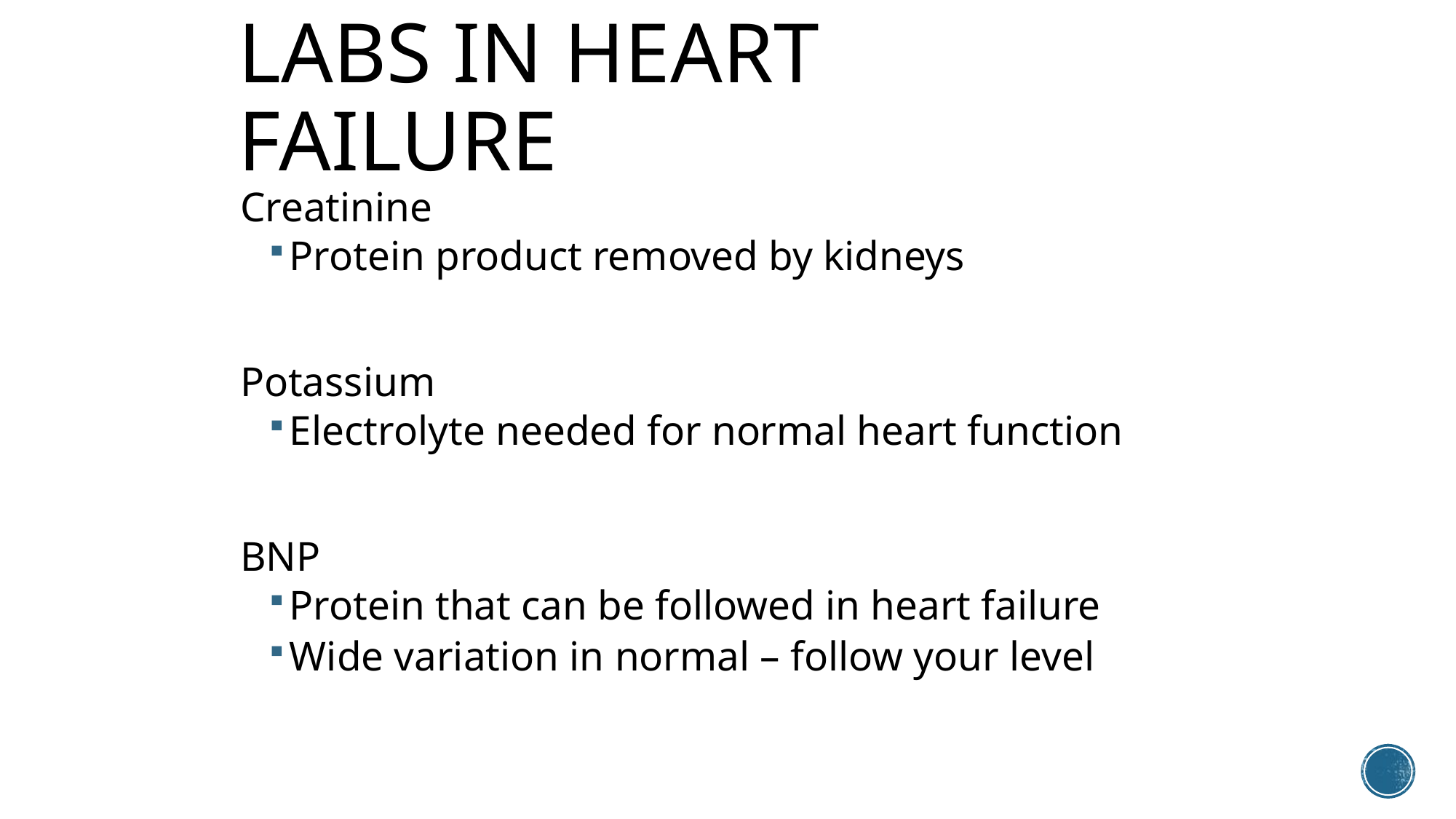

# Labs in Heart Failure
Creatinine
Protein product removed by kidneys
Potassium
Electrolyte needed for normal heart function
BNP
Protein that can be followed in heart failure
Wide variation in normal – follow your level

## Slide 10
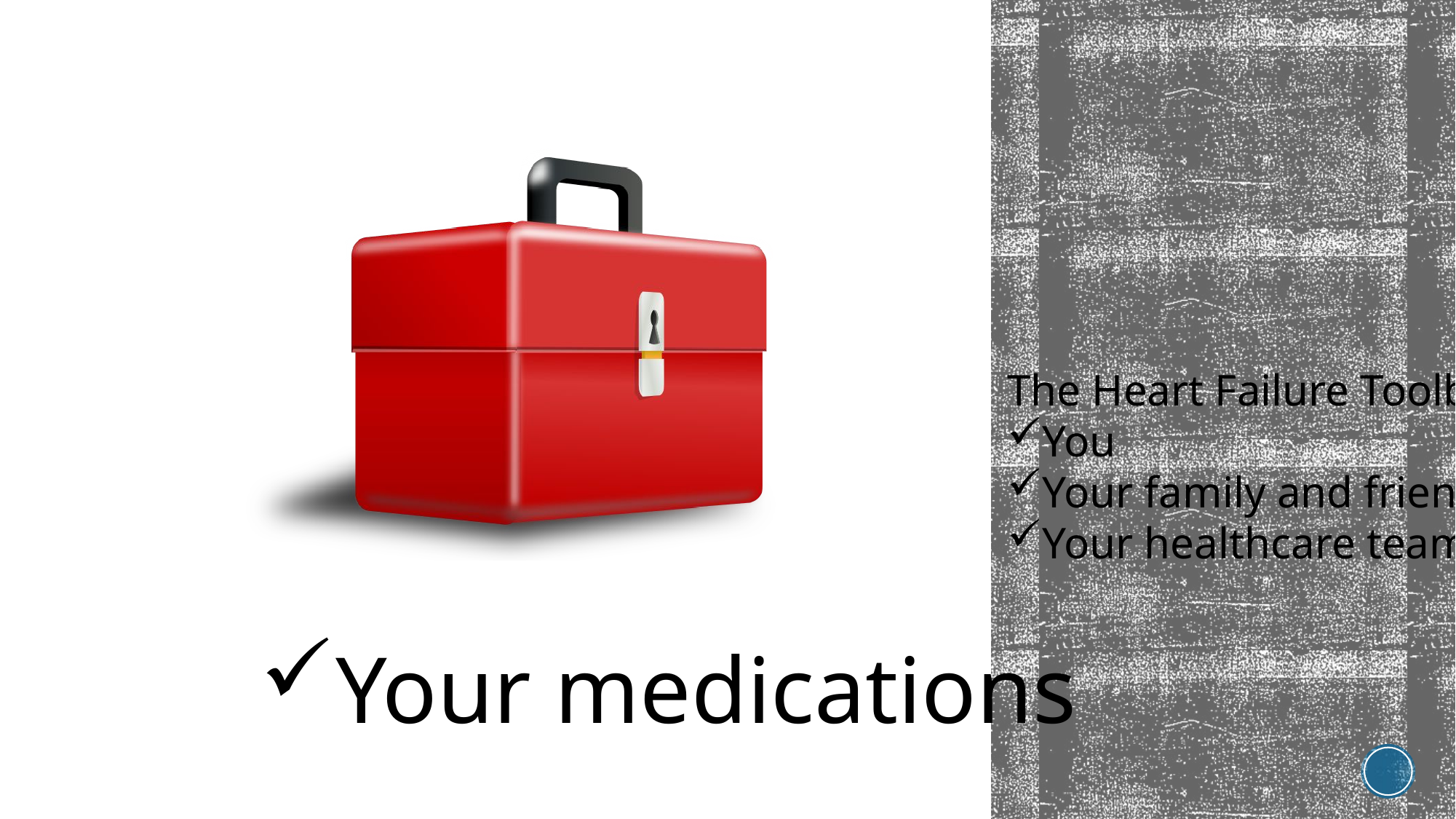

#
The Heart Failure Toolbox
You
Your family and friends
Your healthcare team
Your medications

## Slide 11
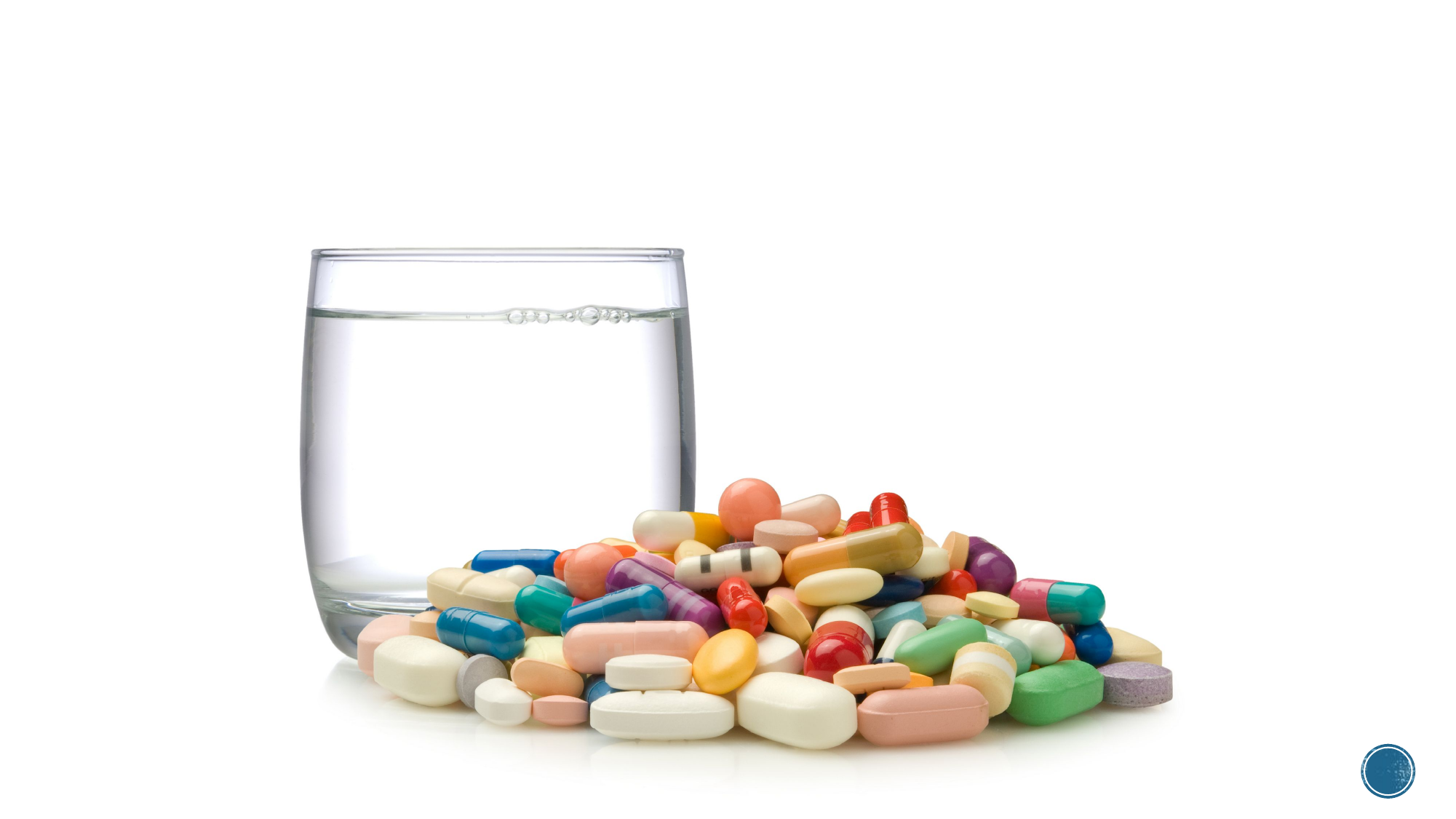

#

## Slide 12
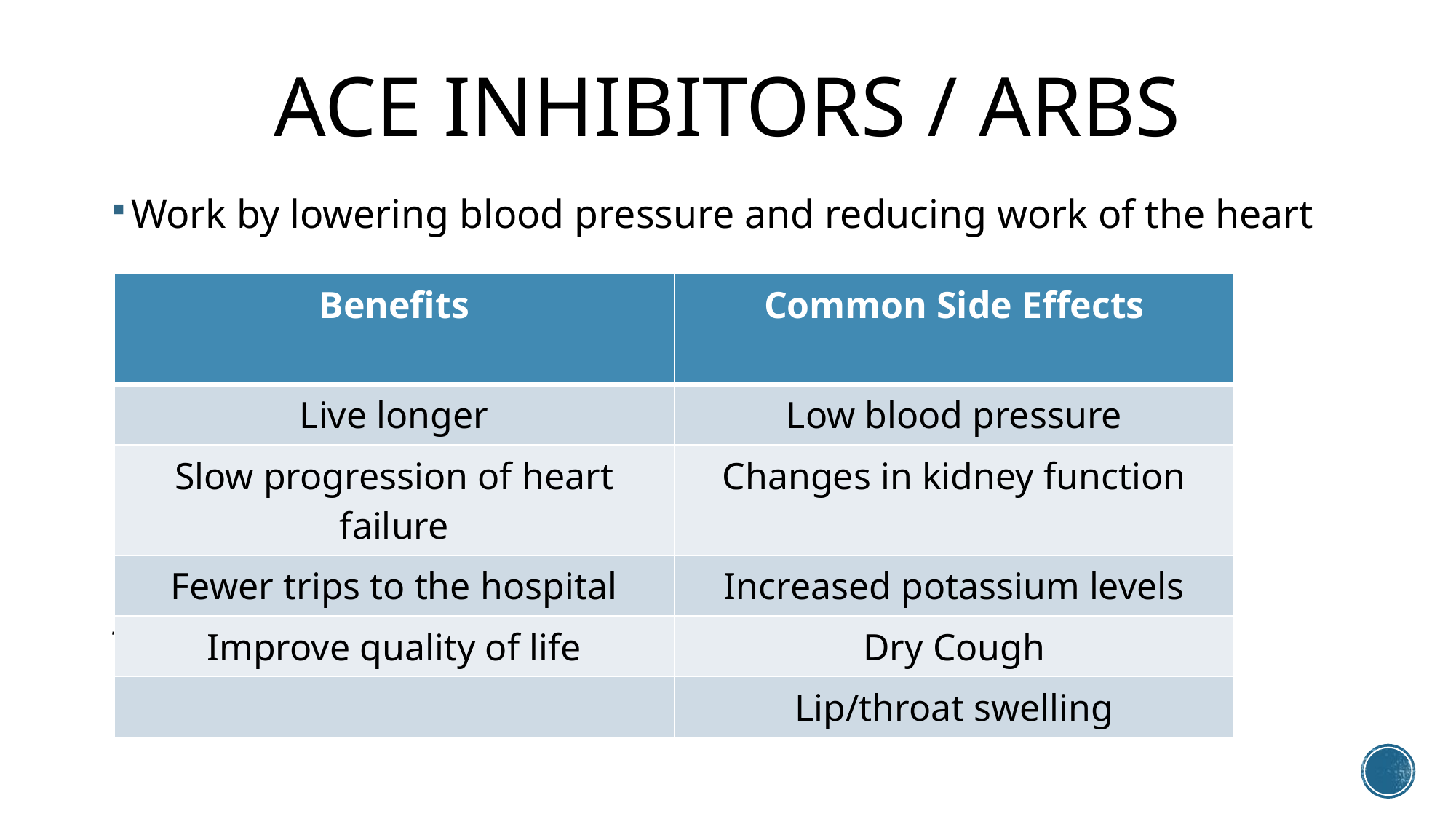

# ACE Inhibitors / ARBs
Work by lowering blood pressure and reducing work of the heart
**Caution not to use potassium salt substitutes unless instructed by provider**
| Benefits | Common Side Effects |
| --- | --- |
| Live longer | Low blood pressure |
| Slow progression of heart failure | Changes in kidney function |
| Fewer trips to the hospital | Increased potassium levels |
| Improve quality of life | Dry Cough |
| | Lip/throat swelling |

## Slide 13
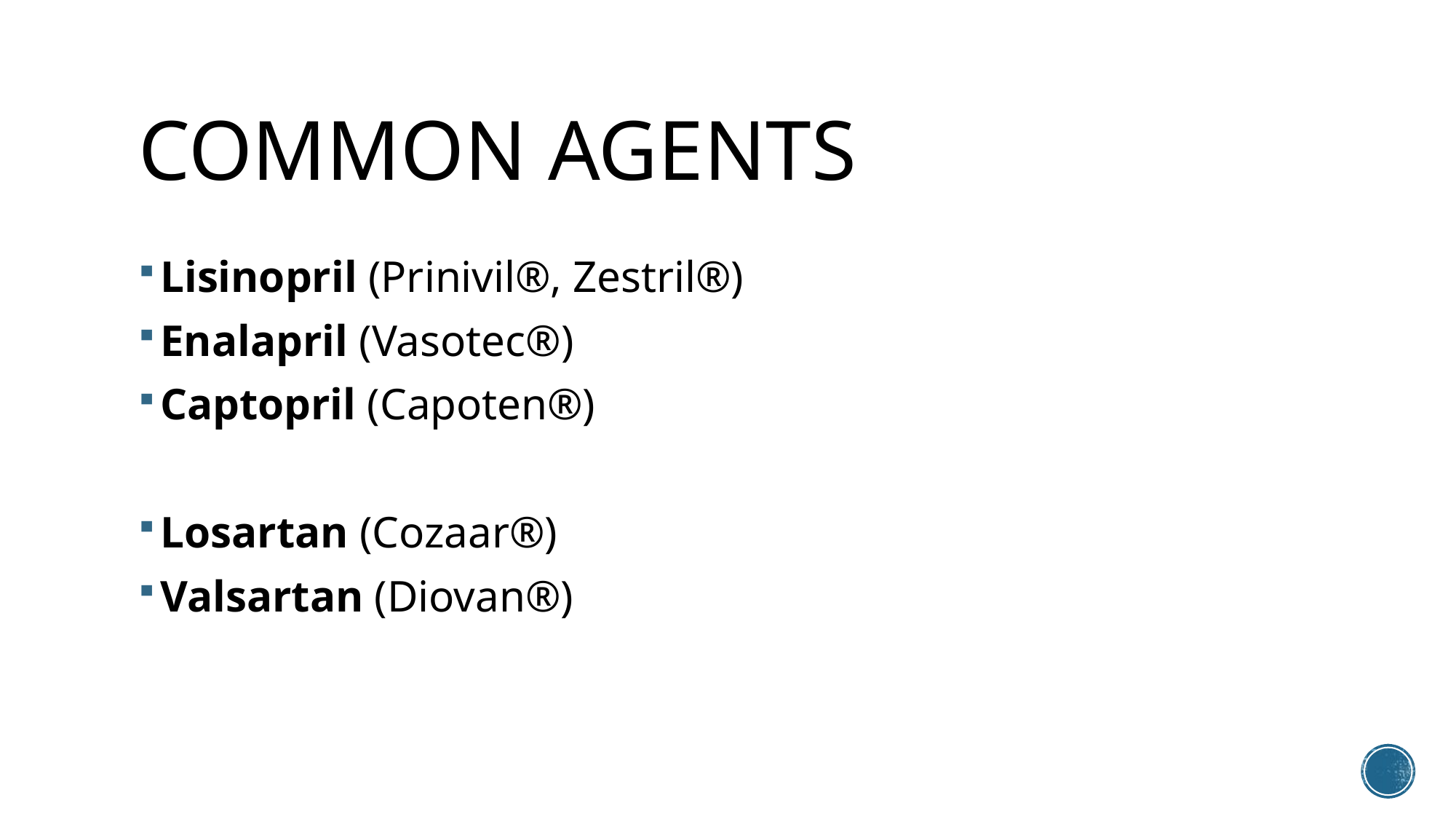

# Common Agents
Lisinopril (Prinivil®, Zestril®)
Enalapril (Vasotec®)
Captopril (Capoten®)
Losartan (Cozaar®)
Valsartan (Diovan®)

## Slide 14
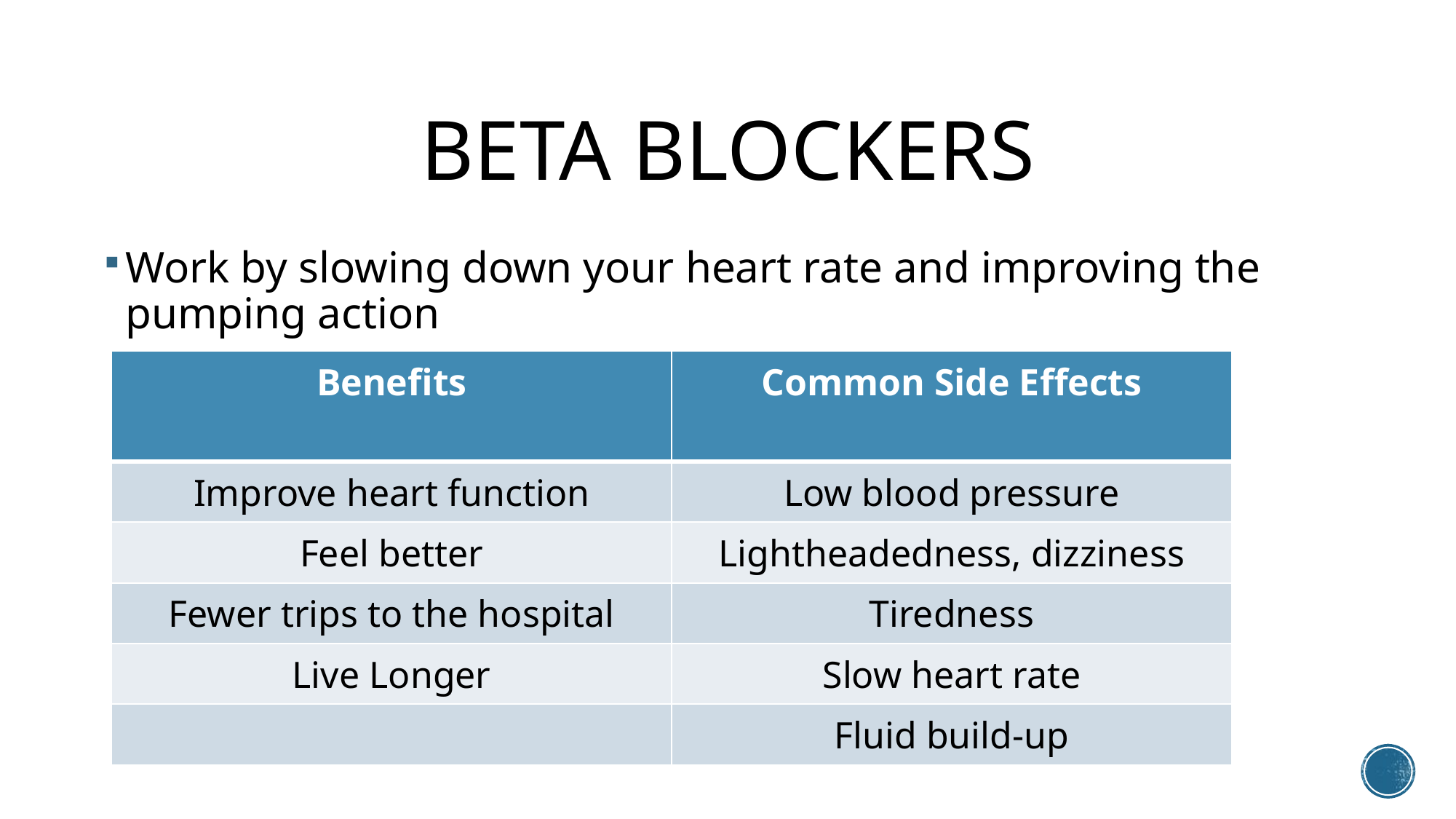

# Beta Blockers
Work by slowing down your heart rate and improving the pumping action
| Benefits | Common Side Effects |
| --- | --- |
| Improve heart function | Low blood pressure |
| Feel better | Lightheadedness, dizziness |
| Fewer trips to the hospital | Tiredness |
| Live Longer | Slow heart rate |
| | Fluid build-up |

## Slide 15
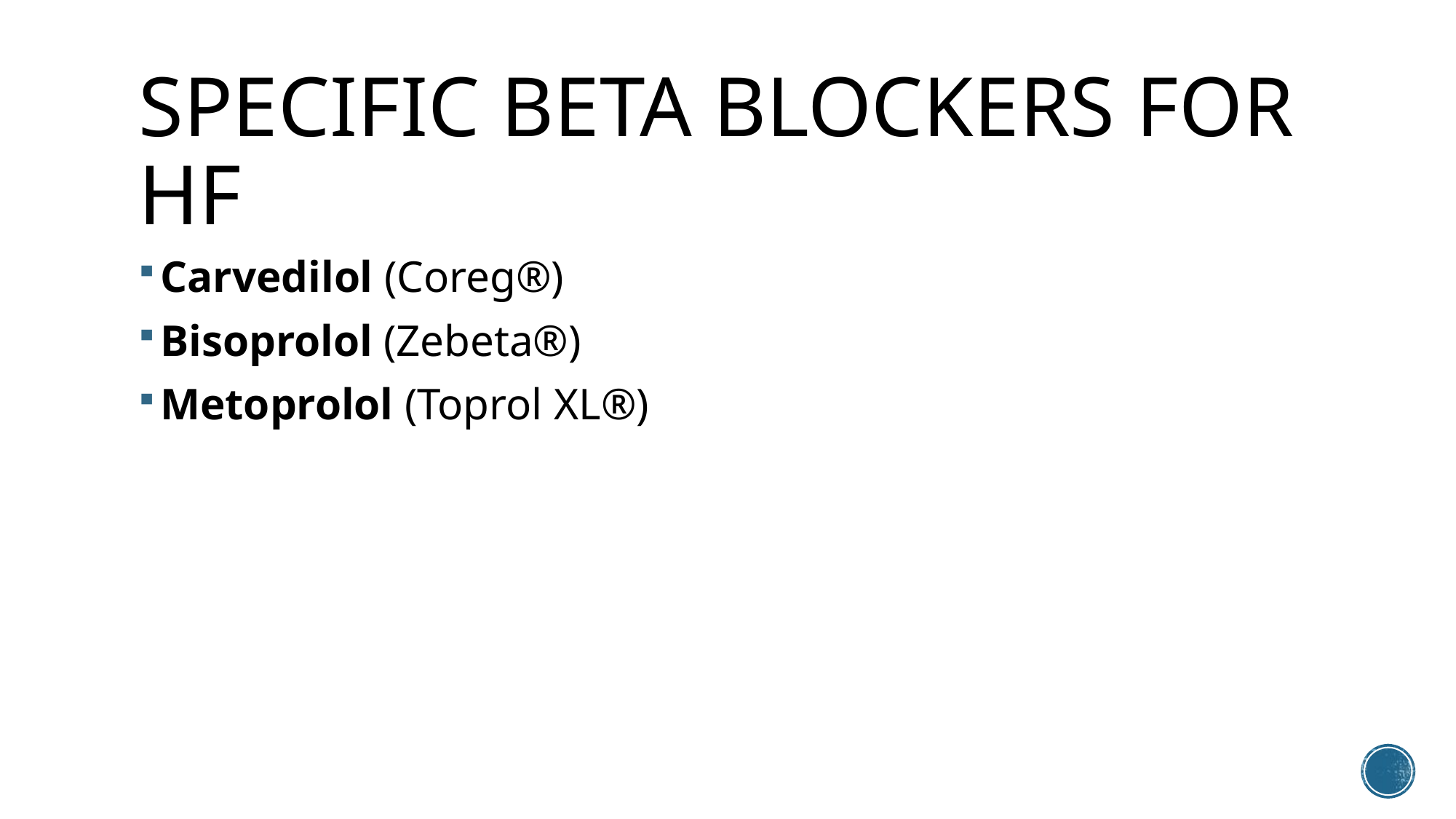

# Specific beta blockers for HF
Carvedilol (Coreg®)
Bisoprolol (Zebeta®)
Metoprolol (Toprol XL®)

## Slide 16
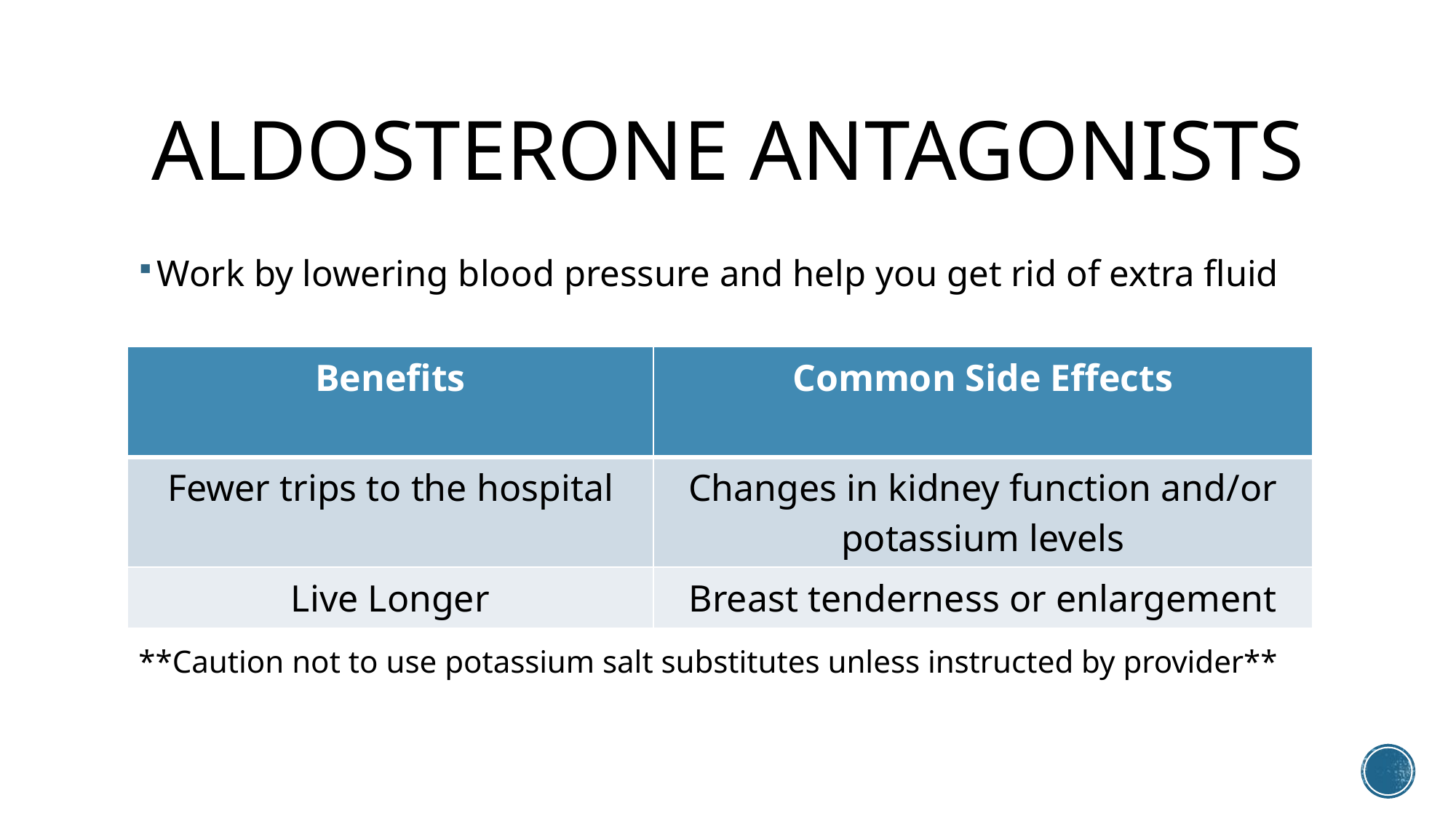

# Aldosterone AntagonistS
Work by lowering blood pressure and help you get rid of extra fluid
**Caution not to use potassium salt substitutes unless instructed by provider**
| Benefits | Common Side Effects |
| --- | --- |
| Fewer trips to the hospital | Changes in kidney function and/or potassium levels |
| Live Longer | Breast tenderness or enlargement |

## Slide 17
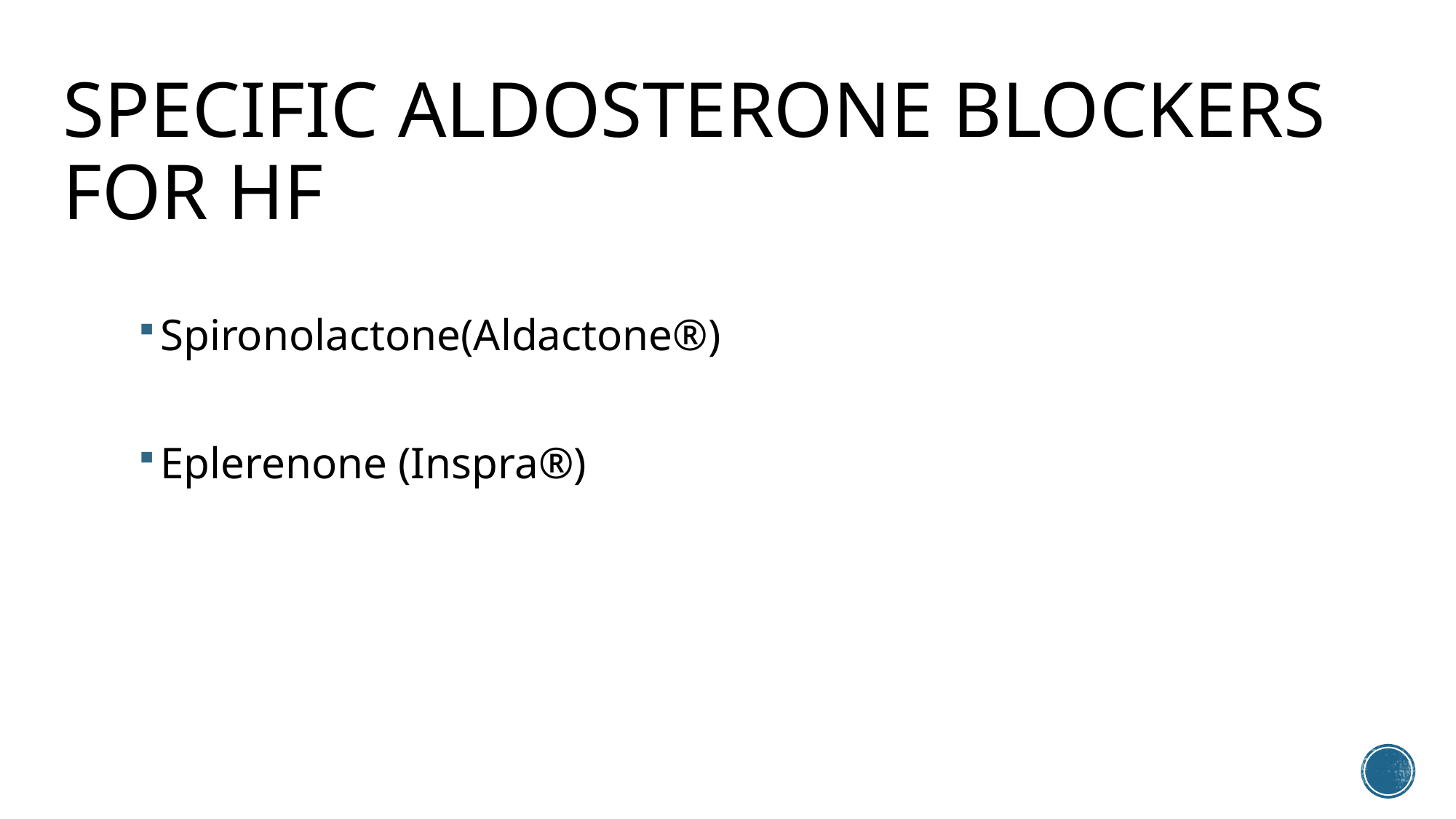

# Specific Aldosterone blockers for hf
Spironolactone(Aldactone®)
Eplerenone (Inspra®)

## Slide 18
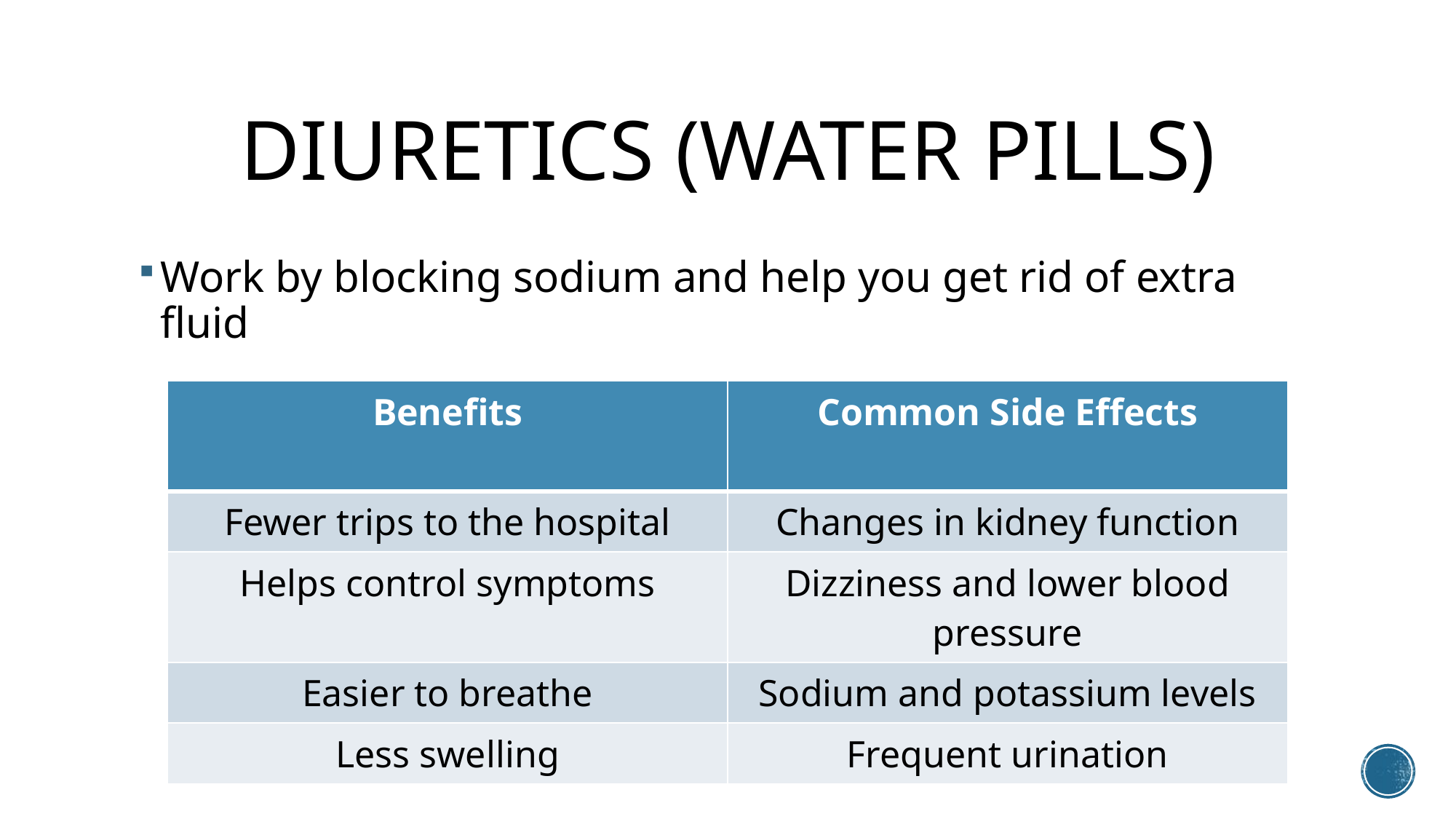

# Diuretics (Water Pills)
Work by blocking sodium and help you get rid of extra fluid
| Benefits | Common Side Effects |
| --- | --- |
| Fewer trips to the hospital | Changes in kidney function |
| Helps control symptoms | Dizziness and lower blood pressure |
| Easier to breathe | Sodium and potassium levels |
| Less swelling | Frequent urination |

## Slide 19
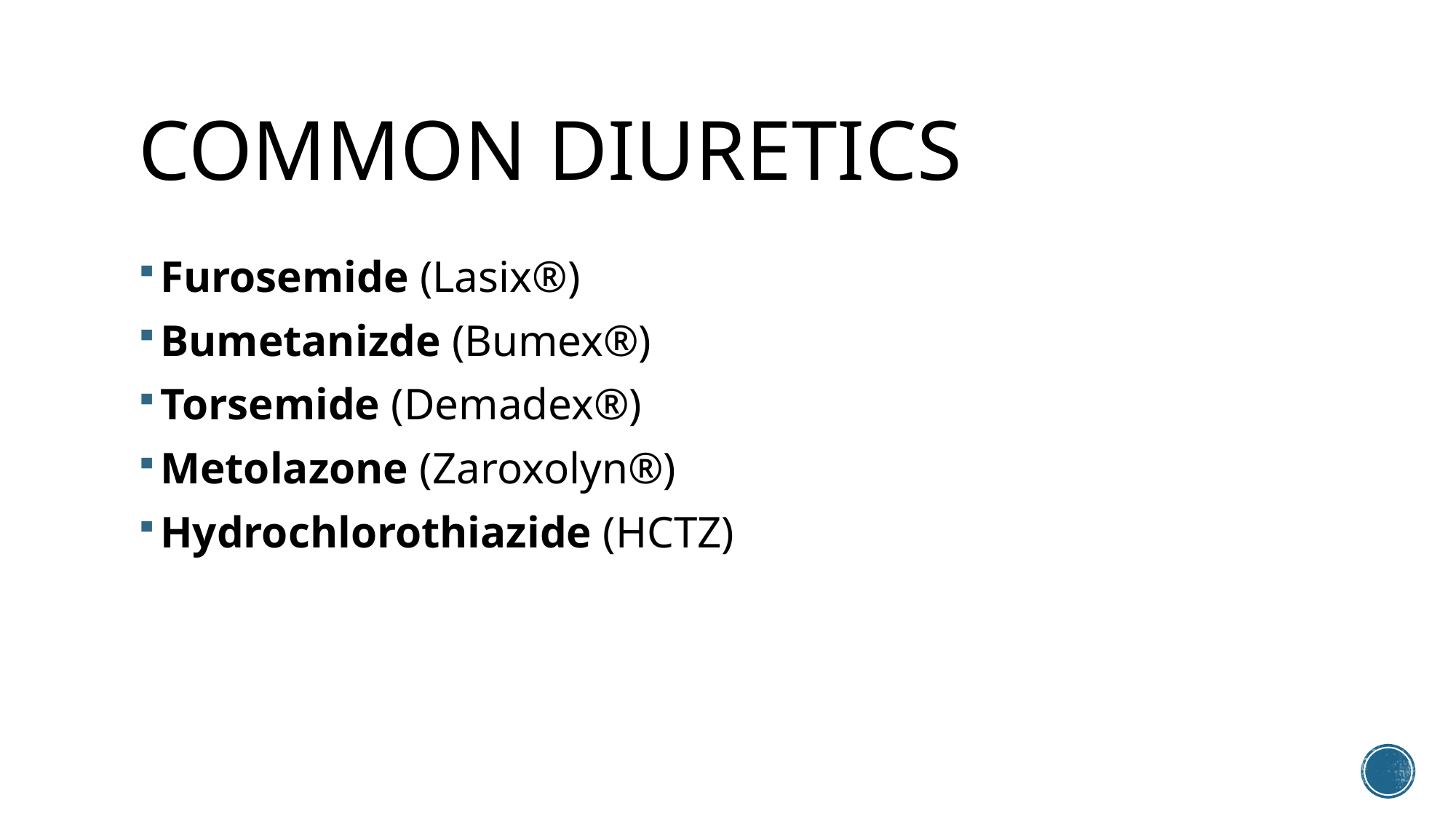

# Common diuretics
Furosemide (Lasix®)
Bumetanizde (Bumex®)
Torsemide (Demadex®)
Metolazone (Zaroxolyn®)
Hydrochlorothiazide (HCTZ)

## Slide 20
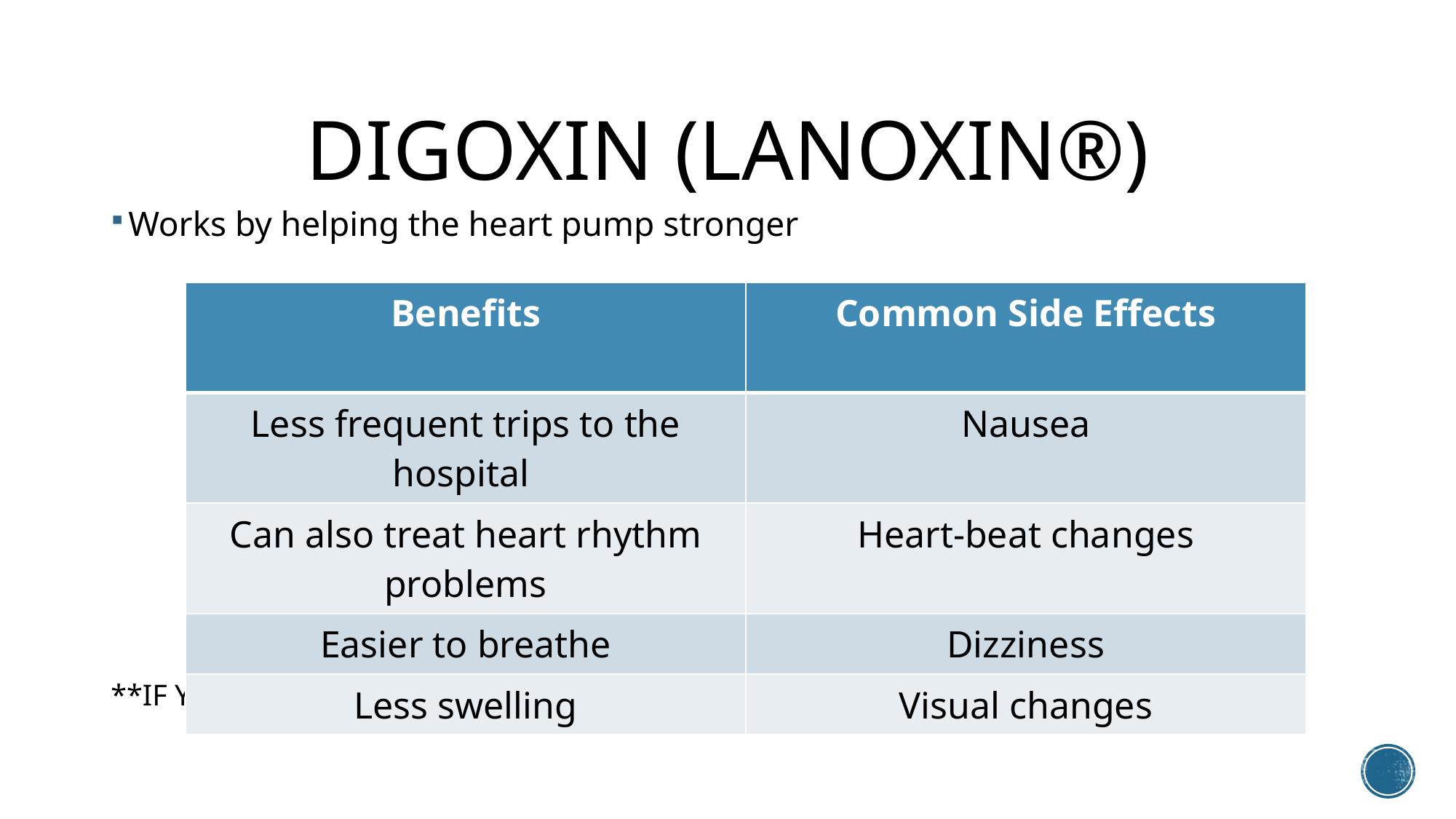

# Digoxin (Lanoxin®)
Works by helping the heart pump stronger
**IF YOU HAVE ANY SERIOUS SIDE EFFECTS CALL DOCTOR IMMEDIATELY**
| Benefits | Common Side Effects |
| --- | --- |
| Less frequent trips to the hospital | Nausea |
| Can also treat heart rhythm problems | Heart-beat changes |
| Easier to breathe | Dizziness |
| Less swelling | Visual changes |

## Slide 21
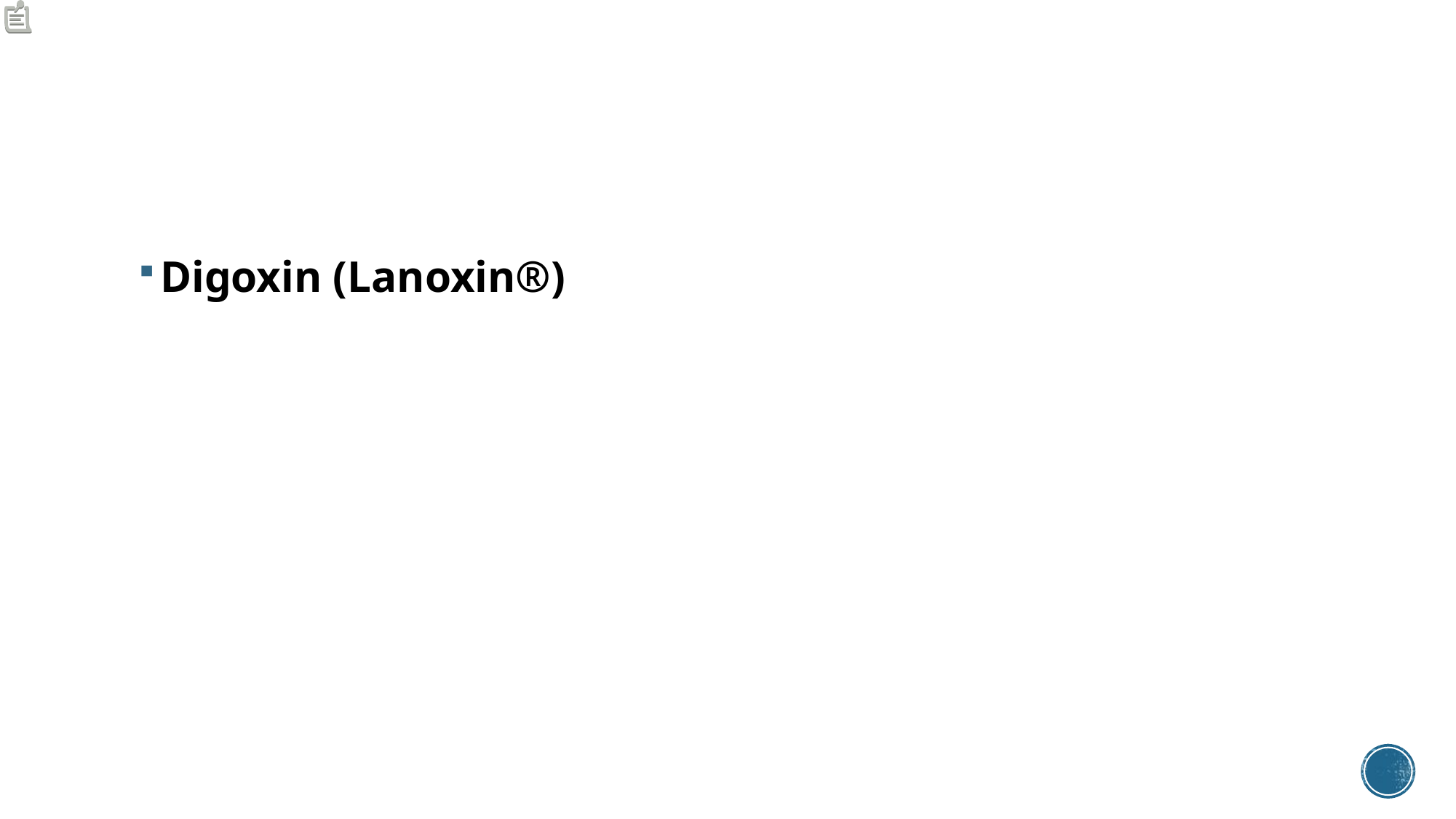

#
Digoxin (Lanoxin®)

## Slide 22
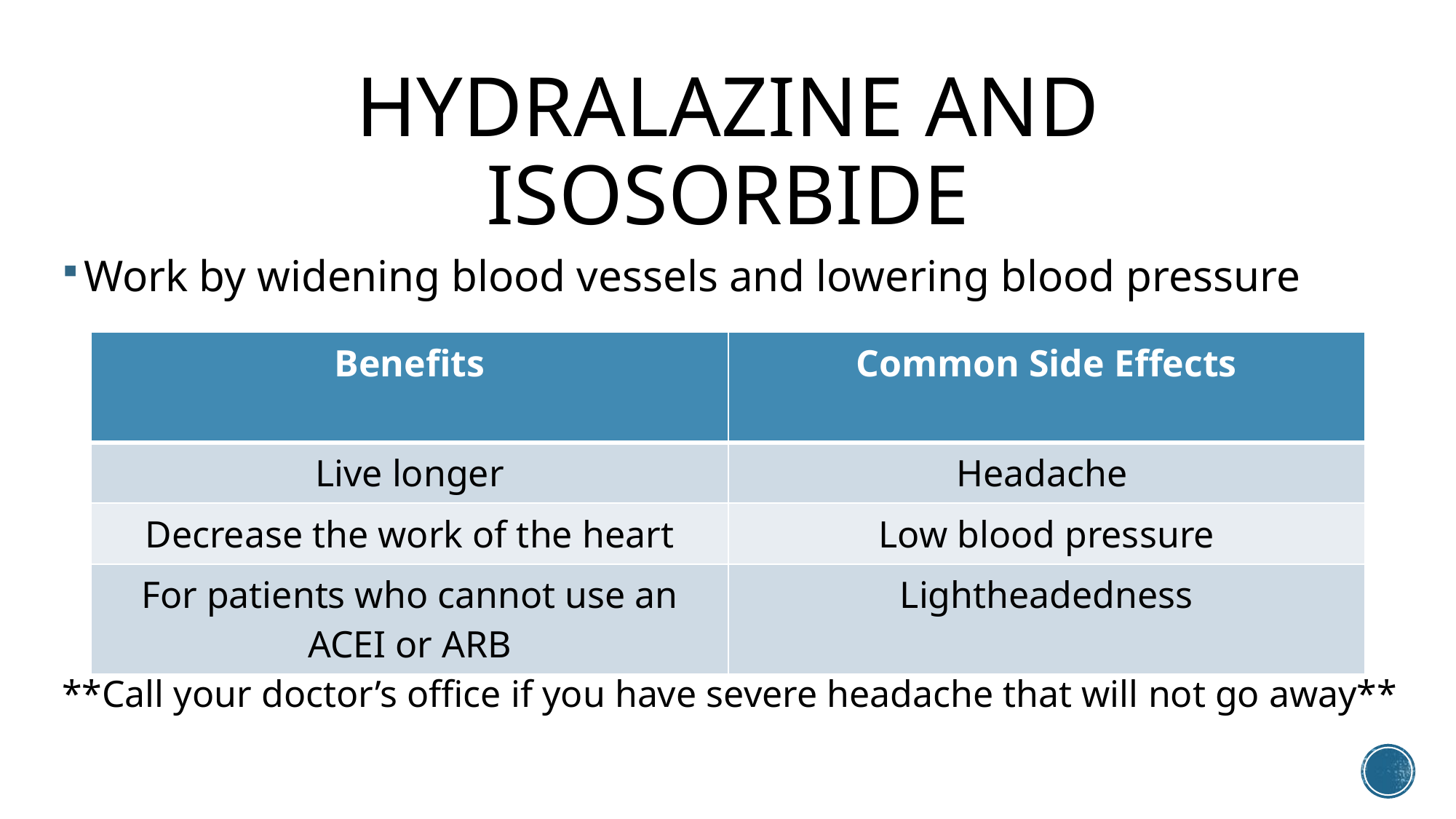

# Hydralazine and Isosorbide
Work by widening blood vessels and lowering blood pressure
**Call your doctor’s office if you have severe headache that will not go away**
| Benefits | Common Side Effects |
| --- | --- |
| Live longer | Headache |
| Decrease the work of the heart | Low blood pressure |
| For patients who cannot use an ACEI or ARB | Lightheadedness |

## Slide 23
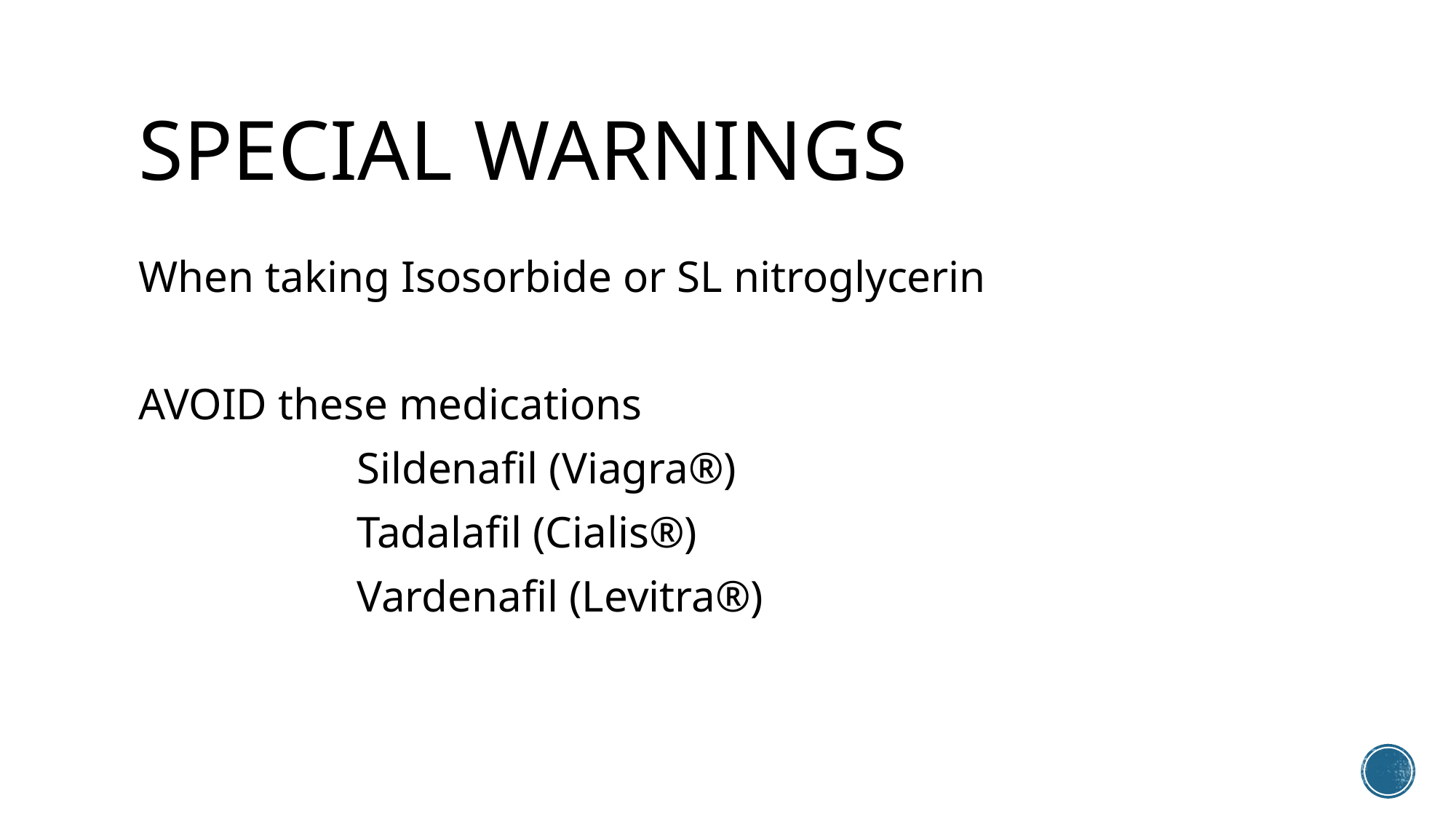

# Special Warnings
When taking Isosorbide or SL nitroglycerin
AVOID these medications
		Sildenafil (Viagra®)
		Tadalafil (Cialis®)
		Vardenafil (Levitra®)

## Slide 24
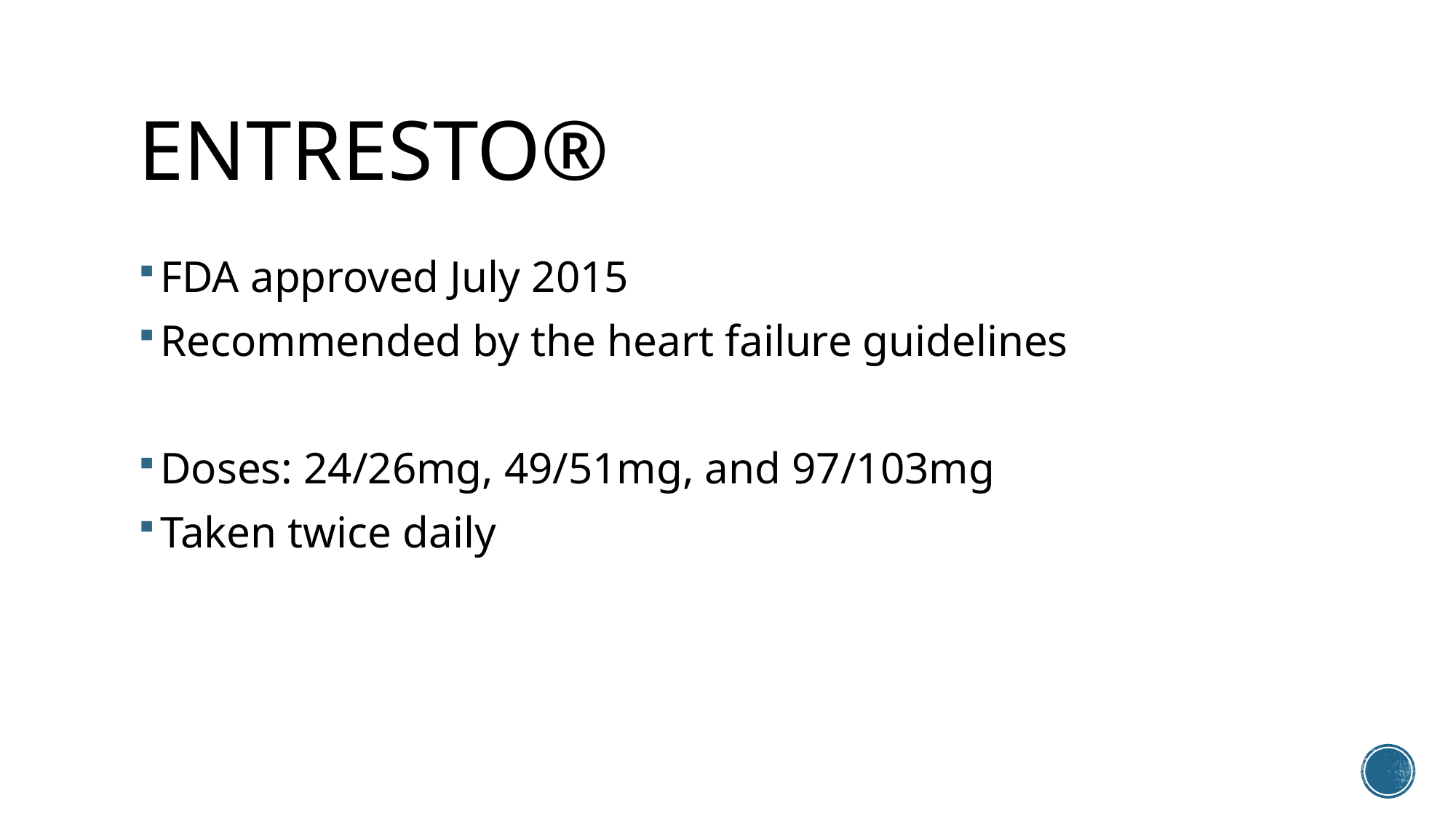

# Entresto®
FDA approved July 2015
Recommended by the heart failure guidelines
Doses: 24/26mg, 49/51mg, and 97/103mg
Taken twice daily

## Slide 25
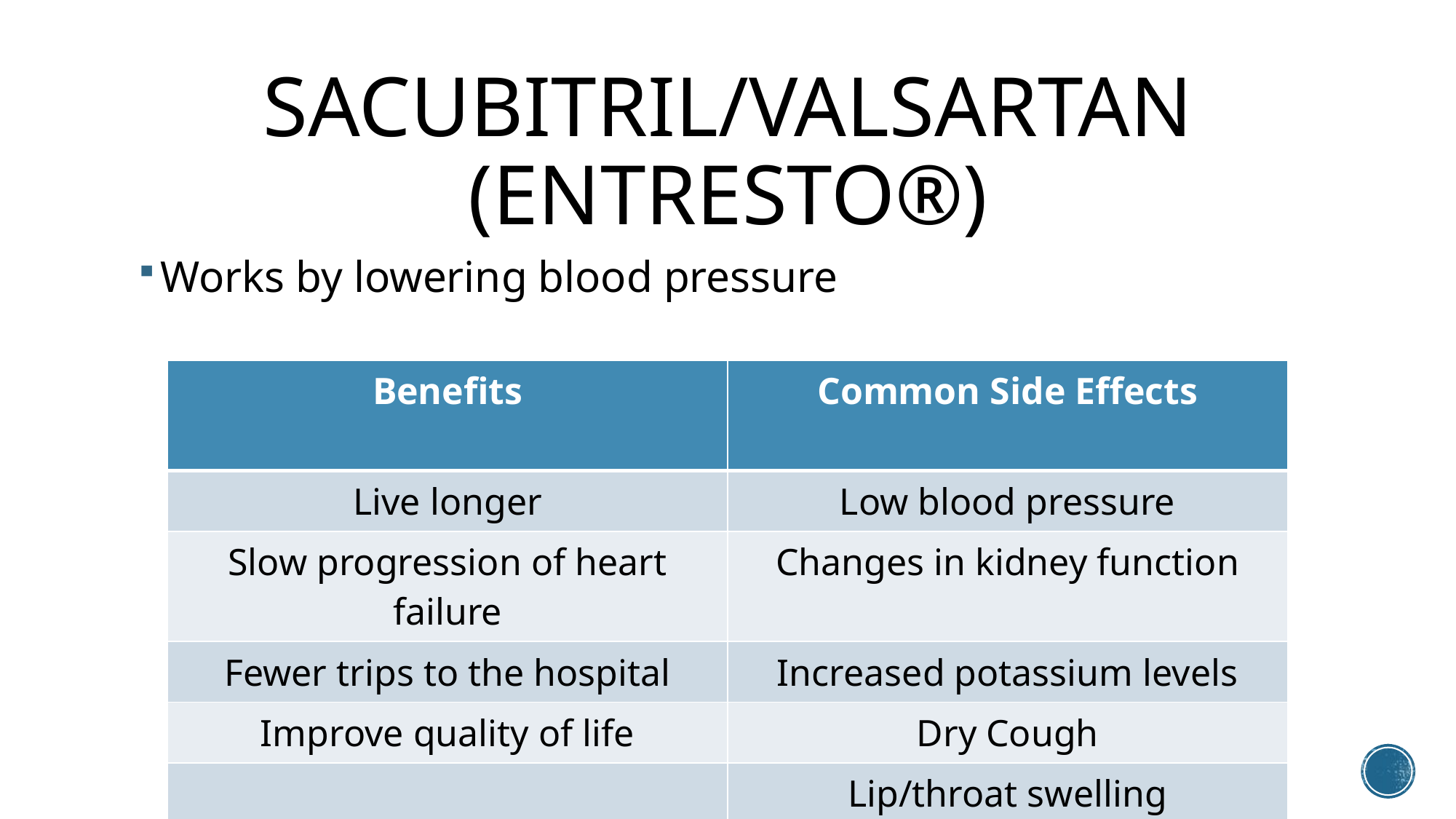

# Sacubitril/Valsartan (Entresto®)
Works by lowering blood pressure
| Benefits | Common Side Effects |
| --- | --- |
| Live longer | Low blood pressure |
| Slow progression of heart failure | Changes in kidney function |
| Fewer trips to the hospital | Increased potassium levels |
| Improve quality of life | Dry Cough |
| | Lip/throat swelling |

## Slide 26
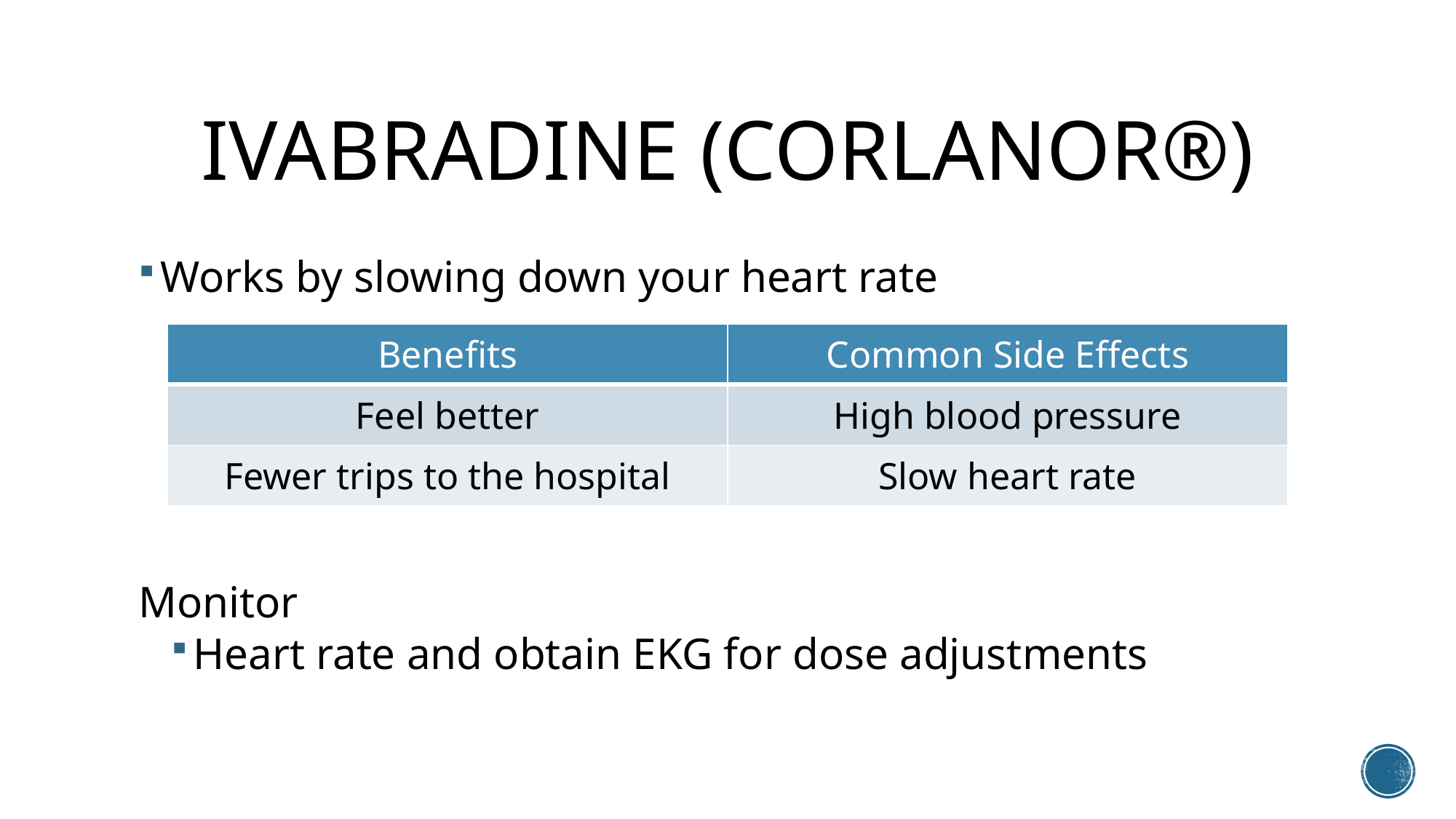

# Ivabradine (Corlanor®)
Works by slowing down your heart rate
Monitor
Heart rate and obtain EKG for dose adjustments
| Benefits | Common Side Effects |
| --- | --- |
| Feel better | High blood pressure |
| Fewer trips to the hospital | Slow heart rate |

## Slide 27
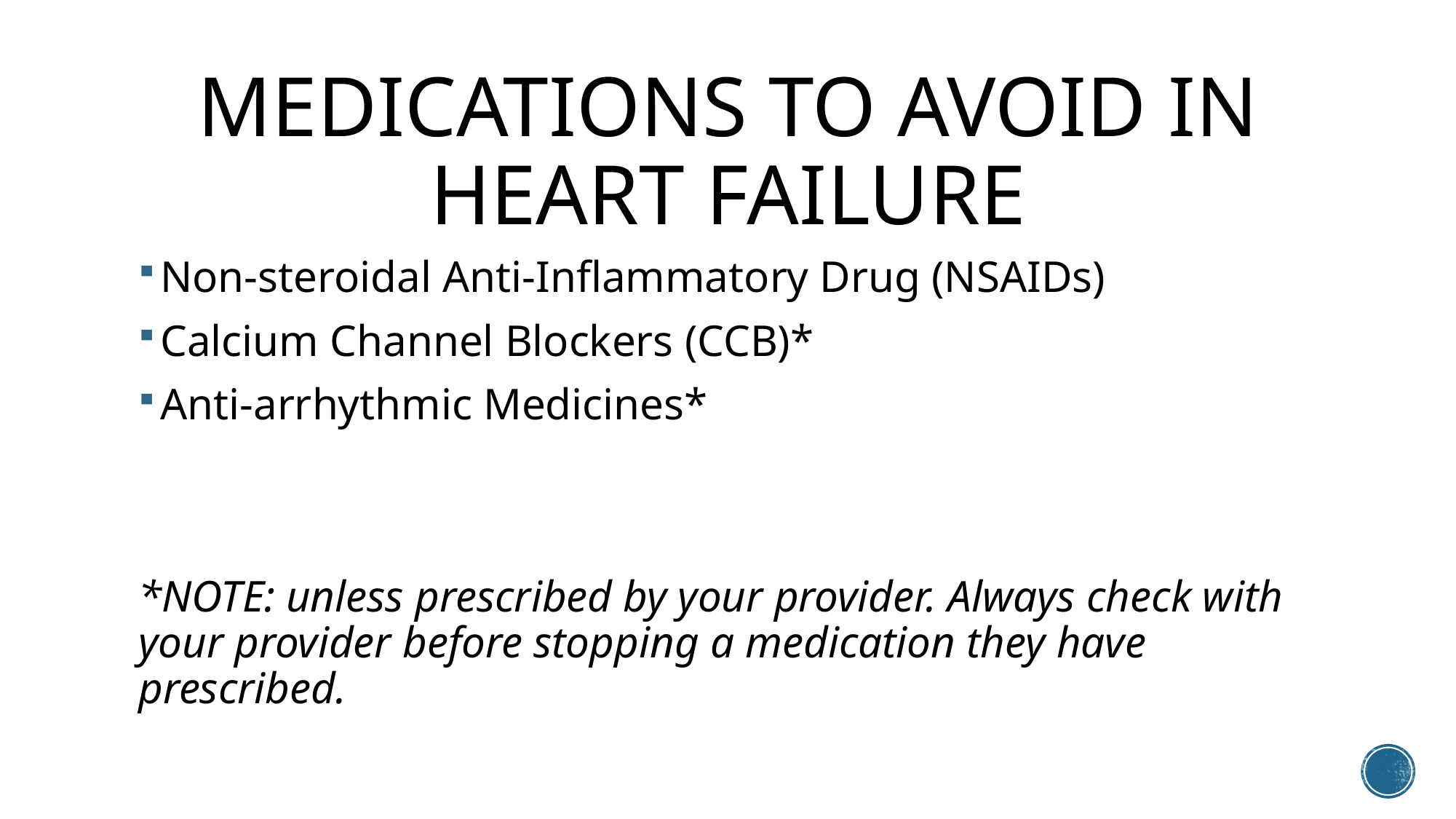

# Medications to Avoid in Heart Failure
Non-steroidal Anti-Inflammatory Drug (NSAIDs)
Calcium Channel Blockers (CCB)*
Anti-arrhythmic Medicines*
*NOTE: unless prescribed by your provider. Always check with your provider before stopping a medication they have prescribed.

## Slide 28
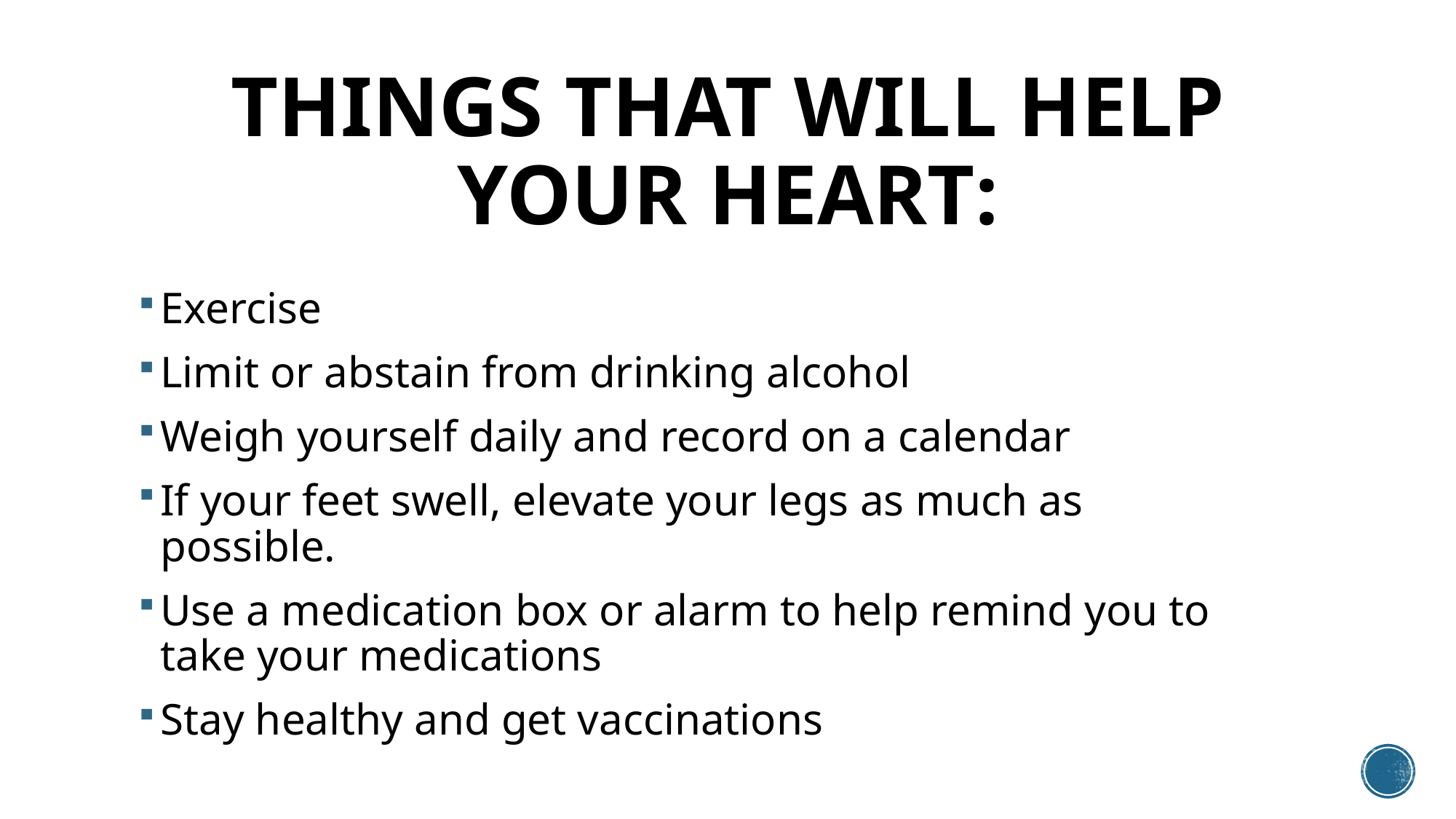

# Things that will help your Heart:
Exercise
Limit or abstain from drinking alcohol
Weigh yourself daily and record on a calendar
If your feet swell, elevate your legs as much as possible.
Use a medication box or alarm to help remind you to take your medications
Stay healthy and get vaccinations

## Slide 29
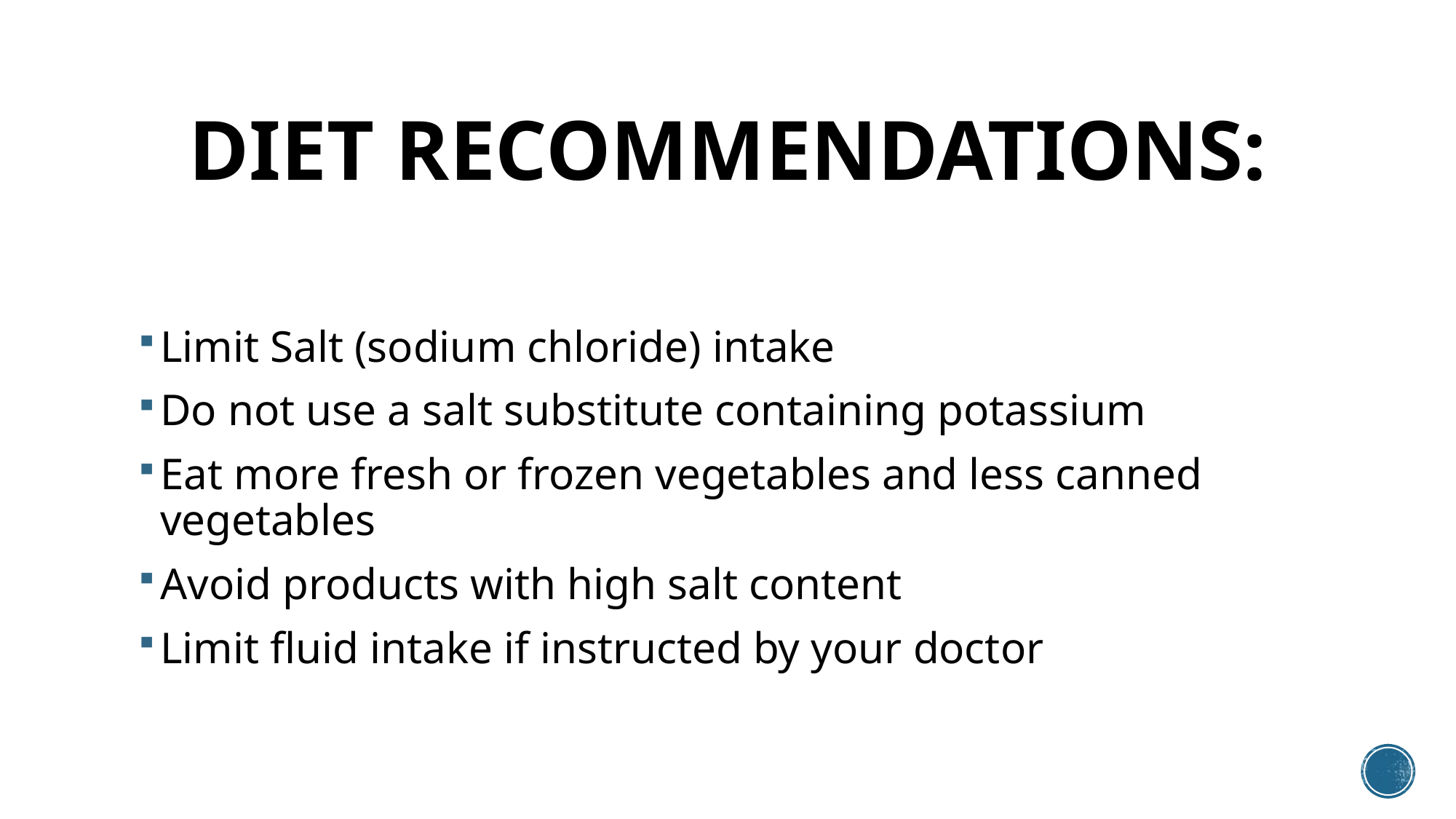

# Diet Recommendations:
Limit Salt (sodium chloride) intake
Do not use a salt substitute containing potassium
Eat more fresh or frozen vegetables and less canned vegetables
Avoid products with high salt content
Limit fluid intake if instructed by your doctor

## Slide 30
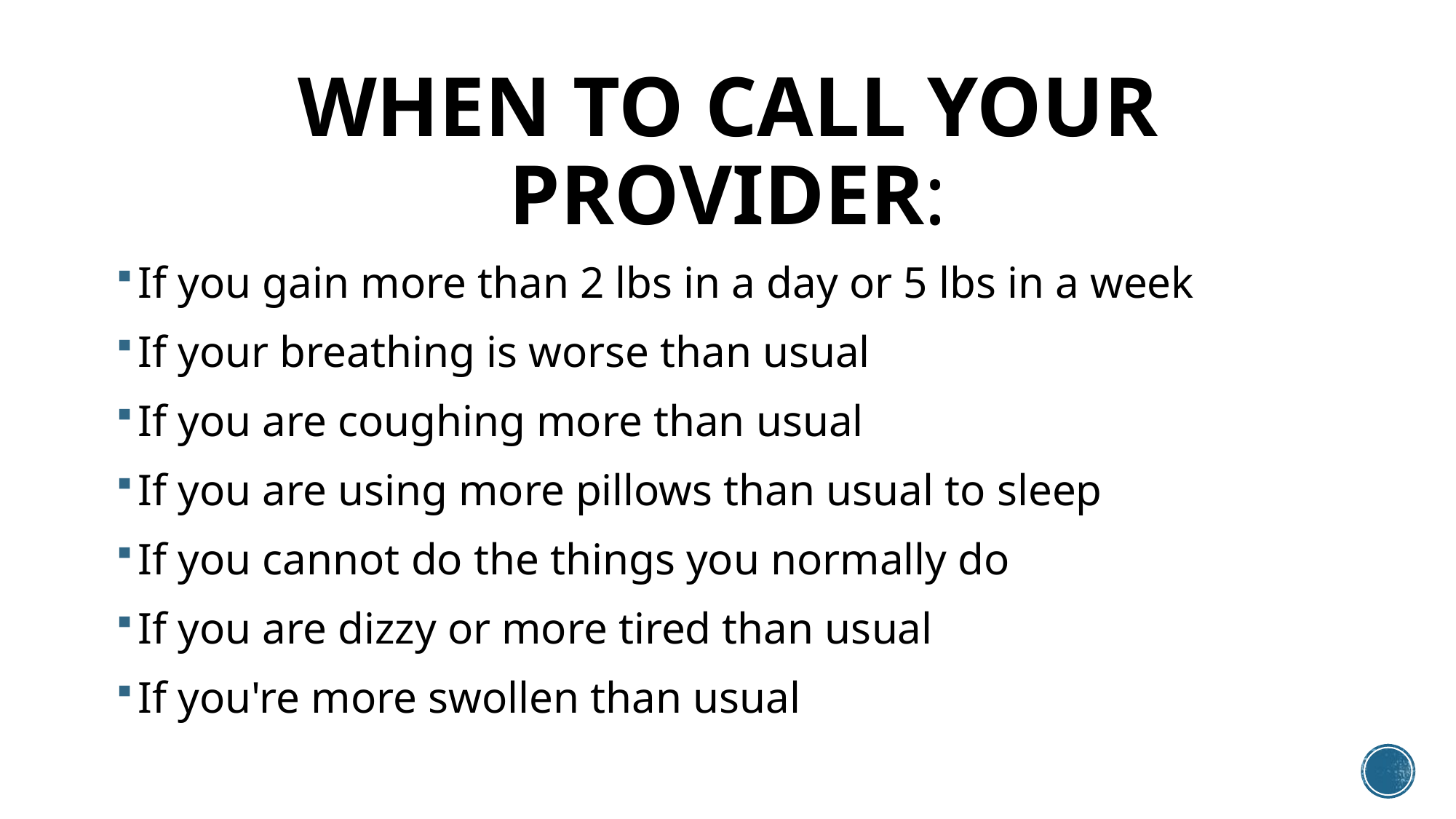

# When to Call Your Provider:
If you gain more than 2 lbs in a day or 5 lbs in a week
If your breathing is worse than usual
If you are coughing more than usual
If you are using more pillows than usual to sleep
If you cannot do the things you normally do
If you are dizzy or more tired than usual
If you're more swollen than usual

## Slide 31
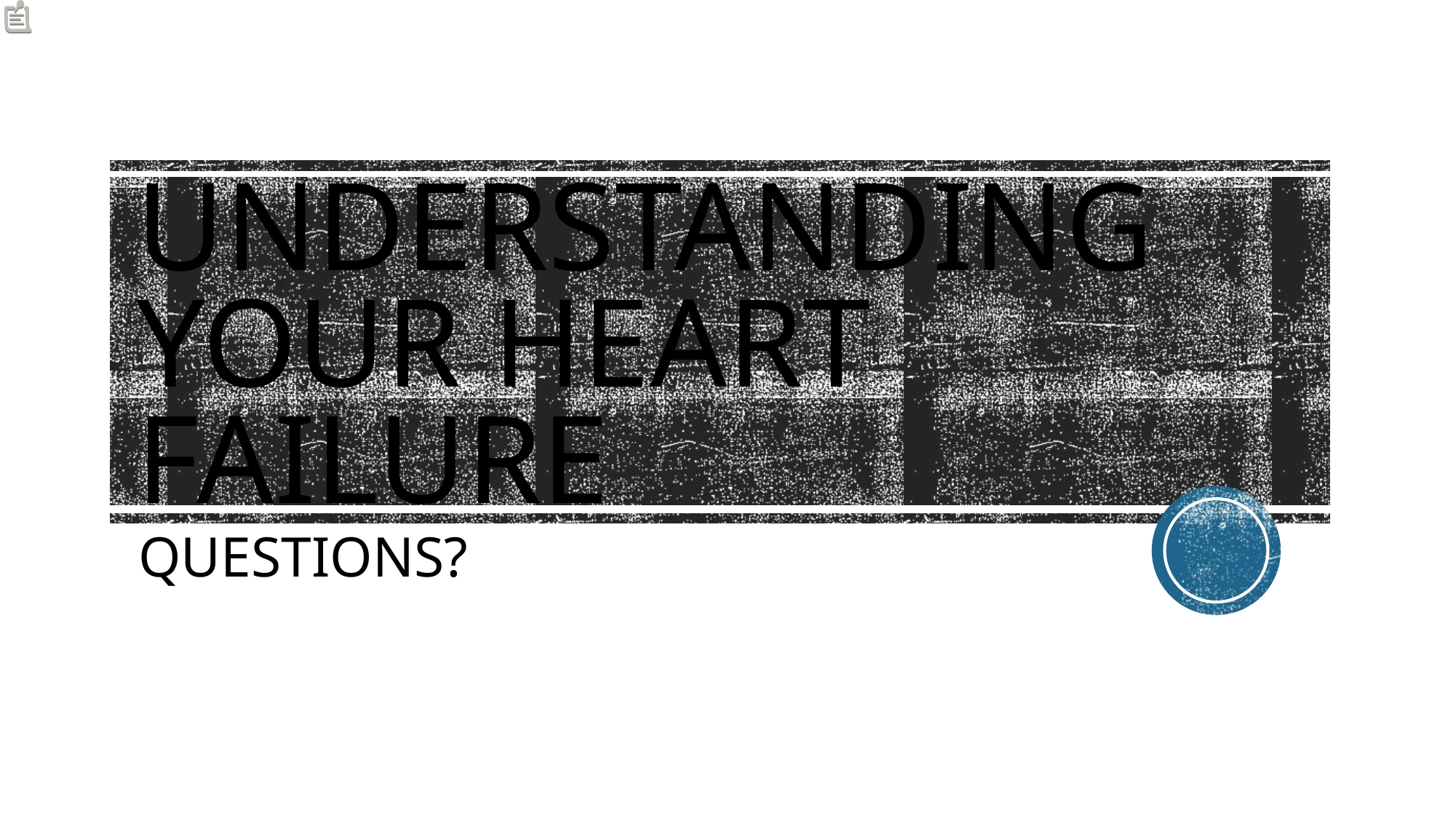

# Understanding your Heart Failure
QUESTIONS?
